# A benchmark of gene expression tissue-specificity metrics

**DOI:** 10.1093/bib/bbw008

**Published:** 2016-02-18

**Authors:** Nadezda Kryuchkova-Mostacci, Marc Robinson-Rechavi

**Affiliations:** 1 Department of Ecology and Evolution, University of Lausanne, Lausanne, Switzerland; 2 Swiss Institute of Bioinformatics, Lausanne, Switzerland

**Keywords:** tissue specificity, expression, human, mouse, RNA-seq, microarray

## Abstract

One of the major properties of genes is their expression pattern. Notably, genes are often classified as tissue specific or housekeeping. This property is of interest to molecular evolution as an explanatory factor of, e.g. evolutionary rate, as well as a functional feature which may in itself evolve. While many different methods of measuring tissue specificity have been proposed and used for such studies, there has been no comparison or benchmarking of these methods to our knowledge, and little justification of their use. In this study, we compare nine measures of tissue specificity. Most methods were established for ESTs and microarrays, and several were later adapted to RNA-seq. We analyse their capacity to distinguish gene categories, their robustness to the choice and number of tissues used and their capture of evolutionary conservation signal.

## Introduction

Gene expression analysis is widely used in genomics and measured with microarrays or RNA-seq. In the case of a multicellular organism with different tissues, it is often useful to have a measure of how tissue specific a gene is.

Even if tissue specificity is often used in studies, there is usually no clear answer why one or another method was used. Yet, there are several methods to measure gene specificity, which differ in their assumptions and their scale. The simplest one is to count in how many tissues each gene is expressed (used in e.g. [1–6]). The problem of this method is to define the threshold to call a gene expressed. Originally, with expressed sequence tags (ESTs), a count of 1 EST was considered sufficient [2]. There are different methods to define thresholds for microarrays [7], while for RNA-seq, an Reads Per Kilobase per Million mapped reads (RPKM) value of 1 is generally used [8, 9]. Some studies use a stringent threshold, e.g. signal to noise ratio >10 [10], and count a gene as specific only if expressed in a single tissue. This method causes only highly expressed genes to be taken into account, and if a data set contains closely related tissues (e.g. brain parts), less genes are called tissue specific. Other papers a very low threshold, e.g. 0.3 RPKM [11–13], that leads to defining most genes as housekeeping.

A widely used method which does not depend on such a cut-off in its formula is Tau [14] (for details, see ‘Materials and Methods’). Tau varies from 0 to 1, where 0 means broadly expressed, and 1 is specific (used in e.g. [15–24]).

Other methods have been proposed, such as the expression enrichment (EE) [25], to calculate for which tissue each gene is specific, for example, in the database TiGER [26]. We also considered: the tissue specificity index (TSI) [27] (used in e.g. [28–30]); Hg by Schug *et al.* [31]; the z-score (used in [32]), widely used for other features than tissue specificity; SPM, used in the database TiSGeD [33]; and Preferential Expression Measure (PEM), suggested for ESTs by Huminiecki *et al.* [34] and used in e.g. [35–38]. Finally, the Gini coefficient, widely used in economics to measure inequality [39], was compared with methods originating in biology.

These methods can be divided in two groups. One group summarizes in a single number whether a gene is tissue specific or ubiquitously expressed (Tau, Gini, TSI, Counts and Hg), and the second group shows for each tissue separately how specific the gene is to that tissue (z-score, SPM, EE and PEM). For comparison purposes with the first group, we use the maximum specificity from the second group.

## Material and methods

For all equations,

$$x_{i}\;is\;the\;expression\;of\;the\;gene\;in\;tissue\;i$$

n  is  the  number  of  tissues

The method of counting in how many tissues a gene is expressed was simply calculated as follows:

Counts=#  tissues  expressed

A cut-off needs to be set; the cut-offs that we used are explained at the end of the ‘Methods' section.

While the other methods do not necessitate a cut-off per their mathematical formulation, they need positive expression values. As expression values are usually log-transformed (because they are log-normally distributed), this means that values <1 are not manageable. Solutions include using a multiplier or/and setting a cut-off of 1 before log transformation. For details of our treatment of the data, see the description of RNA-seq and microarray data.

Tau was calculated as follows [14]:

$$\tau =\frac{\sum_{i=1}^{n}\left(1-\hat{x_{i}}\right)}{n-1};\;\hat{x_{i}}=\frac{x_{i}}{\underset{1\leq i\leq n}{\max}\left(x_{i}\right)}.$$

The EE score was calculated as follows [25]:

$$EE=\frac{x_{i}}{\sum_{i=1}^{n}x_{i}*\frac{s_{i}}{\sum_{i=1}^{n}s_{i}}}=\frac{\sum_{i=1}^{n}s_{i}}{s_{i}}*\frac{x_{i}}{\sum_{i=1}^{n}x_{i}}s_{i}\;summary\;of\;the\;expression\;of\;all\;genes\;in\;tissue\;i.$$

TSI was calculated as follows [27]:

$$TSI=\;\frac{\underset{1\leq i\leq n}{\max}\left(x_{i}\right)}{\sum_{i=1}^{n}x_{i}}.$$

The Gini coefficient was calculated as follows:

$$Gini=\frac{n+1}{n}-\frac{2\sum_{i=1}^{n}(n+1-i)x_{i}}{n\sum_{i=1}^{n}x_{i}}x_{i}\;has\;to\;be\;ordered\;from\;least\;to\;greatest.$$

Hg [31] was calculated as follows:

$$H_{g}=-\sum_{i=1}^{n}p_{i}\text{*}\log_{2}\left(p_{i}\right);\;p_{i}=\frac{x_{i}}{\sum_{i=1}^{n}x_{i}}.$$

The z-score was calculated as follows:

$$\begin{array}{c} z=\;\frac{x_{i}-\mu }{\sigma }\\ \mu \;is\;the\;mean\;of\;gene\;expression;\;\sigma \;is\;the\;standard\;deviatio\mathrm{n.} \end{array}\;$$

The z-score can be implemented in two ways: either only over-expressed genes are defined as tissue specific, or the absolute distance from the mean is used, so that under-expressed genes are also defined as tissue specific. Only the former method was used to be able to compare z-score with other methods.

SPM from the database TiSGeD [33] was calculated as follows:

$$SPM=\frac{x_{i}^{2}}{\sum_{i=1}^{n}x_{i}^{2}}.$$

PEM estimates how different the expression of the gene is relative to an expected expression, under the assumption of uniform expression in all tissues. PEM is calculated as follows [34]:

$$PEM=\log_{10}(\frac{\sum_{i=1}^{n}s_{i}}{s_{i}}*\frac{x_{i}}{\sum_{i=1}^{n}x_{i}})s_{i}\;summary\;of\;the\;expression\;of\;all\;genes\;in\;tissue\;i.$$

Derivation of all the methods from the original equations is presented in Supplementary Materials.

The output of all methods was modified to the same scale from 0 (ubiquitous) to 1 (tissue specific) to be able to compare them (Table 1). Four of the methods calculate specificity value for each tissue separately; for these methods, the largest (most specific) value among all tissues was assigned to the gene (see Table 1).

**Table 1.** Tissue specificity parameters. *N* is the number of tissues in the data set

Methods	Tissues	Ubiquitous	Specific	Transformation
τ (tau)	all	0	1	–
Gini	all	0	(N−1)/N	x*(N/(N−1))
TSI	all	0	1	–
Counts	all	*N*	1	(1−x/N)*(N/(N−1))
$EE_{i}$	separately	0	>5	X/maxX
Hg	all	$\log_{2}N$	0	$1-x/\log_{2}N$
Z score	separately	0	>3	$X/n-1/\sqrt{N}$
PEM score	separately	0	∼1	X/max X
SPM	separately	0	1	X

$X=\;\underset{1\leq i\leq n}{\max}x_{i}$ is the maximal specificity value for a certain gene among all tissues.

All the methods were compared using R version 3.2.1 [40], with the gplots [41], reldist [42, 43], VennDiagram [44] and preprocessCore libraries [45]; the R script is available in Supplementary Materials.

We used the following RNA-seq data: 27 human tissues (E-MTAB-1733) from Fagerberg *et al.* [46] downloaded from their Supplementary Materials, 22 mouse tissues (GSE36025) from the ENCODE project [47, 48] as used in Kryuchkova-Mostacci and Robinson-Rechavi [20] and 8 human tissues and 6 mouse tissues from Brawand *et al.* [49], as processed in the Bgee database [50]. All the genes with expression <1 RPKM were set as not expressed. The RNA-seq data were first log-transformed. After the normalization, a mean value from all replicates for each tissue separately was calculated. All genes that were not expressed in at least one tissue were removed from the analysis.

We used the following microarray data, as annotated in the Bgee database: 32 human tissues (GSE2361) [51] and 19 mouse tissues (GSE9954) [52]. Of note, on the microarrays, we have only 9788 (resp. 16  043) genes with data in human (resp. mouse), relative to 18  754 (resp. 27  364) for RNA-seq. For the microarray data, we used the logarithm of normalized signal intensity. The values set as absent in Bgee were set to 0, following the method of Schuster *et al.* [53]. After the normalization, a mean value from all replicates for each tissue separately was calculated. All genes that were not expressed in at least one tissue were removed from the analysis.

A summary of the workflow is presented in Supplementary Figure S1.

For the comparison of tissue-specific or ubiquitous gene functions, we used the following Gene Ontology (GO) terms: spermatogenesis (GO:0007283; expected to be specific to testis; 469 human genes), neurological system process (GO:0050877; expected to be specific to brain and other neural tissues; 1338 human genes), xenobiotic metabolic process (GO:0006805; expected to be specific to liver and kidney; 163 human genes), protein folding (GO:0006457; expected to be ubiquitous; 231 human genes), membrane organization (GO:0061024; expected to be ubiquitous; 607 human genes) and RNA splicing (GO:0008380; expected to be ubiquitous; 383 human genes).

GO enrichment analysis was performed with GOrilla [54] and Revigo [55].

## Results

**Figure 1.**
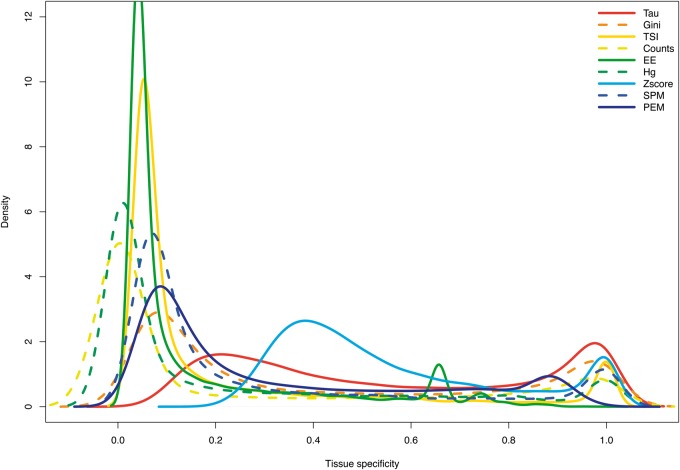
Distribution of tissue-specificity parameters with data for human RNA-seq of 27 tissues. Graph created with density function from R, which computes kernel density estimates. A colour version of this figure is available at BIB online: https://academic.oup.com/bib.

All methods show a bimodal distribution of gene scores: most genes are either broadly expressed or specific, with only few in between. This is true both with RNA-seq data (Figure 1 and Supplementary Figure S2) and with microarray data (Supplementary Figures S3 and S4). Most methods are strongly skewed towards classifying many genes as ubiquitous, and few as tissue specific or intermediate. Z-score has a shifted peak of tissue specificity relative to other metrics. Tau has a less skewed distribution, with the most tissue-specific and intermediate genes, indicating that it might be capturing more of the variance among gene expression patterns.

All methods correlate relatively well with each other (Supplementary Figures S5 and S6), but the relation is often not linear because methods other than Tau and Gini have little variance outside of the most tissue-specific genes. For example, genes which have Tau between 0.85 and 0.95 have Tsi between 0.2 and 0.43 (Supplementary Figure S5).

**Figure 2.**
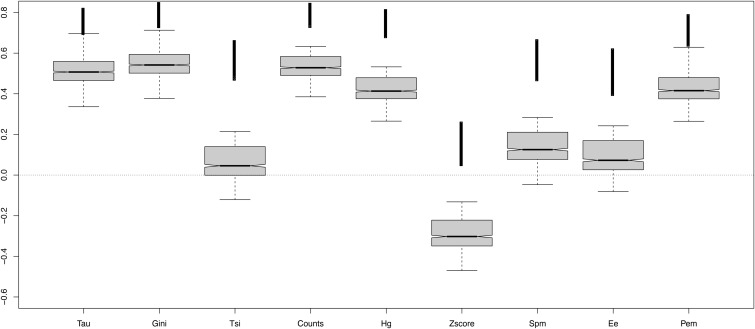
Comparison between tissue-specificity parameters calculated on the same human RNA-seq data set using all 27 tissues versus 1000 random subsets of five tissues.

As a first measure of robustness of tissue-specificity metrics, we compared each metric calculated on the full human RNA-seq data set of 27 tissues, and on subsets of five tissues (Figure 2). Not all permutations were performed, for computational reasons, but a random sample of 1000 permutations. Ideally, the signal for tissue specificity should already be detectable with the five tissues. Tau, Gini, Counts, PEM and the Hg coefficient all show correlations which are not too low (mean *r* > 0.4), indicating that these methods are reasonably robust to the number of tissues. TSI, SPM and EE score show weaker results (mean 0.2 > *r* > 0). The correlation for z-score is even negative, indicating that it should be not used with a small number of tissues, and casting doubt on its utility to robustly estimate tissue specificity. We performed the same analysis in mouse, comparing scores between all 22 available tissues and subsets of five tissues; the results are consistent, but correlations are weaker for all parameters (Supplementary Figure S7). Similarly, we compared the scores using all available tissues (27 in human, 22 in mouse) with the scores using only the 16 tissues shared between these human and mouse data sets; correlations of all parameters are high for human and mouse, and z-score shows again the lowest correlation in all cases (Supplementary Figures S8 and S9).

The choice of tissues to calculate tissue specificity affects the results. All the outliers (stronger correlation) in Figure 2 and Supplementary Figure S7 contain testis tissue. This can be explained by the fact that testis has the largest number of tissue-specific genes (Supplementary Figures S10 and S11). Thus, using a subset which excludes testis produces an estimate of tissue specificity that is biased relative to the full data set, and this bias is only relieved in the few subsets that include testis.

We also analysed robustness of Tau by comparing correlation calculated on all 27 tissues and on all the subsets of 5–26 tissues (Supplementary Figures S12 and S13). Again, all the subsets that are most similar to the full set (outliers with *r* > 0.7) in subsets of five and six tissues contain testis in the set. Conversely, all the subsets that are most different in the full set (outliers with *r* < 0.8) in subsets of 21–26 tissues do not have testis in the subset. There are other outlier subsets that are closer to the main distribution for 25 or 26 tissues: these do include testis, but not brain, which is the second tissue with the most specific genes.

**Figure 3.**
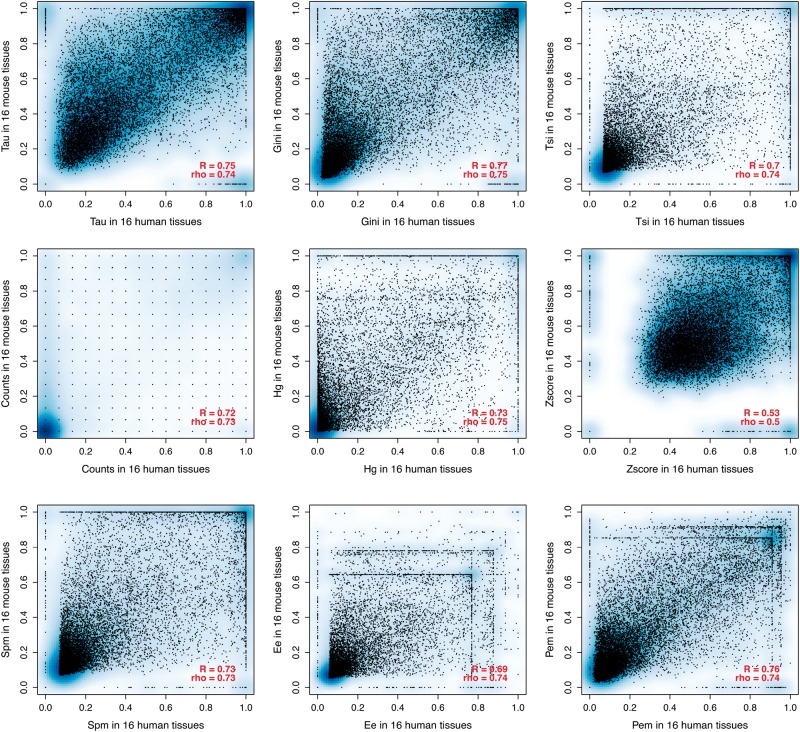
Comparison between tissue-specificity parameters calculated on the 16 common tissues between the human and mouse RNA-seq data sets. All correlations have *P*-value <2.2 × 10^−16^. A colour version of this figure is available at BIB online: https://academic.oup.com/bib.

In addition to being robust to tissue sampling, we expect a good measure of tissue specificity to capture biological signal. A simple expectation of such biological signal is that it should be mostly conserved between orthologues from closely related species such as human and mouse [56]. Thus, we compared the methods in their conservation between human and mouse, using the 16 common tissues (Figure 3). All of the methods, except z-score, show a high correlation (r > 0.69). Specificity parameters calculated on only six common tissues between mouse and human (from the Brawand *et al.* data set) show even higher correlations (*r* > 0.75, Supplementary Figure S14).

**Figure 4.**
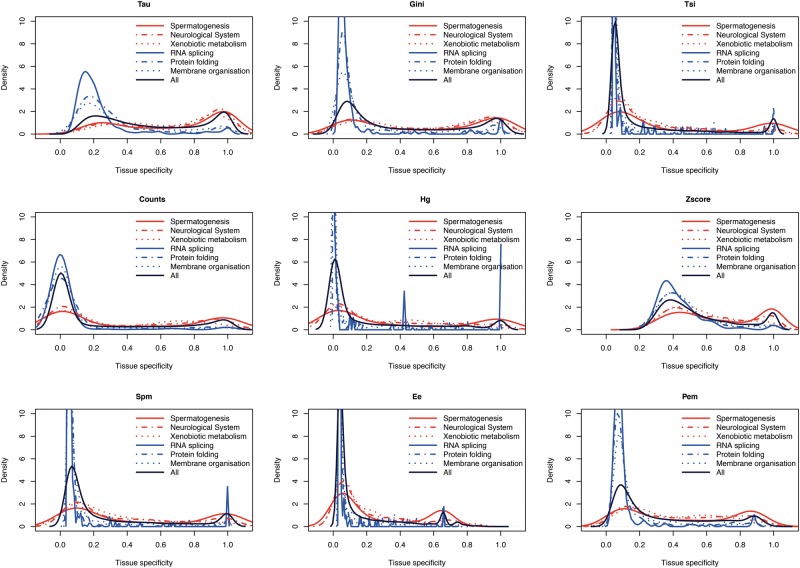
Tissue-specificity parameters of subsets of genes which are expected to be tissue-specific (top three terms, Spermatogenesis to Xenobiotic metabolism lines) or broadly expressed (RNA splicing to Membrane organisation lines), based on associated GO terms (described in Material and Methods). The black line represents the distribution for all genes, including those not associated to any of these GO terms. A colour version of this figure is available at BIB online: https://academic.oup.com/bib.

Another way to capture biological signal is to compare the expression specificity of genes annotated with functions that are expected to be tissue specific, or which are expected to be ubiquitous. For this, we chose three tissue-specific GO terms and three GO terms that are expected to be present in all tissues. The tissue-specific GO terms are spermatogenesis, specific to testis; neurological system process, specific to brain and other neural tissues; and xenobiotic metabolic process, specific to liver and kidney. The broadly expressed GO terms are protein folding, membrane organization and RNA splicing. The distribution of the genes belonging to each category is presented in Figure 4 and Supplementary Figure S15. All of the parameters are successful at recognizing broadly expressed genes (peak of blue lines, as expected, shifted towards 0). But, there are important differences in results for specific genes. Only Tau has a larger peak close to 1 than close to 0. All parameters, except Tau, show strongly bimodal distributions for the genes that are expected to be specific, often with the larger peak at ubiquitous expression. Thus, Tau appears to be more successful at recovering this expected biological signal. We also checked the correlation of genes from tissue-specific functions (according to the three GO terms) between mouse and human orthologues (Supplementary Figure S16). Even if correlations are high and almost the same for all the parameters, the difference is that genes that are expected to be specific are specified by most parameters as ubiquitous. Only Tau reports most of these genes as evolutionarily conserved tissue specific.

**Figure 5.**
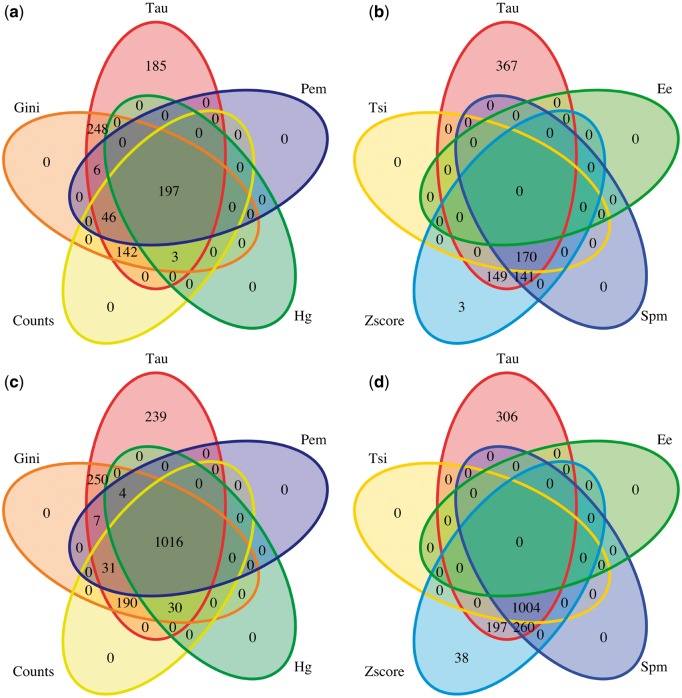
Venn diagram of genes called specific with different parameters, with a cut-off of 0.8 for each parameter; (**A**) and (**B**) genes with their highest expression in the brain; (**C**) and (**D**) genes with their highest expression in the testis; parameters are shown in A/C or B/D for readability, with Tau in common because it calls the most genes tissue specific. A colour version of this figure is available at BIB online: https://academic.oup.com/bib.

Most methods seem to have more difficulty in finding tissue-specificity signal than broad expression signal. We checked whether those tissue-specific genes detected by each method are specific to the method, or also detected by others. Strikingly, almost all tissue-specific genes found by any method are also found by Tau. Gini also reports many tissue-specific genes that are reported by Tau but no other method. This is illustrated with the examples of brain- and testis-specific genes in Figure 5 (for other organs, see Supplementary Figure S17–S41). To call genes specific, a threshold of 0.8 was set, which is after the first peak of the bimodal distribution for most parameters. The same analysis was performed with thresholds of 0.6 and 0.4 (data not shown), and produced similar results: Tau detects all genes that other methods detect plus some that are not detected by any other method. To check whether these additional tissue-specific genes found by Tau are biologically relevant, a GO enrichment test was performed on tissue-specific genes for testis and brain reported by all methods, by Tau alone or only by Tau and Gini (Supplementary Figures S42–S47). Each of these genes sets is indeed enriched in brain- or testis-specific functions, which shows that these were rather false negatives of the other methods than false positives of Tau and Gini.

**Figure 6.**
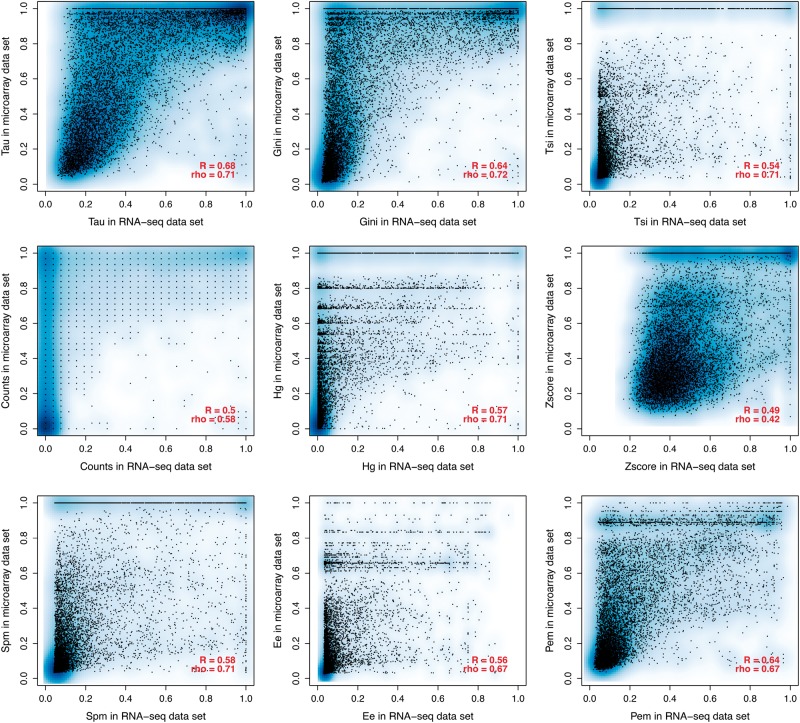
Comparison between tissue-specificity parameters calculated on RNA-seq of 27 tissues versus microarray of 32 tissue in human data sets. All correlations have *P*-value <2.2 × 10^−16^. A colour version of this figure is available at BIB online: https://academic.oup.com/bib.

The same analysis was also performed on the microarray data sets for mouse and human. We compared each metric on a full microarray human data set of 32 tissues and on the subset of five tissues (Supplementary Figure S48). For human, the correlations are weaker than with RNA-seq, even for the best performing metrics: Counts, Gini and Tau (mean 0.2 < *r* < 0.4). For mouse, the correlations on microarray data are better: Counts, Gini and Tau (mean 0.4 < *r* < 0.6) (Supplementary Figure S49). Results for 32 and 14 human tissues, and for 19 and 14 mouse tissues, are shown in Supplementary Figures S50 and S51. The distribution of correlations of Tau calculated on different subsets of tissues is shown in Supplementary Figures S52 and S53. Similarly, in the comparison between human and mouse orthologues, the correlations are much weaker for microarrays than for RNA-seq (Supplementary Figure S54). Specificity values are better correlated between RNA-seq and microarray for the mouse than for the human data sets (Figure 6 and Supplementary Figure S55). This correlation is on the same scale as that between two different RNA-seq data sets, although the correlation is a bit stronger for the RNA-seq data sets (Supplementary Figures S56 and S57). It should be noted that microarray and RNA-seq can only be compared on the subset of genes for which microarray data are usable, which excludes very tissue-specific genes detected only by RNA-seq (Supplementary Figure S58).

Tissue specificity has been reported to be negatively correlated to mean or maximum gene expression level across tissues, i.e. ubiquitous genes have higher expression, and specific genes have lower expression (discussed in [1, 4, 20]). Indeed, we find a negative correlation of all metrics with mean expression; this correlation is similar for RNA-seq (*r* from −0.69 to −0.93) and for microarray (*r* from −0.70 to −0.95) (Supplementary Figures S59–S62). Z-score has the weakest correlation with mean expression on RNA-seq data and on microarray data. The correlation of tissue specificity parameters and maximal expression is also similar with RNA-seq and microarray (Supplementary Figures S63–S66): all the parameters are negatively correlated with maximal expression.

In all the analyses described above, RPKM values were log-transformed, as described in ‘Material and Methods’. In the following, we investigated how stable the results of tissue specificity are if data are not log-normalized or if they are additionally quantile normalized. We compared tissue specificity calculated on log-transformed RPKM (as above), raw RPKM, log-transformed and quantile normalized RPKM (Supplementary Figures S67–S75); quantile normalization was performed across tissues in each data set. In general, quantile normalization has no influence on the results of calculation of tissue specificity (Supplementary Figures S74 and S75). Expectedly, removing log-transformation has a greater influence on all parameters, in the direction of detecting more tissue-specificity, sometimes losing completely the signal of broad expression, e.g. Tau (Supplementary Figure S68). Moreover, in the absence of log-transformation, the correlations between subsets of tissues or between species are in general weaker (Supplementary Figures S69–S70). The normalization has no influence on Counts, as expected, as only yes/no for the expression is taken in the account. Tau, Gini, TSI and Hg show the highest correlations between normalized and non-normalized data (Supplementary Figures S72 and S73), thus appearing more robust.

## Discussion

We analysed nine parameters to calculate tissue specificity. We compared the methods with respect to their stability to the number of tissues, their correlation between one-to-one orthologues in human and mouse, their power in detecting tissue-specific genes and their distribution of values. As many experiments do not have many tissues, it is important that tissue specificity can be calculated reliably on few tissues.

Different methods of calculating tissue specificity take into account different properties of expression. The Counts method does not take in account the amplitude of differences between tissues. This is the simplest method; yet, if the threshold is chosen properly, it gives surprisingly good results. Distribution of Counts tissue specificity depending on the chosen threshold is presented in Supplementary Figure S76: with too high or too low threshold, most genes are reported as not specific, but it is robust to a change of one order of magnitude (1–10 RPKM). Tau and TSI both use the information of expression of a gene in each tissue and its maximal expression over all tissues. The difference between Tau and TSI is that Tau also takes into account the number of tissues. The Hg coefficient is also similar, but differs in that instead of the maximal expression (necessarily in a specific tissue) the sum of expression over tissues is used, and each normalized value is multiplied by log of the value. And for the SPM score, each value (squared) is corrected by the sum of squared gene expression across all tissues. The EE score also corrects each expression value by the sum of gene expression across tissues as well as by the sum of expression in the target tissue. The PEM score is simply the logarithm (base 10) of the EE score. As these coefficients are normalized by either maximal expression of the gene or by the sum of expression of the gene, they are not sensitive to its absolute expression level. Z-score is the only method that takes the standard deviation of expression into account. An overview of the methods with their shared components (e.g. max expression appears in Tau and in TSI) is presented in the Supplementary Materials.

Tau appears consistently to be the most robust method in our analyses. Comparing coefficients calculated on different sized data sets, Tau showed one of the highest correlations (Figure 2 and Supplementary Figures S7–S9). And, while it may be debated what is it the ‘best' distribution between ubiquitous and specific genes, we note that Tau provides well-separated groups with lower skew towards calling most genes ubiquitous or tissue specific than other methods (Figure 1 and Supplementary Figure S2); and it found more tissue-specific genes (Figure 5, Supplementary Figures S10, S11 and S16–S41). Tau also showed a robust behaviour according to normalization of data (Supplementary Figures S72 and S73). With the GO analysis performed, Tau is the best in recognizing tissue-specific genes (Figure 4 and Supplementary Figure S15), and conversely tissue-specific genes found only with Tau have functional annotations that are consistent with their tissue of highest expression (Supplementary Figures S41–44).

When a score per tissue is needed, the PEM score showed acceptable results, except for non-log-transformed mouse RNA-seq (Supplementary Figure S73), and it is most similar to Tau. An association between scores and tissues can be also obtained by simply using Tau and choosing the tissue with the highest expression.

Z-score and PEM score are the only methods to detect under-expression. But z-score is the most sensitive to the number of tissues used for analysis, and generally performs poorly on most tests. The PEM score performs relatively well, though it is skewed to 0, i.e. to calling genes as ubiquitous (Figure 1 and Supplementary Figure S2).

In general, almost twice as many genes can be called expressed in at least one tissue with RNA-seq than with microarray (see ‘Materials and Methods’ and Supplementary Figure S58). It has been reported that the detection of lowly expressed genes is better with RNA-seq than with microarrays [57–59]. Because the most tissue-specific genes are often lowly expressed [1, 4, 20], RNA-seq can detect specific genes that were not detected using microarrays (Supplementary Figure S58). We observe that the correlation between RNA-seq and microarray data set is of the same scale as the correlation between two RNA-seq data sets (Figure 6 and Supplementary Figures S55–S57). It should be noted that the correlation between microarray and RNA-seq is calculated only on half of the genes, mostly excluding specific ones, and that the second RNA-seq data set has only six tissues, which could make the correlation between RNA-seq data sets weaker.

Generally, the tissue specificity estimated from different data types appears to be different. This is notable relative to the number of tissues (Figure 2 compared with Supplementary Figure S48 and Supplementary Figure S7 compared with Supplementary Figure S49): tissue specificity calculated on microarray with a small number of tissues is poorly correlated to that with a larger number in human data, but the opposite is seen for mouse data. The correlation between species is higher for RNA-seq than for microarray (Figure 3 and Supplementary Figure S54). Our observations imply that past results, which relied on microarray data for the evolutionary interpretation of tissue specificity, should be treated with great caution.

With any method of calculating tissue specificity, it should be noted that if the proportion of closely related tissues (e.g. different parts of the brain) in the set of tissues is high, the tissue specificity will be biased. Moreover, usually a large proportion of tissue-specific genes are testis specific, so special care should be taken in comparing data sets with and without testis. Thus, in general, during the analysis of tissue specificity, care should be taken in sampling the tissues used.

For studying the evolution of gene expression, we show here that tissue specificity is a biologically relevant parameter that has strong conservation between relatively closely related species such as human and mouse. Our results show that using a robust method such as Tau allows evolutionary comparisons even when tissue sampling somewhat differs (e.g. correlation with 27 versus 16 tissues). In light of the difficulties of comparing expression levels between species [16, 60, 61], tissue specificity holds promise not only as a confounding factor to take into account in molecular evolution [20], but also as a measure of biological function that can be compared between genes and between species.

Tissue specificity is also important for biomedical applications, as, for example, cancer malignancies can be very tissue specific [62]. More broadly, causative eQTLs identified by genome-wide association study can affect tissue-specific regulation of genes, which is linked to a weak enrichment in disease association of single nucleotide polymorphisms [63].

## Conclusion

The best overall method to measure expression specificity appears to be Tau, which is reassuring, considering the number of studies in which it has been used. Counts is the simplest method, and if the threshold is chosen properly, it shows good results, although with a tendency to under-call tissue-specific genes. Gini is similar to Tau in its performance. These methods allow to capture a signal that has both functional and evolutionary significance to the genes that are studied.

**Key Points**

- Tissue specificity can be measured reliably, and carries relevant biological information.
- Tissue specificity is largely conserved between human–mouse orthologues.
- Tau is the best metric to measure tissue specificity, while Gini and simple Counts also work well.
- RNA-seq is more powerful than microarrays to detect tissue-specific genes.

## Supplementary data

Supplementary data are available online at http://bib.oxfordjournals.org/

## Supplementary Material

Supplementary Data

## Notes

**Nadezda Kryuchkova-Mostacci** is a doctoral student in biology in the Department of Ecology and Evolution, University of Lausanne. Her main interest is in understanding the relation between gene expression patterns and molecular evolution.

## Notes

**Marc Robinson-Rechavi** is an associate professor at the Department of Ecology and Evolution in the University of Lausanne, and Group Leader at the Swiss Institute of Bioinformatics. His main interest is in the evolution of animal genomes in the context of organismal function and development.
