# Supplementary material for: A benchmark of gene expression tissue-specificity metrics
Source: Brief Bioinform. 2016 Feb 18;18(2):205–14. doi: 10.1093/bib/bbw008 (PMC5444245; doi:10.1093/bib/bbw008)
Supplement: Supplementary Data [file bbw008_supp.zip › TScomparison_Supplementary.pdf]

Supplementary Materials

Derivation of all the methods used in the paper

For all equations apply:

$$x_i \text{ is the expression of the gene in tissue } i$$
$$n \text{ is the number of tissues}$$

Derivation of Tau and TSI similarity:

$$\tau = \frac{\sum_{i=1}^{n}(1-\widehat{x_i})}{n-1};\ \widehat{x_i} = \frac{x_i}{\max\limits_{1\le i\le n}(x_i)}\ and\ TSI = \frac{\max\limits_{1\le i\le n}(x_i)}{\sum_{i=1}^{n} x_i} \rightarrow$$

$$\tau = \frac{\sum_{i=1}^{n}\left(1-\frac{x_i}{\max\limits_{1\le i\le n}(x_i)}\right)}{n-1} = \frac{n-\sum_{i=1}^{n}\frac{x_i}{\max\limits_{1\le i\le n}(x_i)}}{n-1} = \frac{n-\frac{\sum_{i=1}^{n}x_i}{\max\limits_{1\le i\le n}(x_i)}}{n-1}$$

$$= \left(n-\frac{\sum_{i=1}^{n}x_i}{\max\limits_{1\le i\le n}(x_i)}\right) * \frac{1}{n-1} = \frac{n}{n-1} - \frac{\sum_{i=1}^{n}x_i}{(n-1) * \max\limits_{1\le i\le n}(x_i)} \rightarrow$$

$$\tau = \frac{n}{n-1} - \frac{1}{(n-1) * TSI}$$

SPM original equation:

$$SPM = \frac{\left|(X_i * X_p)/\|X_p\|\right|}{\|X_p\|} \rightarrow SPM = \frac{x_i^2}{X_p \cdot X_p}$$

$$X_p \text{ vector of the gene expression across all tissues}$$

Derivation of SPM equation:

$$SPM = \frac{\left|\frac{(X_i \cdot X_p)}{\|X_p\|}\right|}{\|X_p\|} \rightarrow$$

$$X_i = (0, \dots, x_i, \dots, 0)\ and\ X_p = (x_1, \dots, x_i, \dots, x_n)$$

$$so\ X_i \cdot X_p = 0 * x_1 + \cdots + x_i * x_i + \cdots + 0 * x_n = x_i^2$$

$$SPM = \frac{\left|\frac{x_i^2}{\|X_p\|}\right|}{\|X_p\|} \rightarrow SPM = \frac{x_i^2}{\|X_p\| * \|X_p\|} \rightarrow$$

$$\|X_p\| = \sqrt{x_1^2 + x_i^2 + x_n^2}\ so\ \|X_p\| * \|X_p\| = X_p \cdot X_p$$

$$SPM = \frac{x_i^2}{X_p \cdot X_p} = \frac{x_i^2}{\sum_{i=1}^{n} x_i^2}$$

Derivation of Gini equation:

$$G = \frac{n+1}{n} - \frac{2\sum_{i=1}^{n}(n+1-i)x_i}{n\sum_{i=1}^{n}x_i} = \frac{n+1}{n} - \frac{2\left(\sum_{i=1}^{n}\left((n+1)x_i - ix_i\right)\right)}{n\sum_{i=1}^{n}x_i}$$

$$= \frac{n+1}{n} - \frac{2(n+1)\sum_{i=1}^{n}x_i - 2\sum_{i=1}^{n}ix_i}{n\sum_{i=1}^{n}x_i} = \frac{n+1}{n} - \frac{2(n+1)}{n} + \frac{2\sum_{i=1}^{n}ix_i}{n\sum_{i=1}^{n}x_i}$$

$$= \frac{2\sum_{i=1}^{n}ix_i}{n\sum_{i=1}^{n}x_i} - \frac{n+1}{n}$$

Hg original equation:

$$H_g = -\sum_{1 \le t \le N} p_{t|g} * \log_2\left(p_{t|g}\right) \; ; \; p_{t|g} = \frac{w_{g,t}}{\sum_{1 \le t \le N} w_{g,t}}$$

$$N\ number\ of\ tissues;\ p_{t|g}\ relative\ expression\ of\ gene\ g\ in\ tissue\ t$$
$$w_{g,t}\ expression\ of\ g\ in\ t; Ranges\ 0\ (single\ tissue) to\ \log_2(N)\ \ (uniformly)$$

Derivation of Hg equation:

$$H_g = -\sum_{i=1}^{n} p_i * \log_2(p_i) \; ; \; p_i = \frac{x_i}{\sum_{i=1}^{n} x_i} \rightarrow$$

$$H_g = -\sum_{i=1}^{n} \frac{x_i}{\sum_{i=1}^{n} x_i} * \log_2 \frac{x_i}{\sum_{i=1}^{n} x_i}$$

EEi original equation:

$$EE_i(g) = \frac{e_i(g)}{f_i(g)};\ f_i = E(g)p_i;\ E(g) = \sum_{i=1}^{n} e_i(g)\ ;\ p_i = \frac{s_i}{\sum_{i=1}^{n} s_i}$$

$$P_i(g) = \sum_{x=e_i(g)}^{E(g)} \binom{E(g)}{x} p_i^x (1-p_i)^{E(g)-x}$$

$$g\ for\ gene\ in\ tissue\ i;\ e_i(g)\ for\ \#\ of\ EST\ in\ tissue\ i;\ f_i\ for\ expected\ \#\ of\ EST$$

$$s_i\ sum\ of\ \#\ of\ EST\ of\ all\ g\ in\ i$$

$$EE_i(g) > 5\ and\ P_i(g) < \ 10^{-3.5}$$

Derivation of EEi equation:

$$EE_i = \frac{x_i}{f_i};\ f_i = Ep_i;\ E = \sum_{i=1}^{n} x_i\ ;\ p_i = \frac{s_i}{\sum_{i=1}^{n} s_i} \rightarrow$$

$$EE_i = \frac{x_i}{\sum_{i=1}^{n} x_i * \frac{s_i}{\sum_{i=1}^{n} s_i}} = \frac{\sum_{i=1}^{n} s_i}{s_i} * \frac{x_i}{\sum_{i=1}^{n} x_i}$$

$$s_i\ summary\ of\ the\ expression\ of\ all\ genes\ in\ tissue\ i$$

Derivation of z score equation:

$$z = \frac{x_i - \mu}{\sigma}$$

$$\mu\ is\ mean;\ \sigma\ is\ standard\ deviation$$

$$\mu = \frac{\sum_{i=1}^{n} x_i}{n}\ and\ \sigma = \sqrt{\frac{\sum_{i=1}^{n}(x_i - \mu)^2}{n-1}} \rightarrow$$

$$z = \frac{x_i - \frac{\sum_{i=1}^{n} x_i}{n}}{\sqrt{\frac{\sum_{i=1}^{n}\left(x_i - \frac{\sum_{i=1}^{n} x_i}{n}\right)^2}{n-1}}} = \left(x_i - \frac{\sum_{i=1}^{n} x_i}{n}\right) * \frac{\sqrt{n-1}}{\sqrt{\sum_{i=1}^{n}\left(x_i - \frac{\sum_{i=1}^{n} x_i}{n}\right)^2}} =$$

$$= \frac{nx_i - \sum_{i=1}^{n} x_i}{n} * \frac{\sqrt{n-1}}{\sqrt{\sum_{i=1}^{n}\left(\frac{nx_i - \sum_{i=1}^{n} x_i}{n}\right)^2}} = \frac{nx_i - \sum_{i=1}^{n} x_i}{n} * \frac{\sqrt{n-1}}{\sqrt{\frac{1}{n^2}\sum_{i=1}^{n}(nx_i - \sum_{i=1}^{n} x_i)^2}} =$$

$$= \frac{\sqrt{n-1} * (nx_i - \sum_{i=1}^{n} x_i)}{\sqrt{\sum_{i=1}^{n}(nx_i - \sum_{i=1}^{n} x_i)^2}}$$

PEM original equation:

$$PEM = \log_{10}\frac{o}{e}; e = G * \frac{N}{T}$$

$$o\ observed\ \#\ of\ EST\ for\ g\ in\ tissue\ i; e\ expected\ \#\ of\ EST;$$

$$G\ total\ \#\ of\ EST\ for\ g;\ N\ total\ \#\ of\ EST\ for\ tissue\ i; T\ total\ \#\ EST$$

$$PEM < 0\ under - expressed, PEM > 0\ over - expressed$$

Derivation of PEM equation:

$$PEM = \log_{10}\frac{x_i}{e}; e = \sum_{i=1}^{n} x_i * \frac{s_i}{\sum_{i=1}^{n} s_i} \rightarrow$$

$$PEM = \log_{10}\frac{x_i}{\sum_{i=1}^{n} x_i * \frac{s_i}{\sum_{i=1}^{n} s_i}} = \log_{10}\left(\frac{\sum_{i=1}^{n} s_i}{s_i} * \frac{x_i}{\sum_{i=1}^{n} x_i}\right) = \log_{10} EE_i$$

$$s_i\ summary\ of\ the\ expression\ of\ all\ genes\ in\ tissue\ i$$

Methods with shared components highlighted

$$\tau = \frac{n}{n-1} - \frac{\sum_{i=1}^{n} x_i}{(n-1) * \max\limits_{1 \le i \le n}(x_i)}$$

$$G = \frac{2\sum_{i=1}^{n} i\, x_i}{n\sum_{i=1}^{n} x_i} - \frac{n+1}{n}$$

$$TSI = \frac{\max\limits_{1 \le i \le n}(x_i)}{\sum_{i=1}^{n} x_i}$$

$$Counts = n_{expressed}$$

$$H_g = -\sum_{i=1}^{n} \frac{x_i}{\sum_{i=1}^{n} x_i} * \log_2 \frac{x_i}{\sum_{i=1}^{n} x_i}$$

$$z = \frac{\sqrt{n-1} * \left(nx_i - \sum_{i=1}^{n} x_i\right)}{\sqrt{\sum_{i=1}^{n}\left(nx_i - \sum_{i=1}^{n} x_i\right)^2}}$$

$$SPM = \frac{x_i^2}{\sum_{i=1}^{n} x_i^2}$$

$$EE_i = \frac{\sum_{i=1}^{n} s_i}{s_i} * \frac{x_i}{\sum_{i=1}^{n} x_i}$$

$$PEM = \log_{10}\left(\frac{\sum_{i=1}^{n} s_i}{s_i} * \frac{x_i}{\sum_{i=1}^{n} x_i}\right)$$

Supplementary figures

All figures are downloadable from Figshare:

http://dx.doi.org/10.6084/m9.figshare.1558257

Supplementary data

Gene list for specific GO terms for human

Neurological system process	Spermatogenesis	Xenobiotic metabolic process
ENSG00000079215	ENSG00000173744	ENSG00000262968
ENSG00000132639	ENSG00000186075	ENSG00000133433
ENSG00000091664	ENSG00000130202	ENSG00000184674
ENSG00000111371	ENSG00000144747	ENSG00000085871
ENSG00000018625	ENSG00000137812	ENSG00000073067
ENSG00000135821	ENSG00000118434	ENSG00000137869
ENSG00000123360	ENSG00000042813	ENSG00000255974
ENSG00000107404	ENSG00000204193	ENSG00000106258
ENSG00000165804	ENSG00000028839	ENSG00000100031
ENSG00000151834	ENSG00000205186	ENSG00000197408
ENSG00000206561	ENSG00000007168	ENSG00000197838
ENSG00000087085	ENSG00000261132	ENSG00000197446
ENSG00000114349	ENSG00000054796	ENSG00000167600
ENSG00000214415	ENSG00000137090	ENSG00000233151
ENSG00000163825	ENSG00000185823	ENSG00000157593
ENSG00000136514	ENSG00000149634	ENSG00000138801
ENSG00000212127	ENSG00000148200	ENSG00000160870
ENSG00000255374	ENSG00000233348	ENSG00000160868
ENSG00000128519	ENSG00000142168	ENSG00000155016
ENSG00000134183	ENSG00000092445	ENSG00000003137
ENSG00000261984	ENSG00000120210	ENSG00000021461
ENSG00000262612	ENSG00000136574	ENSG00000111275
ENSG00000262103	ENSG00000215641	ENSG00000088826
ENSG00000166473	ENSG00000184361	ENSG00000124786
ENSG00000107593	ENSG00000145375	ENSG00000213648
ENSG00000169962	ENSG00000100031	ENSG00000149124
ENSG00000179002	ENSG00000184571	ENSG00000178814
ENSG00000196277	ENSG00000236424	ENSG00000187553
ENSG00000176884	ENSG00000079462	ENSG00000095596
ENSG00000184845	ENSG00000132141	ENSG00000144959
ENSG00000160716	ENSG00000172352	ENSG00000142973
ENSG00000170345	ENSG00000149948	ENSG00000187048
ENSG00000128271	ENSG00000137875	ENSG00000100577
ENSG00000069696	ENSG00000128731	ENSG00000160882
ENSG00000180176	ENSG00000153208	ENSG00000179142
ENSG00000151577	ENSG00000179165	ENSG00000146233
ENSG00000164885	ENSG00000183795	ENSG00000244067
ENSG00000149295	ENSG00000167601	ENSG00000198457
ENSG00000100302	ENSG00000005156	ENSG00000243955
ENSG00000116288	ENSG00000187191	ENSG00000174156
ENSG00000147571	ENSG00000172818	ENSG00000198682
ENSG00000145708	ENSG00000114374	ENSG00000170899
ENSG00000169676	ENSG00000152582	ENSG00000180432
ENSG00000145335	ENSG00000182583	ENSG00000171428
ENSG00000169057	ENSG00000141577	ENSG00000156006
ENSG00000109738	ENSG00000084674	ENSG00000169764
ENSG00000130751	ENSG00000134057	ENSG00000105398
ENSG00000151322	ENSG00000122970	ENSG00000093010
ENSG00000105464	ENSG00000186471	ENSG00000151224
ENSG00000150275	ENSG00000102904	ENSG00000036530
ENSG00000184564	ENSG00000101190	ENSG00000186104
ENSG00000181195	ENSG00000119048	ENSG00000001084
ENSG00000273079	ENSG00000124678	ENSG00000124212
ENSG00000183454	ENSG00000205916	ENSG00000261052
ENSG00000164434	ENSG00000215077	ENSG00000181019
ENSG00000145888	ENSG00000003137	ENSG00000148832
ENSG00000268563	ENSG00000135525	ENSG00000110711
ENSG00000262933	ENSG00000166171	ENSG00000088002
ENSG00000110680	ENSG00000086288	ENSG00000038274
ENSG00000129991	ENSG00000095627	ENSG00000084207
ENSG00000120907	ENSG00000164256	ENSG00000130649
ENSG00000171873	ENSG00000175294	ENSG00000166741
ENSG00000170214	ENSG00000146857	ENSG00000145217
ENSG00000175344	ENSG00000185894	ENSG00000019186
ENSG00000259880	ENSG00000125398	ENSG00000198203
ENSG00000145050	ENSG00000102781	ENSG00000059377
ENSG00000071242	ENSG00000172288	ENSG00000162813
ENSG00000135744	ENSG00000125207	ENSG00000198075
ENSG00000180772	ENSG00000010803	ENSG00000133475
ENSG00000269602	ENSG00000140057	ENSG00000137364

ENSG00000167081	ENSG00000234495	ENSG00000134716
ENSG00000163485	ENSG00000180616	ENSG00000189221
ENSG00000169760	ENSG00000173930	ENSG00000069535
ENSG00000121297	ENSG00000184903	ENSG00000144852
ENSG00000115419	ENSG00000139618	ENSG00000108242
ENSG00000164438	ENSG00000079393	ENSG00000165841
ENSG00000109132	ENSG00000219435	ENSG00000138109
ENSG00000180613	ENSG00000152705	ENSG00000138115
ENSG00000196338	ENSG00000118245	ENSG00000148795
ENSG00000169992	ENSG00000132122	ENSG00000109814
ENSG00000166833	ENSG00000130368	ENSG00000135929
ENSG00000108684	ENSG00000173262	ENSG00000133313
ENSG00000187848	ENSG00000008294	ENSG00000163221
ENSG00000130234	ENSG00000181638	ENSG00000116704
ENSG00000115353	ENSG00000168619	ENSG00000154930
ENSG00000112096	ENSG00000136449	ENSG00000168906
ENSG00000261125	ENSG00000264668	ENSG00000206338
ENSG00000260283	ENSG00000129873	ENSG00000241635
ENSG00000168263	ENSG00000197081	ENSG00000241119
ENSG00000171189	ENSG00000182415	ENSG00000167165
ENSG00000106688	ENSG00000144962	ENSG00000155850
ENSG00000102057	ENSG00000070950	ENSG00000240224
ENSG00000120251	ENSG00000151445	ENSG00000165092
ENSG00000166206	ENSG00000180113	ENSG00000244474
ENSG00000132164	ENSG00000005513	ENSG00000242366
ENSG00000157103	ENSG00000120669	ENSG00000140459
ENSG00000004660	ENSG00000070759	ENSG00000138061
ENSG00000186297	ENSG00000039600	ENSG00000148834
ENSG00000135519	ENSG00000118156	ENSG00000065621
ENSG00000182256	ENSG00000133101	ENSG00000140465
ENSG00000173338	ENSG00000133808	ENSG00000140505
ENSG00000152822	ENSG00000012817	ENSG00000231852
ENSG00000174775	ENSG00000122966	ENSG00000111012
ENSG00000169282	ENSG00000138207	ENSG00000167910
ENSG00000128683	ENSG00000143452	ENSG00000116984
ENSG00000124140	ENSG00000161800	ENSG00000100197
ENSG00000171408	ENSG00000136811	ENSG00000120915
ENSG00000206511	ENSG00000196352	ENSG00000106546
ENSG00000101188	ENSG00000173698	ENSG00000124275
ENSG00000152578	ENSG00000124610	ENSG00000101444
ENSG00000170049	ENSG00000164104	ENSG00000187134
ENSG00000197535	ENSG00000128242	ENSG00000172817
ENSG00000160014	ENSG00000145757	ENSG00000131069
ENSG00000136854	ENSG00000152430	ENSG00000100983
ENSG00000167414	ENSG00000006282	ENSG00000001630
ENSG00000164129	ENSG00000159374	ENSG00000130540
ENSG00000143727	ENSG00000242875	ENSG00000023909
ENSG00000152495	ENSG00000168594	ENSG00000008394
ENSG00000168036	ENSG00000182541	ENSG00000163106
ENSG00000137766	ENSG00000089094	ENSG00000186529
ENSG00000138435	ENSG00000086061	ENSG00000186204
ENSG00000153822	ENSG00000164611	ENSG00000198099
ENSG00000034053	ENSG00000100105	ENSG00000186115
ENSG00000111275	ENSG00000244588	ENSG00000172955
ENSG00000055118	ENSG00000123131	ENSG00000187758
ENSG00000117013	ENSG00000187475	ENSG00000171903
ENSG00000167083	ENSG00000087338	ENSG00000168765
ENSG00000155974	ENSG00000033800	ENSG00000196616
ENSG00000101204	ENSG00000166220	ENSG00000213366
ENSG00000161509	ENSG00000071859	ENSG00000196344
ENSG00000113319	ENSG00000165269	ENSG00000134184
ENSG00000123700	ENSG00000269964	ENSG00000134201
ENSG00000263324	ENSG00000166224	ENSG00000134202
ENSG00000075043	ENSG00000112592	ENSG00000232414
ENSG00000132155	ENSG00000204188	ENSG00000235134
ENSG00000078053	ENSG00000005889	ENSG00000066813
ENSG00000179546	ENSG00000179580	ENSG00000196620
ENSG00000182389	ENSG00000005955	ENSG00000166743
ENSG00000114812	ENSG00000102230	ENSG00000213759
ENSG00000114353	ENSG00000111837	ENSG00000135226
ENSG00000042753	ENSG00000184650	ENSG00000156096
ENSG00000157764	ENSG00000127554	ENSG00000173597
ENSG00000149782	ENSG00000169297	ENSG00000109193
ENSG00000232569	ENSG00000169981	ENSG00000006625
ENSG00000173267	ENSG00000204713	ENSG00000095303
ENSG00000164418	ENSG00000158480	ENSG00000101076
ENSG00000182450	ENSG00000164708	ENSG00000197165

ENSG00000188157	ENSG00000124226	ENSG00000196502
ENSG00000101152	ENSG00000168556	ENSG00000143198
ENSG00000136449	ENSG00000169800	ENSG00000007933
ENSG00000129990	ENSG00000164972	ENSG00000094963
ENSG00000261941	ENSG00000024048	ENSG00000010932
ENSG00000173826	ENSG00000119772	ENSG00000076258
ENSG00000112499	ENSG00000170684	ENSG00000266592
ENSG00000125675	ENSG00000168757	ENSG00000105852
ENSG00000237112	ENSG00000183035	ENSG00000147465
ENSG00000110076	ENSG00000226941	ENSG00000214435
ENSG00000109103	ENSG00000100811	ENSG00000162433
ENSG00000220205	ENSG00000112981	ENSG00000176884
ENSG00000120049	ENSG00000153094	ENSG00000151726
ENSG00000179841	ENSG00000235307	ENSG00000116791
ENSG00000104783	ENSG00000111087	ENSG00000244122
ENSG00000157542	ENSG00000183753	ENSG00000243135
ENSG00000157551	ENSG00000112053	
ENSG00000105737	ENSG00000177463	
ENSG00000182621	ENSG00000188120	
ENSG00000149927	ENSG00000158077	
ENSG00000135643	ENSG00000182985	
ENSG00000136750	ENSG00000220201	
ENSG00000184185	ENSG00000136603	
ENSG00000197584	ENSG00000012061	
ENSG00000171303	ENSG00000160207	
ENSG00000112294	ENSG00000107679	
ENSG00000053918	ENSG00000013297	
ENSG00000073969	ENSG00000233803	
ENSG00000093010	ENSG00000229549	
ENSG00000171121	ENSG00000087088	
ENSG00000183020	ENSG00000228927	
ENSG00000145451	ENSG00000107140	
ENSG00000135423	ENSG00000258992	
ENSG00000162989	ENSG00000166762	
ENSG00000148680	ENSG00000163577	
ENSG00000080709	ENSG00000161533	
ENSG00000138031	ENSG00000173809	
ENSG00000177189	ENSG00000095794	
ENSG00000196586	ENSG00000205944	
ENSG00000006125	ENSG00000188782	
ENSG00000156113	ENSG00000118816	
ENSG00000143473	ENSG00000128886	
ENSG00000145864	ENSG00000229524	
ENSG00000135312	ENSG00000168092	
ENSG00000145863	ENSG00000253797	
ENSG00000158445	ENSG00000159409	
ENSG00000213585	ENSG00000139874	
ENSG00000134769	ENSG00000143450	
ENSG00000022355	ENSG00000142182	
ENSG00000070748	ENSG00000007038	
ENSG00000067715	ENSG00000132749	
ENSG00000182324	ENSG00000004809	
ENSG00000135679	ENSG00000183473	
ENSG00000159248	ENSG00000124733	
ENSG00000113327	ENSG00000106346	
ENSG00000021645	ENSG00000185972	
ENSG00000187714	ENSG00000182134	
ENSG00000168993	ENSG00000197181	
ENSG00000101327	ENSG00000102225	
ENSG00000187486	ENSG00000139734	
ENSG00000127955	ENSG00000196092	
ENSG00000186795	ENSG00000170608	
ENSG00000244165	ENSG00000188425	
ENSG00000165646	ENSG00000013375	
ENSG00000111181	ENSG00000096155	
ENSG00000006071	ENSG00000183395	
ENSG00000072133	ENSG00000154438	
ENSG00000137252	ENSG00000158402	
ENSG00000102468	ENSG00000163421	
ENSG00000149305	ENSG00000204849	
ENSG00000026559	ENSG00000088930	
ENSG00000166736	ENSG00000204848	
ENSG00000082482	ENSG00000180190	
ENSG00000241973	ENSG00000147926	
ENSG00000010379	ENSG00000163923	
ENSG00000124780	ENSG00000234214	
ENSG00000095981	ENSG00000233788	

ENSG00000105971	ENSG00000092345	
ENSG00000115665	ENSG00000165643	
ENSG00000237051	ENSG00000131759	
ENSG00000129159	ENSG00000185775	
ENSG00000164082	ENSG00000227761	
ENSG00000145349	ENSG00000100206	
ENSG00000108797	ENSG00000234734	
ENSG00000204681	ENSG00000185122	
ENSG00000101557	ENSG00000071539	
ENSG00000166073	ENSG00000102387	
ENSG00000198722	ENSG00000129654	
ENSG00000141503	ENSG00000134874	
ENSG00000124493	ENSG00000060138	
ENSG00000189221	ENSG00000165059	
ENSG00000058404	ENSG00000228760	
ENSG00000164326	ENSG00000164871	
ENSG00000099365	ENSG00000229006	
ENSG00000147432	ENSG00000143578	
ENSG00000147434	ENSG00000113916	
ENSG00000006283	ENSG00000234507	
ENSG00000100030	ENSG00000205213	
ENSG00000166862	ENSG00000136709	
ENSG00000140992	ENSG00000135454	
ENSG00000104848	ENSG00000131068	
ENSG00000152214	ENSG00000168454	
ENSG00000078369	ENSG00000131808	
ENSG00000108556	ENSG00000007174	
ENSG00000187730	ENSG00000042980	
ENSG00000137841	ENSG00000057468	
ENSG00000135902	ENSG00000105549	
ENSG00000136099	ENSG00000135951	
ENSG00000196811	ENSG00000237071	
ENSG00000263156	ENSG00000171552	
ENSG00000166006	ENSG00000116918	
ENSG00000100433	ENSG00000197386	
ENSG00000117676	ENSG00000196470	
ENSG00000198822	ENSG00000108384	
ENSG00000008056	ENSG00000164736	
ENSG00000156486	ENSG00000070404	
ENSG00000128310	ENSG00000155087	
ENSG00000123454	ENSG00000188486	
ENSG00000161203	ENSG00000170469	
ENSG00000182132	ENSG00000167098	
ENSG00000183395	ENSG00000214929	
ENSG00000145936	ENSG00000153046	
ENSG00000164270	ENSG00000171403	
ENSG00000162188	ENSG00000047936	
ENSG00000100151	ENSG00000169083	
ENSG00000170579	ENSG00000118432	
ENSG00000182687	ENSG00000115548	
ENSG00000143553	ENSG00000167468	
ENSG00000174343	ENSG00000234704	
ENSG00000168135	ENSG00000108423	
ENSG00000104888	ENSG00000096717	
ENSG00000197971	ENSG00000101412	
ENSG00000105711	ENSG00000178279	
ENSG00000143933	ENSG00000178257	
ENSG00000155980	ENSG00000122304	
ENSG00000149575	ENSG00000175646	
ENSG00000172380	ENSG00000165496	
ENSG00000165300	ENSG00000148634	
ENSG00000165970	ENSG00000177992	
ENSG00000183960	ENSG00000179218	
ENSG00000162104	ENSG00000025156	
ENSG00000138622	ENSG00000137948	
ENSG00000184408	ENSG00000129473	
ENSG00000118260	ENSG00000260508	
ENSG00000172354	ENSG00000258643	
ENSG00000157005	ENSG00000100290	
ENSG00000126458	ENSG00000130005	
ENSG00000178342	ENSG00000071626	
ENSG00000164588	ENSG00000162526	
ENSG00000107282	ENSG00000163935	
ENSG00000206466	ENSG00000152670	
ENSG00000069424	ENSG00000187556	
ENSG00000182255	ENSG00000144451	
ENSG00000183729	ENSG00000108666	

ENSG00000082556	ENSG00000198021	
ENSG00000196961	ENSG00000203926	
ENSG00000070018	ENSG00000064199	
ENSG00000142319	ENSG00000157404	
ENSG00000141933	ENSG00000133962	
ENSG00000104490	ENSG00000142920	
ENSG00000141404	ENSG00000061656	
ENSG00000102466	ENSG00000170275	
ENSG00000099822	ENSG00000105011	
ENSG00000131089	ENSG00000134686	
ENSG00000179097	ENSG00000136943	
ENSG00000102924	ENSG00000234651	
ENSG00000070808	ENSG00000099338	
ENSG00000135750	ENSG00000196116	
ENSG00000121281	ENSG00000133863	
ENSG00000163285	ENSG00000179168	
ENSG00000168243	ENSG00000156414	
ENSG00000168830	ENSG00000103811	
ENSG00000148408	ENSG00000155833	
ENSG00000109158	ENSG00000114547	
ENSG00000142875	ENSG00000087116	
ENSG00000163288	ENSG00000127530	
ENSG00000174021	ENSG00000119318	
ENSG00000183044	ENSG00000128602	
ENSG00000162975	ENSG00000128040	
ENSG00000152315	ENSG00000159625	
ENSG00000171517	ENSG00000176903	
ENSG00000143603	ENSG00000204256	
ENSG00000184611	ENSG00000101079	
ENSG00000077522	ENSG00000073146	
ENSG00000137575	ENSG00000076242	
ENSG00000116032	ENSG00000177947	
ENSG00000131398	ENSG00000198252	
ENSG00000121764	ENSG00000100918	
ENSG00000074317	ENSG00000138039	
ENSG00000198668	ENSG00000147400	
ENSG00000049247	ENSG00000253293	
ENSG00000173175	ENSG00000169340	
ENSG00000120903	ENSG00000184916	
ENSG00000078295	ENSG00000176371	
ENSG00000109099	ENSG00000182035	
ENSG00000151067	ENSG00000100241	
ENSG00000110436	ENSG00000204084	
ENSG00000146276	ENSG00000269058	
ENSG00000232632	ENSG00000262576	
ENSG00000111886	ENSG00000170820	
ENSG00000123560	ENSG00000006047	
ENSG00000103546	ENSG00000214114	
ENSG00000164742	ENSG00000139908	
ENSG00000173805	ENSG00000217442	
ENSG00000149403	ENSG00000122574	
ENSG00000127914	ENSG00000166118	
ENSG00000148660	ENSG00000166086	
ENSG00000168418	ENSG00000145309	
ENSG00000154229	ENSG00000175792	
ENSG00000075461	ENSG00000185825	
ENSG00000079841	ENSG00000078549	
ENSG00000109971	ENSG00000181323	
ENSG00000164794	ENSG00000077800	
ENSG00000168081	ENSG00000091542	
ENSG00000141837	ENSG00000174898	
ENSG00000135355	ENSG00000134627	
ENSG00000082458	ENSG00000131773	
ENSG00000241563	ENSG00000198765	
ENSG00000155511	ENSG00000101443	
ENSG00000185760	ENSG00000121989	
ENSG00000134294	ENSG00000114487	
ENSG00000099337	ENSG00000248099	
ENSG00000132535	ENSG00000130377	
ENSG00000254550	ENSG00000023445	
ENSG00000169684	ENSG00000122557	
ENSG00000080644	ENSG00000075151	
ENSG00000143630	ENSG00000181929	
ENSG00000170745	ENSG00000172139	
ENSG00000117971	ENSG00000204463	
ENSG00000123159	ENSG00000188488	
ENSG00000173786	ENSG00000173531	

ENSG00000136928	ENSG00000197889	
ENSG00000100884	ENSG00000132681	
ENSG00000176533	ENSG00000158796	
ENSG00000171126	ENSG00000143194	
ENSG00000058335	ENSG00000143199	
ENSG00000113262	ENSG00000116741	
ENSG00000122585	ENSG00000066279	
ENSG00000088367	ENSG00000262532	
ENSG00000105143	ENSG00000269063	
ENSG00000126583	ENSG00000268239	
ENSG00000186469	ENSG00000270633	
ENSG00000114279	ENSG00000272358	
ENSG00000127928	ENSG00000268512	
ENSG00000066427	ENSG00000267977	
ENSG00000089558	ENSG00000269645	
ENSG00000142408	ENSG00000129214	
ENSG00000073150	ENSG00000261631	
ENSG00000242616	ENSG00000165209	
ENSG00000161610	ENSG00000164113	
ENSG00000065135	ENSG00000182077	
ENSG00000150672	ENSG00000104044	
ENSG00000182674	ENSG00000132972	
ENSG00000011677	ENSG00000100814	
ENSG00000151704	ENSG00000125779	
ENSG00000120457	ENSG00000236759	
ENSG00000163873	ENSG00000206203	
ENSG00000151079	ENSG00000181552	
ENSG00000111262	ENSG00000112530	
ENSG00000130037	ENSG00000112531	
ENSG00000011132	ENSG00000126803	
ENSG00000165917	ENSG00000112130	
ENSG00000170296	ENSG00000212122	
ENSG00000152413	ENSG00000072364	
ENSG00000116396	ENSG00000125863	
ENSG00000143105	ENSG00000232045	
ENSG00000121361	ENSG00000141497	
ENSG00000177301	ENSG00000204371	
ENSG00000177272	ENSG00000156475	
ENSG00000069431	ENSG00000221829	
ENSG00000112852	ENSG00000102531	
ENSG00000101438	ENSG00000134709	
ENSG00000113205	ENSG00000177045	
ENSG00000081818	ENSG00000179059	
ENSG00000113209	ENSG00000140463	
ENSG00000113211	ENSG00000206376	
ENSG00000196963	ENSG00000108387	
ENSG00000147246	ENSG00000143552	
ENSG00000120324	ENSG00000105679	
ENSG00000197479	ENSG00000167077	
ENSG00000187372	ENSG00000118046	
ENSG00000120327	ENSG00000227333	
ENSG00000155897	ENSG00000227234	
ENSG00000150394	ENSG00000235604	
ENSG00000135569	ENSG00000177990	
ENSG00000171385	ENSG00000161594	
ENSG00000184156	ENSG00000177938	
ENSG00000168959	ENSG00000109832	
ENSG00000139190	ENSG00000165490	
ENSG00000109991	ENSG00000238134	
ENSG00000179915	ENSG00000179598	
ENSG00000134640	ENSG00000149187	
ENSG00000006128	ENSG00000166037	
ENSG00000139574	ENSG00000139549	
ENSG00000129467	ENSG00000162782	
ENSG00000124134	ENSG00000077327	
ENSG00000110218	ENSG00000269853	
ENSG00000174233	ENSG00000262672	
ENSG00000007168	ENSG00000107954	
ENSG00000166501	ENSG00000141933	
ENSG00000006116	ENSG00000125124	
ENSG00000167535	ENSG00000047932	
ENSG00000106236	ENSG00000142025	
ENSG00000169427	ENSG00000187166	
ENSG00000158748	ENSG00000178093	
ENSG00000105642	ENSG00000096063	
ENSG00000106089	ENSG00000146047	
ENSG00000105649	ENSG00000139351	

ENSG00000139910<br>ENSG00000008282<br>ENSG00000171246<br>ENSG00000111664<br>ENSG00000164061<br>ENSG00000177807<br>ENSG00000162728<br>ENSG00000140015<br>ENSG00000158887<br>ENSG00000075711<br>ENSG00000198216<br>ENSG00000165995<br>ENSG00000182963<br>ENSG00000143858<br>ENSG00000263024<br>ENSG00000268229<br>ENSG00000267829<br>ENSG00000260225<br>ENSG00000272633<br>ENSG00000268060<br>ENSG00000262565<br>ENSG00000273357<br>ENSG00000273310<br>ENSG00000268195<br>ENSG00000261309<br>ENSG00000270478<br>ENSG00000260458<br>ENSG00000272706<br>ENSG00000101180<br>ENSG00000131238<br>ENSG00000160469<br>ENSG00000007516<br>ENSG00000154764<br>ENSG00000101298<br>ENSG00000185666<br>ENSG00000111052<br>ENSG00000132872<br>ENSG00000104863<br>ENSG00000148943<br>ENSG00000119396<br>ENSG00000128872<br>ENSG00000171862<br>ENSG00000198010<br>ENSG00000054523<br>ENSG00000114200<br>ENSG00000129749<br>ENSG00000130203<br>ENSG00000181019<br>ENSG00000166405<br>ENSG00000170175<br>ENSG00000244405<br>ENSG00000168539<br>ENSG00000179388<br>ENSG00000101306<br>ENSG00000185652<br>ENSG00000112038<br>ENSG00000125510<br>ENSG00000213199<br>ENSG00000110148<br>ENSG00000169006<br>ENSG00000137474<br>ENSG00000187688<br>ENSG00000185313<br>ENSG00000121743<br>ENSG00000100053<br>ENSG00000244752<br>ENSG00000108821<br>ENSG00000134987<br>ENSG00000170264<br>ENSG00000101850<br>ENSG00000179270<br>ENSG00000164175<br>ENSG00000122375<br>ENSG00000100122<br>ENSG00000121207<br>ENSG00000196431<br>ENSG00000170289	ENSG00000068878<br>ENSG00000244405<br>ENSG00000109906<br>ENSG00000154040<br>ENSG00000001626<br>ENSG00000115257<br>ENSG00000072062<br>ENSG00000225697<br>ENSG00000091138<br>ENSG00000091140<br>ENSG00000086015<br>ENSG00000144468<br>ENSG00000144061<br>ENSG00000134595	

ENSG00000102001<br>ENSG00000102076<br>ENSG00000147380<br>ENSG00000166160<br>ENSG00000205981<br>ENSG00000132518<br>ENSG00000163635<br>ENSG00000116035<br>ENSG00000065154<br>ENSG00000074621<br>ENSG00000099204<br>ENSG00000174483<br>ENSG00000179941<br>ENSG00000164007<br>ENSG00000106536<br>ENSG00000134160<br>ENSG00000163795<br>ENSG00000102104<br>ENSG00000095777<br>ENSG00000163914<br>ENSG00000180245<br>ENSG00000155886<br>ENSG00000186765<br>ENSG00000114124<br>ENSG00000095464<br>ENSG00000170819<br>ENSG00000112041<br>ENSG00000188888<br>ENSG00000138083<br>ENSG00000105392<br>ENSG00000120708<br>ENSG00000263243<br>ENSG00000185527<br>ENSG00000062038<br>ENSG00000135517<br>ENSG00000125863<br>ENSG00000112333<br>ENSG00000124818<br>ENSG00000133256<br>ENSG00000112706<br>ENSG00000198570<br>ENSG00000156194<br>ENSG00000128973<br>ENSG00000048545<br>ENSG00000112599<br>ENSG00000134438<br>ENSG00000175544<br>ENSG00000116016<br>ENSG00000141101<br>ENSG00000080511<br>ENSG00000107736<br>ENSG00000139330<br>ENSG00000158158<br>ENSG00000139329<br>ENSG00000091010<br>ENSG00000108255<br>ENSG00000050555<br>ENSG00000100234<br>ENSG00000156313<br>ENSG00000042781<br>ENSG00000112619<br>ENSG00000135437<br>ENSG00000156973<br>ENSG00000104804<br>ENSG00000160202<br>ENSG00000167995<br>ENSG00000188419<br>ENSG00000188937<br>ENSG00000148604<br>ENSG00000184292<br>ENSG00000125741<br>ENSG00000124479<br>ENSG00000125746<br>ENSG00000066933<br>ENSG00000102218<br>ENSG00000149489<br>ENSG00000092200		

ENSG00000164199<br>ENSG00000145476<br>ENSG00000109047<br>ENSG00000042317<br>ENSG00000139988<br>ENSG00000102805<br>ENSG00000182372<br>ENSG00000140463<br>ENSG00000100987<br>ENSG00000144191<br>ENSG00000116745<br>ENSG00000138686<br>ENSG00000182871<br>ENSG00000130561<br>ENSG00000111215<br>ENSG00000116791<br>ENSG00000158161<br>ENSG00000107618<br>ENSG00000118231<br>ENSG00000163254<br>ENSG00000182187<br>ENSG00000168582<br>ENSG00000132915<br>ENSG00000137955<br>ENSG00000043355<br>ENSG00000131480<br>ENSG00000007372<br>ENSG00000187242<br>ENSG00000198963<br>ENSG00000138061<br>ENSG00000188107<br>ENSG00000104237<br>ENSG00000214140<br>ENSG00000113966<br>ENSG00000129221<br>ENSG00000108370<br>ENSG00000198515<br>ENSG00000179603<br>ENSG00000203668<br>ENSG00000185974<br>ENSG00000176842<br>ENSG00000109501<br>ENSG00000139053<br>ENSG00000130045<br>ENSG00000120500<br>ENSG00000125124<br>ENSG00000163646<br>ENSG00000198691<br>ENSG00000128617<br>ENSG00000091262<br>ENSG00000060718<br>ENSG00000129535<br>ENSG00000101890<br>ENSG00000163093<br>ENSG00000162496<br>ENSG00000140859<br>ENSG00000070729<br>ENSG00000174238<br>ENSG00000081148<br>ENSG00000121039<br>ENSG00000173976<br>ENSG00000263247<br>ENSG00000263040<br>ENSG00000071909<br>ENSG00000120057<br>ENSG00000139219<br>ENSG00000119614<br>ENSG00000198836<br>ENSG00000077498<br>ENSG00000107521<br>ENSG00000112319<br>ENSG00000100949<br>ENSG00000140522<br>ENSG00000136940<br>ENSG00000138472<br>ENSG00000122507<br>ENSG00000172037		

ENSG00000075891<br>ENSG00000121634<br>ENSG00000184302<br>ENSG00000115380<br>ENSG00000143333<br>ENSG00000143341<br>ENSG00000262180<br>ENSG00000116703<br>ENSG00000261910<br>ENSG00000266662<br>ENSG00000265203<br>ENSG00000269180<br>ENSG00000264499<br>ENSG00000271091<br>ENSG00000269057<br>ENSG00000271772<br>ENSG00000260825<br>ENSG00000268221<br>ENSG00000269133<br>ENSG00000269433<br>ENSG00000165474<br>ENSG00000121742<br>ENSG00000151615<br>ENSG00000142168<br>ENSG00000235708<br>ENSG00000131504<br>ENSG00000077782<br>ENSG00000164904<br>ENSG00000100473<br>ENSG00000101849<br>ENSG00000103126<br>ENSG00000197121<br>ENSG00000227801<br>ENSG00000169031<br>ENSG00000166866<br>ENSG00000206290<br>ENSG00000181449<br>ENSG00000116039<br>ENSG00000176402<br>ENSG00000112782<br>ENSG00000180509<br>ENSG00000160460<br>ENSG00000137203<br>ENSG00000139767<br>ENSG00000115155<br>ENSG00000118162<br>ENSG00000182040<br>ENSG00000171885<br>ENSG00000152939<br>ENSG00000230930<br>ENSG00000184058<br>ENSG00000172809<br>ENSG00000112320<br>ENSG00000105929<br>ENSG00000150779<br>ENSG00000039068<br>ENSG00000101276<br>ENSG00000213892<br>ENSG00000164190<br>ENSG00000197753<br>ENSG00000160183<br>ENSG00000125879<br>ENSG00000196767<br>ENSG00000166963<br>ENSG00000164305<br>ENSG00000006611<br>ENSG00000157087<br>ENSG00000188162<br>ENSG00000204311<br>ENSG00000125492<br>ENSG00000166402<br>ENSG00000129355<br>ENSG00000175894<br>ENSG00000167210<br>ENSG00000213614<br>ENSG00000136158<br>ENSG00000019549		

ENSG00000263043<br>ENSG00000181585<br>ENSG00000184154<br>ENSG00000112837<br>ENSG00000196876<br>ENSG00000151090<br>ENSG00000176058<br>ENSG00000111276<br>ENSG00000049860<br>ENSG00000215203<br>ENSG00000187017<br>ENSG00000138081<br>ENSG00000171316<br>ENSG00000109927<br>ENSG00000260234<br>ENSG00000135903<br>ENSG00000178602<br>ENSG00000071127<br>ENSG00000188910<br>ENSG00000157388<br>ENSG00000099800<br>ENSG00000169131<br>ENSG00000105928<br>ENSG00000104313<br>ENSG00000147684<br>ENSG00000105991<br>ENSG00000204248<br>ENSG00000179520<br>ENSG00000095397<br>ENSG00000174804<br>ENSG00000103316<br>ENSG00000155719<br>ENSG00000170615<br>ENSG00000134809<br>ENSG00000091536<br>ENSG00000149972<br>ENSG00000100575<br>ENSG00000233276<br>ENSG00000091137<br>ENSG00000126778<br>ENSG00000271809<br>ENSG00000272728<br>ENSG00000087460<br>ENSG00000197405<br>ENSG00000122136<br>ENSG00000154165<br>ENSG00000221882<br>ENSG00000127780<br>ENSG00000196184<br>ENSG00000154654<br>ENSG00000170340<br>ENSG00000261059<br>ENSG00000167332<br>ENSG00000171944<br>ENSG00000182070<br>ENSG00000176742<br>ENSG00000183251<br>ENSG00000184881<br>ENSG00000242180<br>ENSG00000176239<br>ENSG00000184698<br>ENSG00000184321<br>ENSG00000167359<br>ENSG00000187918<br>ENSG00000181609<br>ENSG00000205329<br>ENSG00000179695<br>ENSG00000132259<br>ENSG00000184933<br>ENSG00000166363<br>ENSG00000166368<br>ENSG00000170683<br>ENSG00000065989<br>ENSG00000171102<br>ENSG00000122718<br>ENSG00000141194<br>ENSG00000261949		

ENSG00000165533		
ENSG00000039987		
ENSG00000196578		
ENSG00000221888		
ENSG00000171180		
ENSG00000197125		
ENSG00000189433		
ENSG00000188269		
ENSG00000183862		
ENSG00000144357		
ENSG00000173610		
ENSG00000271271		
ENSG00000270386		
ENSG00000149133		
ENSG00000140090		
ENSG00000165202		
ENSG00000154678		
ENSG00000262628		
ENSG00000184166		
ENSG00000172150		
ENSG00000172146		
ENSG00000180016		
ENSG00000180340		
ENSG00000163794		
ENSG00000146469		
ENSG00000171867		
ENSG00000068305		
ENSG00000161642		
ENSG00000104327		
ENSG00000138443		
ENSG00000188191		
ENSG00000081189		
ENSG00000110911		
ENSG00000126351		
ENSG00000185900		
ENSG00000105220		
ENSG00000143801		
ENSG00000120738		
ENSG00000176697		
ENSG00000162624		
ENSG00000106278		
ENSG00000197386		
ENSG00000160307		
ENSG00000122877		
ENSG00000118432		
ENSG00000148082		
ENSG00000196743		
ENSG00000159461		
ENSG00000080815		
ENSG00000155966		
ENSG00000146648		
ENSG00000188763		
ENSG00000198400		
ENSG00000263254		
ENSG00000269754		
ENSG00000159082		
ENSG00000146938		
ENSG00000175592		
ENSG00000133216		
ENSG00000184144		
ENSG00000198576		
ENSG00000169862		
ENSG00000105894		
ENSG00000174469		
ENSG00000138138		
ENSG00000177606		
ENSG00000162105		
ENSG00000010810		
ENSG00000137868		
ENSG00000107758		
ENSG00000148053		
ENSG00000115526		
ENSG00000129682		
ENSG00000169220		
ENSG00000154118		
ENSG00000092051		
ENSG00000094914		

ENSG00000251322<br>ENSG00000005249<br>ENSG00000073756<br>ENSG00000147852<br>ENSG00000101405<br>ENSG00000196639<br>ENSG00000136689<br>ENSG00000043591<br>ENSG00000159224<br>ENSG00000120088<br>ENSG00000161638<br>ENSG00000167244<br>ENSG00000180914<br>ENSG00000105409<br>ENSG00000110881<br>ENSG00000096433<br>ENSG00000168496<br>ENSG00000005884<br>ENSG00000166342<br>ENSG00000184381<br>ENSG00000124588<br>ENSG00000108576<br>ENSG00000257793<br>ENSG00000113749<br>ENSG00000129455<br>ENSG00000157045<br>ENSG00000145632<br>ENSG00000030304<br>ENSG00000134259<br>ENSG00000077943<br>ENSG00000272584<br>ENSG00000110492<br>ENSG00000132386<br>ENSG00000107864<br>ENSG00000125872<br>ENSG00000183826<br>ENSG00000198961<br>ENSG00000225950<br>ENSG00000067606<br>ENSG00000112658<br>ENSG00000161681<br>ENSG00000108443<br>ENSG00000118515<br>ENSG00000189056<br>ENSG00000040531<br>ENSG00000159388<br>ENSG00000171532<br>ENSG00000188603<br>ENSG00000116147<br>ENSG00000105227<br>ENSG00000112033<br>ENSG00000112531<br>ENSG00000013297<br>ENSG00000185668<br>ENSG00000142192<br>ENSG00000214285<br>ENSG00000070031<br>ENSG00000131771<br>ENSG00000104755<br>ENSG00000171956<br>ENSG00000124788<br>ENSG00000197283<br>ENSG00000166313<br>ENSG00000115183<br>ENSG00000227460<br>ENSG00000196712<br>ENSG00000113161<br>ENSG00000157404<br>ENSG00000177963<br>ENSG00000213281<br>ENSG00000133703<br>ENSG00000100644<br>ENSG00000100625<br>ENSG00000104936<br>ENSG00000132437<br>ENSG00000171045<br>ENSG00000089041		

ENSG00000186472<br>ENSG00000117758<br>ENSG00000145920<br>ENSG00000213578<br>ENSG00000100505<br>ENSG00000186111<br>ENSG00000079950<br>ENSG00000184702<br>ENSG00000180900<br>ENSG00000260968<br>ENSG00000104164<br>ENSG00000130477<br>ENSG00000163618<br>ENSG00000081803<br>ENSG00000102003<br>ENSG00000102383<br>ENSG00000268353<br>ENSG00000102189<br>ENSG00000064218<br>ENSG00000163531<br>ENSG00000184984<br>ENSG00000170989<br>ENSG00000166086<br>ENSG00000091129<br>ENSG00000188037<br>ENSG00000119782<br>ENSG00000144285<br>ENSG00000164850<br>ENSG00000108405<br>ENSG00000135124<br>ENSG00000100346<br>ENSG00000130226<br>ENSG00000151150<br>ENSG00000169432<br>ENSG00000181656<br>ENSG00000164900<br>ENSG00000165060<br>ENSG00000182636<br>ENSG00000151617<br>ENSG00000115041<br>ENSG00000164128<br>ENSG00000100739<br>ENSG00000179148<br>ENSG00000184117<br>ENSG00000188822<br>ENSG00000128645<br>ENSG00000113302<br>ENSG00000102882<br>ENSG00000120068<br>ENSG00000154153<br>ENSG00000078401<br>ENSG00000141433<br>ENSG00000183695<br>ENSG00000163421<br>ENSG00000136160<br>ENSG00000154277<br>ENSG00000166257<br>ENSG00000169860<br>ENSG00000109255<br>ENSG00000196549<br>ENSG00000121351<br>ENSG00000258839<br>ENSG00000172201<br>ENSG00000148826<br>ENSG00000124920<br>ENSG00000068078<br>ENSG00000197921<br>ENSG00000108381<br>ENSG00000166333<br>ENSG00000157933<br>ENSG00000169247<br>ENSG00000104728<br>ENSG00000153902<br>ENSG00000008277<br>ENSG00000184486<br>ENSG00000196569<br>ENSG00000173402		

ENSG00000145362<br>ENSG00000174607<br>ENSG00000082397<br>ENSG00000116906<br>ENSG00000010278<br>ENSG00000103089<br>ENSG00000105221<br>ENSG00000072415<br>ENSG00000142208<br>ENSG00000104419<br>ENSG00000112367<br>ENSG00000115020<br>ENSG00000063660<br>ENSG00000087053<br>ENSG00000137462<br>ENSG00000104884<br>ENSG00000149256<br>ENSG00000184454<br>ENSG00000100697<br>ENSG00000075073<br>ENSG00000174871<br>ENSG00000105402<br>ENSG00000104368<br>ENSG00000115902<br>ENSG00000003393<br>ENSG00000143786<br>ENSG00000125814<br>ENSG00000152208<br>ENSG00000137880<br>ENSG00000135049<br>ENSG00000077616<br>ENSG00000175785<br>ENSG00000089250<br>ENSG00000134780<br>ENSG00000164535<br>ENSG00000171759<br>ENSG00000143149<br>ENSG00000128573<br>ENSG00000088812<br>ENSG00000205927<br>ENSG00000105329<br>ENSG00000133812<br>ENSG00000107186<br>ENSG00000115211<br>ENSG00000166886<br>ENSG00000141736<br>ENSG00000184113<br>ENSG00000128242<br>ENSG00000172466<br>ENSG00000145191<br>ENSG00000138386<br>ENSG00000165699<br>ENSG00000141385<br>ENSG00000136531<br>ENSG00000167755<br>ENSG00000103740<br>ENSG00000172005<br>ENSG00000102934<br>ENSG00000198121<br>ENSG00000181754<br>ENSG00000164300<br>ENSG00000119718<br>ENSG00000139549<br>ENSG00000121966<br>ENSG00000198947<br>ENSG00000157168<br>ENSG00000008735<br>ENSG00000112425<br>ENSG00000119522<br>ENSG00000205726<br>ENSG00000107295<br>ENSG00000260502<br>ENSG00000124507<br>ENSG00000160325<br>ENSG00000079482<br>ENSG00000127022<br>ENSG00000066468		

ENSG00000079805<br>ENSG00000092964<br>ENSG00000188428<br>ENSG00000185009<br>ENSG00000047579<br>ENSG00000070718<br>ENSG00000177879<br>ENSG00000189114<br>ENSG00000267545<br>ENSG00000135441<br>ENSG00000186222<br>ENSG00000065000<br>ENSG00000132842<br>ENSG00000103723<br>ENSG00000157823<br>ENSG00000196072<br>ENSG00000176406<br>ENSG00000272636<br>ENSG00000272670<br>ENSG00000069869<br>ENSG00000165985<br>ENSG00000197548<br>ENSG00000157796<br>ENSG00000235608<br>ENSG00000132932<br>ENSG00000125878<br>ENSG00000131979<br>ENSG00000171298<br>ENSG00000103657<br>ENSG00000188620<br>ENSG00000174498<br>ENSG00000104320<br>ENSG00000184254<br>ENSG00000133026<br>ENSG00000090861<br>ENSG00000166340<br>ENSG00000100320<br>ENSG00000204120<br>ENSG00000186716<br>ENSG00000169750<br>ENSG00000179772<br>ENSG00000078328<br>ENSG00000159842<br>ENSG00000119508<br>ENSG00000149547<br>ENSG00000084234<br>ENSG00000134121<br>ENSG00000104723<br>ENSG00000158352<br>ENSG00000102158<br>ENSG00000165186<br>ENSG00000268668<br>ENSG00000269639<br>ENSG00000139197<br>ENSG00000133104<br>ENSG00000128710<br>ENSG00000204842<br>ENSG00000180818<br>ENSG00000086717<br>ENSG00000138207<br>ENSG00000186326<br>ENSG00000170786<br>ENSG00000236398<br>ENSG00000221937<br>ENSG00000138795<br>ENSG00000185899<br>ENSG00000221855<br>ENSG00000260696<br>ENSG00000127588<br>ENSG00000127362<br>ENSG00000127364<br>ENSG00000127366<br>ENSG00000257138<br>ENSG00000162572<br>ENSG00000178201<br>ENSG00000152207<br>ENSG00000160349		

ENSG00000121377<br>ENSG00000121314<br>ENSG00000121381<br>ENSG00000121318<br>ENSG00000212128<br>ENSG00000212126<br>ENSG00000255837<br>ENSG00000212124<br>ENSG00000256436<br>ENSG00000226761<br>ENSG00000256188<br>ENSG00000186136<br>ENSG00000169777<br>ENSG00000196131<br>ENSG00000228567<br>ENSG00000273326<br>ENSG00000272712<br>ENSG00000273086<br>ENSG00000272805<br>ENSG00000273457<br>ENSG00000273431<br>ENSG00000273092<br>ENSG00000273404<br>ENSG00000263028<br>ENSG00000263097<br>ENSG00000273359<br>ENSG00000272930<br>ENSG00000262525<br>ENSG00000262111<br>ENSG00000273279<br>ENSG00000273105<br>ENSG00000272952<br>ENSG00000272873<br>ENSG00000262393<br>ENSG00000262379<br>ENSG00000262889<br>ENSG00000262908<br>ENSG00000111319<br>ENSG00000134817<br>ENSG00000166828<br>ENSG00000168447<br>ENSG00000169884<br>ENSG00000064651<br>ENSG00000149488<br>ENSG00000242866<br>ENSG00000105707<br>ENSG00000165091<br>ENSG00000206512<br>ENSG00000224319<br>ENSG00000229680<br>ENSG00000227044<br>ENSG00000204657<br>ENSG00000168158<br>ENSG00000206467<br>ENSG00000229185<br>ENSG00000180090<br>ENSG00000186188<br>ENSG00000173662<br>ENSG00000132563<br>ENSG00000185933<br>ENSG00000165606<br>ENSG00000144481<br>ENSG00000150594<br>ENSG00000104321<br>ENSG00000262304<br>ENSG00000196689<br>ENSG00000154928<br>ENSG00000079257<br>ENSG00000141480<br>ENSG00000160113<br>ENSG00000269095<br>ENSG00000150782<br>ENSG00000164171<br>ENSG00000135919<br>ENSG00000152192<br>ENSG00000146477<br>ENSG00000130287		

ENSG00000066032<br>ENSG00000070831<br>ENSG00000169933<br>ENSG00000134072<br>ENSG00000121853<br>ENSG00000110400<br>ENSG00000124839<br>ENSG00000163110<br>ENSG00000169398<br>ENSG00000185008<br>ENSG00000187122<br>ENSG00000114251<br>ENSG00000145242<br>ENSG00000186340<br>ENSG00000157017<br>ENSG00000141668<br>ENSG00000102359<br>ENSG00000101463<br>ENSG00000169139<br>ENSG00000131409<br>ENSG00000182580<br>ENSG00000063015<br>ENSG00000112759<br>ENSG00000138814<br>ENSG00000100219<br>ENSG00000198785<br>ENSG00000162946<br>ENSG00000117399<br>ENSG00000107954<br>ENSG00000176248<br>ENSG00000072071<br>ENSG00000136848<br>ENSG00000185149<br>ENSG00000267534<br>ENSG00000101489<br>ENSG00000198844		

Gene list for ubiquitous GO terms for human

Membrane organization	Protein folding	RNA splicing
ENSG00000099940	ENSG00000261258	ENSG00000215699
ENSG00000118640	ENSG00000262684	ENSG00000101161
ENSG00000136874	ENSG00000101162	ENSG00000188529
ENSG00000205356	ENSG00000198033	ENSG00000169217
ENSG00000188603	ENSG00000102309	ENSG00000154548
ENSG00000134516	ENSG00000156261	ENSG00000105618
ENSG00000172005	ENSG00000166794	ENSG00000064703
ENSG00000116824	ENSG00000151835	ENSG00000271442
ENSG00000138119	ENSG00000153132	ENSG00000273469
ENSG00000182533	ENSG00000206283	ENSG00000273462
ENSG00000011347	ENSG00000198445	ENSG00000273376
ENSG00000133026	ENSG00000166226	ENSG00000273060
ENSG00000135636	ENSG00000198161	ENSG00000272964
ENSG00000177238	ENSG00000132141	ENSG00000272932
ENSG00000125414	ENSG00000096384	ENSG00000273109
ENSG00000087088	ENSG00000151929	ENSG00000273224
ENSG00000168621	ENSG00000171497	ENSG00000167088
ENSG00000170175	ENSG00000164070	ENSG00000182004
ENSG00000087470	ENSG00000235692	ENSG00000168066
ENSG00000163485	ENSG00000105701	ENSG00000003756
ENSG00000198408	ENSG00000240344	ENSG00000169249
ENSG00000149930	ENSG00000153201	ENSG00000225073
ENSG00000025796	ENSG00000115275	ENSG00000110107
ENSG00000148498	ENSG00000166855	ENSG00000205571
ENSG00000163558	ENSG00000185615	ENSG00000172062
ENSG00000067191	ENSG00000115484	ENSG00000230624
ENSG00000125703	ENSG00000137168	ENSG00000143977
ENSG00000100139	ENSG00000066136	ENSG00000215425
ENSG00000163825	ENSG00000131013	ENSG00000125743
ENSG00000136514	ENSG00000108179	ENSG00000092208
ENSG00000116237	ENSG00000205981	ENSG00000101343
ENSG00000137575	ENSG00000100209	ENSG00000225859
ENSG00000113966	ENSG00000204220	ENSG00000141759
ENSG00000197879	ENSG00000110011	ENSG00000262170

ENSG00000144848	ENSG00000173486	ENSG00000168883
ENSG00000158517	ENSG00000119782	ENSG00000152147
ENSG00000261919	ENSG00000171960	ENSG00000235439
ENSG00000114850	ENSG00000120438	ENSG00000229496
ENSG00000153037	ENSG00000134109	ENSG00000139218
ENSG00000008952	ENSG00000114857	ENSG00000079785
ENSG00000140319	ENSG00000089248	ENSG00000198563
ENSG00000124783	ENSG00000135624	ENSG00000135250
ENSG00000170540	ENSG00000133265	ENSG00000144028
ENSG00000182934	ENSG00000090006	ENSG00000174243
ENSG00000067167	ENSG00000115541	ENSG00000143368
ENSG00000163479	ENSG00000270757	ENSG00000117360
ENSG00000136942	ENSG00000109846	ENSG00000115524
ENSG00000198034	ENSG00000113312	ENSG00000136527
ENSG00000215472	ENSG00000263007	ENSG00000124383
ENSG00000265681	ENSG00000168724	ENSG00000138231
ENSG00000096150	ENSG00000096060	ENSG00000133226
ENSG00000168028	ENSG00000125863	ENSG00000164609
ENSG00000108298	ENSG00000086061	ENSG00000189091
ENSG00000198918	ENSG00000237335	ENSG00000116350
ENSG00000171858	ENSG00000090520	ENSG00000060688
ENSG00000226225	ENSG00000088832	ENSG00000123154
ENSG00000197958	ENSG00000127445	ENSG00000119953
ENSG00000188846	ENSG00000119383	ENSG00000175324
ENSG00000198242	ENSG00000114988	ENSG00000136875
ENSG00000138326	ENSG00000074695	ENSG00000174231
ENSG00000122026	ENSG00000136827	ENSG00000183431
ENSG00000161016	ENSG00000110711	ENSG00000116752
ENSG00000145425	ENSG00000224782	ENSG00000088247
ENSG00000221983	ENSG00000259207	ENSG00000263977
ENSG00000231500	ENSG00000185250	ENSG00000265228
ENSG00000142937	ENSG00000185624	ENSG00000170892
ENSG00000109475	ENSG00000090861	ENSG00000270502
ENSG00000197756	ENSG00000075624	ENSG00000262716
ENSG00000105372	ENSG00000137094	ENSG00000273053
ENSG00000197728	ENSG00000124659	ENSG00000272739
ENSG00000142676	ENSG00000105176	ENSG00000272561
ENSG00000233927	ENSG00000160202	ENSG00000272705
ENSG00000144713	ENSG00000101132	ENSG00000273490
ENSG00000260501	ENSG00000079150	ENSG00000262868
ENSG00000272869	ENSG00000183207	ENSG00000273235
ENSG00000149806	ENSG00000100023	ENSG00000135486
ENSG00000177600	ENSG00000166405	ENSG00000174891
ENSG00000147403	ENSG00000167004	ENSG00000065883
ENSG00000137154	ENSG00000102218	ENSG00000099783
ENSG00000174444	ENSG00000089597	ENSG00000101489
ENSG00000198755	ENSG00000155959	ENSG00000011304
ENSG00000186468	ENSG00000126756	ENSG00000152601
ENSG00000229117	ENSG00000169877	ENSG00000116560
ENSG00000108107	ENSG00000168938	ENSG00000100028
ENSG00000100883	ENSG00000126602	ENSG00000124562
ENSG00000172809	ENSG00000080824	ENSG00000082516
ENSG00000235650	ENSG00000137267	ENSG00000175467
ENSG00000129128	ENSG00000113013	ENSG00000164902
ENSG00000144867	ENSG00000102595	ENSG00000046647
ENSG00000100603	ENSG00000164284	ENSG00000132600
ENSG00000140986	ENSG00000196262	ENSG00000142252
ENSG00000089009	ENSG00000103423	ENSG00000125835
ENSG00000140988	ENSG00000137285	ENSG00000139343
ENSG00000089157	ENSG00000069345	ENSG00000100462
ENSG00000166562	ENSG00000110917	ENSG00000137574
ENSG00000223367	ENSG00000100442	ENSG00000169371
ENSG00000161970	ENSG00000176170	ENSG00000179409
ENSG00000171863	ENSG00000168454	ENSG00000136937
ENSG00000145592	ENSG00000112208	ENSG00000074201
ENSG00000110700	ENSG00000177239	ENSG00000116455
ENSG00000129824	ENSG00000120725	ENSG00000114503
ENSG00000063177	ENSG00000142892	ENSG00000099995
ENSG00000262088	ENSG00000198225	ENSG00000104897
ENSG00000125691	ENSG00000130175	ENSG00000164985
ENSG00000137818	ENSG00000100380	ENSG00000136450
ENSG00000166441	ENSG00000188229	ENSG00000263077
ENSG00000083845	ENSG00000088356	ENSG00000101138
ENSG00000163682	ENSG00000162616	ENSG00000261230
ENSG00000124614	ENSG00000136731	ENSG00000125351
ENSG00000148303	ENSG00000176014	ENSG00000236826
ENSG00000167881	ENSG00000116957	ENSG00000171566

ENSG00000156482<br>ENSG00000162244<br>ENSG00000143742<br>ENSG00000142541<br>ENSG00000142534<br>ENSG00000118997<br>ENSG00000100316<br>ENSG00000241343<br>ENSG00000037241<br>ENSG00000177954<br>ENSG00000174748<br>ENSG00000116251<br>ENSG00000118181<br>ENSG00000008988<br>ENSG00000164587<br>ENSG00000114902<br>ENSG00000213741<br>ENSG00000071082<br>ENSG00000122406<br>ENSG00000115268<br>ENSG00000131469<br>ENSG00000118363<br>ENSG00000149273<br>ENSG00000106803<br>ENSG00000132432<br>ENSG00000134419<br>ENSG00000174780<br>ENSG00000167526<br>ENSG00000184779<br>ENSG00000182774<br>ENSG00000118705<br>ENSG00000147604<br>ENSG00000170889<br>ENSG00000105193<br>ENSG00000114391<br>ENSG00000140612<br>ENSG00000112306<br>ENSG00000058262<br>ENSG00000163902<br>ENSG00000138778<br>ENSG00000130255<br>ENSG00000180879<br>ENSG00000105640<br>ENSG00000244038<br>ENSG00000143947<br>ENSG00000182899<br>ENSG00000269315<br>ENSG00000271572<br>ENSG00000272852<br>ENSG00000269520<br>ENSG00000268963<br>ENSG00000263076<br>ENSG00000273135<br>ENSG00000272943<br>ENSG00000273467<br>ENSG00000273385<br>ENSG00000263332<br>ENSG00000272643<br>ENSG00000272614<br>ENSG00000127603<br>ENSG00000089041<br>ENSG00000198691<br>ENSG00000106299<br>ENSG00000137942<br>ENSG00000127955<br>ENSG00000105971<br>ENSG00000170310<br>ENSG00000102189<br>ENSG00000124333<br>ENSG00000104915<br>ENSG00000134215<br>ENSG00000065135<br>ENSG00000111731<br>ENSG00000118308<br>ENSG00000135823<br>ENSG00000150337<br>ENSG00000155849	ENSG00000143870<br>ENSG00000163956<br>ENSG00000127824<br>ENSG00000166598<br>ENSG00000135924<br>ENSG00000086619<br>ENSG00000129221<br>ENSG00000065485<br>ENSG00000105993<br>ENSG00000187726<br>ENSG00000113068<br>ENSG00000179218<br>ENSG00000166170<br>ENSG00000109519<br>ENSG00000109971<br>ENSG00000150753<br>ENSG00000088298<br>ENSG00000141756<br>ENSG00000140403<br>ENSG00000004478<br>ENSG00000075886<br>ENSG00000132002<br>ENSG00000168259<br>ENSG00000023318<br>ENSG00000172728<br>ENSG00000141556<br>ENSG00000117899<br>ENSG00000182858<br>ENSG00000171530<br>ENSG00000138398<br>ENSG00000146731<br>ENSG00000156735<br>ENSG00000127022<br>ENSG00000123349<br>ENSG00000197930<br>ENSG00000119321<br>ENSG00000258947<br>ENSG00000269058<br>ENSG00000163468<br>ENSG00000106080<br>ENSG00000084072<br>ENSG00000163681<br>ENSG00000136940<br>ENSG00000153015<br>ENSG00000077800<br>ENSG00000113593<br>ENSG00000122642<br>ENSG00000134285<br>ENSG00000236334<br>ENSG00000255854<br>ENSG00000104833<br>ENSG00000129255<br>ENSG00000188603<br>ENSG00000132703<br>ENSG00000123416<br>ENSG00000167552<br>ENSG00000167553<br>ENSG00000255963<br>ENSG00000256374<br>ENSG00000143256<br>ENSG00000158874<br>ENSG00000118217<br>ENSG00000116260<br>ENSG00000116406<br>ENSG00000023572<br>ENSG00000136770<br>ENSG00000262532<br>ENSG00000270913<br>ENSG00000271567<br>ENSG00000270339<br>ENSG00000271001<br>ENSG00000263464<br>ENSG00000270537<br>ENSG00000271091<br>ENSG00000263353<br>ENSG00000268440<br>ENSG00000144381	ENSG00000112081<br>ENSG00000240344<br>ENSG00000105323<br>ENSG00000100109<br>ENSG00000137168<br>ENSG00000150316<br>ENSG00000096401<br>ENSG00000087365<br>ENSG00000125944<br>ENSG00000169976<br>ENSG00000115128<br>ENSG00000163510<br>ENSG00000090060<br>ENSG00000100056<br>ENSG00000171960<br>ENSG00000065978<br>ENSG00000169813<br>ENSG00000260380<br>ENSG00000177700<br>ENSG00000131876<br>ENSG00000164663<br>ENSG00000105705<br>ENSG00000170144<br>ENSG00000224979<br>ENSG00000077312<br>ENSG00000063244<br>ENSG00000204392<br>ENSG00000100603<br>ENSG00000117614<br>ENSG00000162385<br>ENSG00000188342<br>ENSG00000149532<br>ENSG00000218823<br>ENSG00000021776<br>ENSG00000169564<br>ENSG00000177733<br>ENSG00000125870<br>ENSG00000170860<br>ENSG00000072501<br>ENSG00000160201<br>ENSG00000137656<br>ENSG00000205937<br>ENSG00000183684<br>ENSG00000092199<br>ENSG00000159409<br>ENSG00000104852<br>ENSG00000167978<br>ENSG00000076650<br>ENSG00000168438<br>ENSG00000100941<br>ENSG00000089280<br>ENSG00000101811<br>ENSG00000033030<br>ENSG00000119203<br>ENSG00000100142<br>ENSG00000168002<br>ENSG00000109536<br>ENSG00000111786<br>ENSG00000138668<br>ENSG00000147669<br>ENSG00000128534<br>ENSG00000172850<br>ENSG00000140829<br>ENSG00000116754<br>ENSG00000070756<br>ENSG00000071894<br>ENSG00000261974<br>ENSG00000126698<br>ENSG00000126945<br>ENSG00000144231<br>ENSG00000112739<br>ENSG00000100650<br>ENSG00000108654<br>ENSG00000176102<br>ENSG00000135316<br>ENSG00000100410<br>ENSG00000115875

ENSG00000144366	ENSG00000163528	ENSG00000161547
ENSG00000237727	ENSG00000109501	ENSG00000100138
ENSG00000150760	ENSG00000077232	ENSG00000086589
ENSG00000234836	ENSG00000237724	ENSG00000231502
ENSG00000172243	ENSG00000224501	ENSG00000165119
ENSG00000206428	ENSG00000235941	ENSG00000099817
ENSG00000136238	ENSG00000232804	ENSG00000153187
ENSG00000110934	ENSG00000204389	ENSG00000125970
ENSG00000235588	ENSG00000204388	ENSG00000105258
ENSG00000235985	ENSG00000166710	ENSG00000147274
ENSG00000165029	ENSG00000215328	ENSG00000039123
ENSG00000140545	ENSG00000212866	ENSG00000100836
ENSG00000204472	ENSG00000234475	ENSG00000167005
ENSG00000158869	ENSG00000231555	ENSG00000096746
ENSG00000266332	ENSG00000105821	ENSG00000183258
ENSG00000113569	ENSG00000187522	ENSG00000060339
ENSG00000084073	ENSG00000097033	ENSG00000123136
ENSG00000054654	ENSG00000183291	ENSG00000005075
ENSG00000136827	ENSG00000105254	ENSG00000164548
ENSG00000164828	ENSG00000187097	ENSG00000102978
ENSG00000168476	ENSG00000242685	ENSG00000130332
ENSG00000104936	ENSG00000160404	ENSG00000163882
ENSG00000100242	ENSG00000120694	ENSG00000104824
ENSG00000165476	ENSG00000136816	ENSG00000225998
ENSG00000175826	ENSG00000242361	ENSG00000047315
ENSG00000160789	ENSG00000243215	ENSG00000169045
ENSG00000139197	ENSG00000243719	ENSG00000165494
ENSG00000182372	ENSG00000198113	ENSG00000122566
ENSG00000145335	ENSG00000019582	ENSG00000105298
ENSG00000116688	ENSG00000243189	ENSG00000101452
ENSG00000105197	ENSG00000204257	ENSG00000197111
ENSG00000198836	ENSG00000239463	ENSG00000124193
ENSG00000106554	ENSG00000186283	ENSG00000172409
ENSG00000188157	ENSG00000140553	ENSG00000084072
ENSG00000070182	ENSG00000141161	ENSG00000165934
ENSG00000151150	ENSG00000101266	ENSG00000153015
ENSG00000120742	ENSG00000170946	ENSG00000181222
ENSG00000128274	ENSG00000120885	ENSG00000240682
ENSG00000163554	ENSG00000110172	ENSG00000141543
ENSG00000115306	ENSG00000179407	ENSG00000113593
ENSG00000134376	ENSG00000162735	ENSG00000067596
ENSG00000101146	ENSG00000110958	ENSG00000125651
ENSG00000119408	ENSG00000107262	ENSG00000139910
ENSG00000125450		ENSG00000131795
ENSG00000163002		ENSG00000076924
ENSG00000139496		ENSG00000135829
ENSG00000134057		ENSG00000108883
ENSG00000153201		ENSG00000269384
ENSG00000175334		ENSG00000268642
ENSG00000120253		ENSG00000265241
ENSG00000110713		ENSG00000007392
ENSG00000155561		ENSG00000108848
ENSG00000132182		ENSG00000061936
ENSG00000102119		ENSG00000146963
ENSG00000100749		ENSG00000269955
ENSG00000157456		ENSG00000048740
ENSG00000095319		ENSG00000117569
ENSG00000111581		ENSG00000149187
ENSG00000124789		ENSG00000154743
ENSG00000138750		ENSG00000149743
ENSG00000126883		ENSG00000182173
ENSG00000075188		ENSG00000100220
ENSG00000069248		ENSG00000198860
ENSG00000108559		ENSG00000185272
ENSG00000213024		ENSG00000259956
ENSG00000170312		ENSG00000132819
ENSG00000085415		ENSG00000128739
ENSG00000102900		ENSG00000267483
ENSG00000093000		ENSG00000150459
ENSG00000136243		ENSG00000108819
ENSG00000094914		ENSG00000130520
ENSG00000030066		ENSG00000197976
ENSG00000196313		ENSG00000161981
ENSG00000166851		ENSG00000090470
ENSG00000119638		ENSG00000168566
ENSG00000047410		ENSG00000110844
ENSG00000262122		ENSG00000159140

ENSG00000268557<br>ENSG00000113575<br>ENSG00000176915<br>ENSG00000221914<br>ENSG00000105568<br>ENSG00000128294<br>ENSG00000175294<br>ENSG00000104755<br>ENSG00000120160<br>ENSG00000258484<br>ENSG00000182264<br>ENSG00000124812<br>ENSG00000106304<br>ENSG00000114547<br>ENSG00000010278<br>ENSG00000188488<br>ENSG00000168958<br>ENSG00000171109<br>ENSG00000030110<br>ENSG00000141385<br>ENSG00000214253<br>ENSG00000179598<br>ENSG00000124198<br>ENSG00000066777<br>ENSG00000157107<br>ENSG00000113734<br>ENSG00000175073<br>ENSG00000114698<br>ENSG00000163746<br>ENSG00000188313<br>ENSG00000177119<br>ENSG00000187838<br>ENSG00000261921<br>ENSG00000068001<br>ENSG00000130202<br>ENSG00000183087<br>ENSG00000164850<br>ENSG00000131238<br>ENSG00000075711<br>ENSG00000143553<br>ENSG00000171862<br>ENSG00000124920<br>ENSG00000103089<br>ENSG00000142168<br>ENSG00000105221<br>ENSG00000072415<br>ENSG00000169247<br>ENSG00000142208<br>ENSG00000104419<br>ENSG00000150093<br>ENSG00000132563<br>ENSG00000145362<br>ENSG00000090020<br>ENSG00000170348<br>ENSG00000102125<br>ENSG00000162600<br>ENSG00000168924<br>ENSG00000159685<br>ENSG00000268904<br>ENSG00000111057<br>ENSG00000084733<br>ENSG00000174483<br>ENSG00000101246<br>ENSG00000220205<br>ENSG00000113384<br>ENSG00000137177<br>ENSG00000167964<br>ENSG00000168899<br>ENSG00000168461<br>ENSG00000049245<br>ENSG00000123240<br>ENSG00000125124<br>ENSG00000144674<br>ENSG00000117475<br>ENSG00000117533<br>ENSG00000196924<br>ENSG00000105737		ENSG00000167258<br>ENSG00000164167<br>ENSG00000145833<br>ENSG00000257413<br>ENSG00000213079<br>ENSG00000163634<br>ENSG00000146457<br>ENSG00000109819<br>ENSG00000100296<br>ENSG00000139767<br>ENSG00000109606<br>ENSG00000064607<br>ENSG00000100319<br>ENSG00000173933<br>ENSG00000173914<br>ENSG00000103067<br>ENSG00000112531<br>ENSG00000125676<br>ENSG00000196504<br>ENSG00000206486<br>ENSG00000075292<br>ENSG00000242875<br>ENSG00000233418<br>ENSG00000134748<br>ENSG00000234414<br>ENSG00000242389<br>ENSG00000096063<br>ENSG00000164944<br>ENSG00000124380<br>ENSG00000244395<br>ENSG00000163156<br>ENSG00000169800<br>ENSG00000104413<br>ENSG00000104859<br>ENSG00000130810<br>ENSG00000109111<br>ENSG00000226941<br>ENSG00000179837<br>ENSG00000113575<br>ENSG00000179950<br>ENSG00000100320<br>ENSG00000079134<br>ENSG00000139675<br>ENSG00000182872<br>ENSG00000233049<br>ENSG00000131652<br>ENSG00000138433<br>ENSG00000108349<br>ENSG00000126653<br>ENSG00000163166<br>ENSG00000158941<br>ENSG00000163605<br>ENSG00000140830<br>ENSG00000076770<br>ENSG00000111196<br>ENSG00000117751<br>ENSG00000126461<br>ENSG00000139793<br>ENSG00000185246<br>ENSG00000132485<br>ENSG00000184937<br>ENSG00000051596<br>ENSG00000108561<br>ENSG00000213782<br>ENSG00000182196<br>ENSG00000078328<br>ENSG00000070495<br>ENSG00000139168<br>ENSG00000226171<br>ENSG00000161265<br>ENSG00000231377<br>ENSG00000184209<br>ENSG00000213516<br>ENSG00000084463<br>ENSG00000100813<br>ENSG00000162664<br>ENSG00000137948

ENSG00000164885		ENSG00000204560
ENSG00000197283		ENSG00000106344
ENSG00000107404		ENSG00000109971
ENSG00000187391		ENSG00000023734
ENSG00000227460		ENSG00000119707
ENSG00000119185		ENSG00000120948
ENSG00000100151		ENSG00000203867
ENSG00000175920		ENSG00000105568
ENSG00000141837		ENSG00000155966
ENSG00000149269		ENSG00000185946
ENSG00000134569		ENSG00000147140
ENSG00000030304		ENSG00000131051
ENSG00000272706		ENSG00000134186
ENSG00000269329		ENSG00000138398
ENSG00000082397		ENSG00000054118
ENSG00000121966		ENSG00000103005
ENSG00000137801		ENSG00000181817
ENSG00000158156		ENSG00000119314
ENSG00000066056		ENSG00000165630
ENSG00000073969		ENSG00000132792
ENSG00000187616		ENSG00000134453
ENSG00000177370		ENSG00000239665
ENSG00000134809		ENSG00000083896
ENSG00000100575		ENSG00000116954
ENSG00000170606		ENSG00000260485
ENSG00000100216		ENSG00000131981
ENSG00000080824		ENSG00000167281
ENSG00000100347		ENSG00000153006
ENSG00000166128		ENSG00000140939
ENSG00000034693		ENSG00000106355
ENSG00000215193		ENSG00000116830
ENSG00000121680		ENSG00000164610
ENSG00000162735		ENSG00000153914
ENSG00000198356		ENSG00000233561
ENSG00000130635		ENSG00000116679
ENSG00000137409		ENSG00000271352
ENSG00000186417		ENSG00000269754
ENSG00000091129		ENSG00000120688
ENSG00000163531		ENSG00000170935
ENSG00000151466		ENSG00000110063
ENSG00000160460		ENSG00000178607
ENSG00000184144		
ENSG00000120049		
ENSG00000174469		
ENSG00000206190		
ENSG00000058063		
ENSG00000081923		
ENSG00000104783		
ENSG00000112697		
ENSG00000118322		
ENSG00000054793		
ENSG00000166377		
ENSG00000124406		
ENSG00000145246		
ENSG00000068650		
ENSG00000101974		
ENSG00000130270		
ENSG00000262782		
ENSG00000260283		
ENSG00000196329		
ENSG00000015475		
ENSG00000108179		
ENSG00000176171		
ENSG00000002330		
ENSG00000141682		
ENSG00000171791		
ENSG00000171552		
ENSG00000197386		
ENSG00000173786		
ENSG00000141510		
ENSG00000196535		
ENSG00000184432		
ENSG00000101199		
ENSG00000111481		
ENSG00000105669		
ENSG00000129083		
ENSG00000107862		

ENSG00000143761<br>ENSG00000095139<br>ENSG00000086598<br>ENSG00000184840<br>ENSG00000181789<br>ENSG00000122218<br>ENSG00000269382<br>ENSG00000036257<br>ENSG00000152700<br>ENSG00000113615<br>ENSG00000138802<br>ENSG00000157020<br>ENSG00000138073<br>ENSG00000100934<br>ENSG00000150961<br>ENSG00000148396<br>ENSG00000138674<br>ENSG00000176986<br>ENSG00000120341<br>ENSG00000117153<br>ENSG00000145506<br>ENSG00000167461<br>ENSG00000042753<br>ENSG00000085832<br>ENSG00000113282<br>ENSG00000153071<br>ENSG00000006125<br>ENSG00000130643<br>ENSG00000011405<br>ENSG00000065609<br>ENSG00000170091<br>ENSG00000168824<br>ENSG00000073921<br>ENSG00000130475<br>ENSG00000127946<br>ENSG00000263334<br>ENSG00000270478<br>ENSG00000138768<br>ENSG00000162236<br>ENSG00000105829<br>ENSG00000102003<br>ENSG00000065000<br>ENSG00000268353<br>ENSG00000166579<br>ENSG00000007168<br>ENSG00000163626<br>ENSG00000175077<br>ENSG00000155463<br>ENSG00000198471<br>ENSG00000116032<br>ENSG00000068615<br>ENSG00000146648<br>ENSG00000163913<br>ENSG00000157796<br>ENSG00000176101<br>ENSG00000128602<br>ENSG00000143156<br>ENSG00000187446<br>ENSG00000185053<br>ENSG00000128298<br>ENSG00000197971<br>ENSG00000110375<br>ENSG00000087253<br>ENSG00000147588<br>ENSG00000115355<br>ENSG00000184056<br>ENSG00000197548<br>ENSG00000135604<br>ENSG00000166900<br>ENSG00000085382<br>ENSG00000115155<br>ENSG00000105402<br>ENSG00000092531<br>ENSG00000182446<br>ENSG00000108433<br>ENSG00000100568<br>ENSG00000101558		

ENSG00000134265<br>ENSG00000029725<br>ENSG00000138279<br>ENSG00000115271<br>ENSG00000164935<br>ENSG00000079841<br>ENSG00000035720<br>ENSG00000183155<br>ENSG00000070495<br>ENSG00000105974<br>ENSG00000100266<br>ENSG00000244405<br>ENSG00000103423<br>ENSG00000136816<br>ENSG00000143337<br>ENSG00000233348<br>ENSG00000102178<br>ENSG00000239857<br>ENSG00000229524<br>ENSG00000096155<br>ENSG00000227761<br>ENSG00000228760<br>ENSG00000234651<br>ENSG00000204463<br>ENSG00000269715<br>ENSG00000109472<br>ENSG00000092529<br>ENSG00000124507<br>ENSG00000174705<br>ENSG00000167476<br>ENSG00000073712<br>ENSG00000197535<br>ENSG00000135525<br>ENSG00000177108<br>ENSG00000103769<br>ENSG00000184307<br>ENSG00000151025<br>ENSG00000137145<br>ENSG00000024422<br>ENSG00000188888<br>ENSG00000039068<br>ENSG00000206480<br>ENSG00000116171<br>ENSG00000206379<br>ENSG00000166289<br>ENSG00000067606<br>ENSG00000236271<br>ENSG00000131477<br>ENSG00000181092<br>ENSG00000143545<br>ENSG00000230143<br>ENSG00000122679<br>ENSG00000132329<br>ENSG00000129682<br>ENSG00000166257<br>ENSG00000137312<br>ENSG00000169860<br>ENSG00000004948<br>ENSG00000156735<br>ENSG00000155256<br>ENSG00000130545<br>ENSG00000232280<br>ENSG00000260825<br>ENSG00000175115<br>ENSG00000013016<br>ENSG00000182718<br>ENSG00000029534<br>ENSG00000143398<br>ENSG00000088992<br>ENSG00000101608<br>ENSG00000099864<br>ENSG00000185920<br>ENSG00000132376<br>ENSG00000179364<br>ENSG00000271374<br>ENSG00000130702<br>ENSG00000162552		

ENSG00000174827<br>ENSG00000265111<br>ENSG00000186439<br>ENSG00000179331<br>ENSG00000109113<br>ENSG00000139832<br>ENSG00000075785<br>ENSG00000170855<br>ENSG00000169230<br>ENSG00000169760<br>ENSG00000101463<br>ENSG00000179915<br>ENSG00000110076<br>ENSG00000196338<br>ENSG00000169992<br>ENSG00000146938<br>ENSG00000153707<br>ENSG00000169306<br>ENSG00000132535<br>ENSG00000251322<br>ENSG00000189056<br>ENSG00000087250<br>ENSG00000173548<br>ENSG00000165912<br>ENSG00000064655<br>ENSG00000140983<br>ENSG00000215012<br>ENSG00000126858<br>ENSG00000196072<br>ENSG00000171169<br>ENSG00000159445<br>ENSG00000115966<br>ENSG00000153094<br>ENSG00000165283		

Gene list for specific GO terms for mouse

Neurological system process	Spermatogenesis	Xenobiotic metabolic process
ENSMUSG00000007097<br>ENSMUSG00000030500<br>ENSMUSG00000032959<br>ENSMUSG00000029071<br>ENSMUSG00000000560<br>ENSMUSG00000022489<br>ENSMUSG00000039059<br>ENSMUSG00000026986<br>ENSMUSG00000056115<br>ENSMUSG00000052850<br>ENSMUSG00000037140<br>ENSMUSG00000058250<br>ENSMUSG00000047102<br>ENSMUSG00000051917<br>ENSMUSG00000046652<br>ENSMUSG00000056203<br>ENSMUSG00000048284<br>ENSMUSG00000028777<br>ENSMUSG00000043865<br>ENSMUSG00000034837<br>ENSMUSG00000009108<br>ENSMUSG00000066319<br>ENSMUSG00000054497<br>ENSMUSG00000053389<br>ENSMUSG00000056901<br>ENSMUSG00000053217<br>ENSMUSG00000057381<br>ENSMUSG00000062952<br>ENSMUSG00000071147<br>ENSMUSG00000033355<br>ENSMUSG00000048827<br>ENSMUSG00000037578<br>ENSMUSG00000028738<br>ENSMUSG00000029072<br>ENSMUSG00000021478<br>ENSMUSG00000021250<br>ENSMUSG00000027950	ENSMUSG00000062300<br>ENSMUSG00000038709<br>ENSMUSG00000020193<br>ENSMUSG00000028264<br>ENSMUSG00000027528<br>ENSMUSG00000030059<br>ENSMUSG00000071359<br>ENSMUSG00000020745<br>ENSMUSG00000028575<br>ENSMUSG00000026159<br>ENSMUSG00000017195<br>ENSMUSG00000027722<br>ENSMUSG00000048516<br>ENSMUSG00000037001<br>ENSMUSG00000002015<br>ENSMUSG00000020453<br>ENSMUSG00000027490<br>ENSMUSG00000022021<br>ENSMUSG00000035517<br>ENSMUSG00000003549<br>ENSMUSG00000020451<br>ENSMUSG00000025902<br>ENSMUSG00000020661<br>ENSMUSG00000019893<br>ENSMUSG00000029516<br>ENSMUSG00000032000<br>ENSMUSG00000049721<br>ENSMUSG00000042063<br>ENSMUSG00000020064<br>ENSMUSG00000095833<br>ENSMUSG00000094727<br>ENSMUSG00000020063<br>ENSMUSG00000021944<br>ENSMUSG00000063889<br>ENSMUSG00000028426<br>ENSMUSG00000024107<br>ENSMUSG00000032937	ENSMUSG00000040170<br>ENSMUSG00000025069<br>ENSMUSG00000021575<br>ENSMUSG00000030711<br>ENSMUSG00000021259<br>ENSMUSG00000060803<br>ENSMUSG00000058216<br>ENSMUSG00000024847<br>ENSMUSG00000022809<br>ENSMUSG00000021213<br>ENSMUSG00000021211<br>ENSMUSG00000027698<br>ENSMUSG00000017950<br>ENSMUSG00000019256<br>ENSMUSG00000029269<br>ENSMUSG00000024087<br>ENSMUSG00000097830<br>ENSMUSG00000097663<br>ENSMUSG00000097319<br>ENSMUSG00000029759<br>ENSMUSG00000031574<br>ENSMUSG00000032315<br>ENSMUSG00000003559<br>ENSMUSG00000026959<br>ENSMUSG00000052974<br>ENSMUSG00000018796<br>ENSMUSG00000025068<br>ENSMUSG00000004032<br>ENSMUSG00000004035<br>ENSMUSG00000027890<br>ENSMUSG00000028199<br>ENSMUSG00000089960

ENSMUSG00000056755<br>ENSMUSG00000026959<br>ENSMUSG00000034472<br>ENSMUSG00000025889<br>ENSMUSG00000032259<br>ENSMUSG00000028969<br>ENSMUSG00000020178<br>ENSMUSG00000049796<br>ENSMUSG00000025496<br>ENSMUSG00000022705<br>ENSMUSG00000073465<br>ENSMUSG00000023826<br>ENSMUSG00000028964<br>ENSMUSG00000000214<br>ENSMUSG00000021680<br>ENSMUSG00000045573<br>ENSMUSG00000019874<br>ENSMUSG00000031393<br>ENSMUSG00000045871<br>ENSMUSG00000028020<br>ENSMUSG00000059003<br>ENSMUSG00000052613<br>ENSMUSG00000002771<br>ENSMUSG00000001988<br>ENSMUSG00000030209<br>ENSMUSG00000021010<br>ENSMUSG00000000263<br>ENSMUSG00000030669<br>ENSMUSG00000035458<br>ENSMUSG00000045875<br>ENSMUSG00000027335<br>ENSMUSG00000050541<br>ENSMUSG00000030525<br>ENSMUSG00000032575<br>ENSMUSG00000049115<br>ENSMUSG00000031980<br>ENSMUSG00000053719<br>ENSMUSG00000023809<br>ENSMUSG00000068122<br>ENSMUSG00000012520<br>ENSMUSG00000021217<br>ENSMUSG00000026103<br>ENSMUSG00000031302<br>ENSMUSG00000040610<br>ENSMUSG00000035946<br>ENSMUSG00000063887<br>ENSMUSG00000038718<br>ENSMUSG00000051790<br>ENSMUSG00000042429<br>ENSMUSG00000052512<br>ENSMUSG00000020704<br>ENSMUSG00000029503<br>ENSMUSG00000030043<br>ENSMUSG00000015405<br>ENSMUSG00000006818<br>ENSMUSG00000023328<br>ENSMUSG00000002104<br>ENSMUSG00000036422<br>ENSMUSG00000000058<br>ENSMUSG00000044014<br>ENSMUSG00000068615<br>ENSMUSG00000024109<br>ENSMUSG00000003378<br>ENSMUSG00000047879<br>ENSMUSG00000035383<br>ENSMUSG00000045731<br>ENSMUSG00000033214<br>ENSMUSG00000020827<br>ENSMUSG00000033981<br>ENSMUSG00000028456<br>ENSMUSG00000024897<br>ENSMUSG00000034891<br>ENSMUSG00000063142<br>ENSMUSG00000047910<br>ENSMUSG00000024211<br>ENSMUSG00000057914<br>ENSMUSG00000041936	ENSMUSG00000031447<br>ENSMUSG00000029279<br>ENSMUSG00000010796<br>ENSMUSG00000019878<br>ENSMUSG00000036912<br>ENSMUSG00000016626<br>ENSMUSG00000026567<br>ENSMUSG00000063415<br>ENSMUSG00000020475<br>ENSMUSG00000047003<br>ENSMUSG00000063049<br>ENSMUSG00000008482<br>ENSMUSG00000005447<br>ENSMUSG00000040629<br>ENSMUSG00000021477<br>ENSMUSG00000050052<br>ENSMUSG00000022982<br>ENSMUSG00000056223<br>ENSMUSG00000002602<br>ENSMUSG00000020609<br>ENSMUSG00000059625<br>ENSMUSG00000002768<br>ENSMUSG00000027433<br>ENSMUSG00000030771<br>ENSMUSG00000023977<br>ENSMUSG00000049932<br>ENSMUSG00000063281<br>ENSMUSG00000036551<br>ENSMUSG00000054717<br>ENSMUSG00000015365<br>ENSMUSG00000041298<br>ENSMUSG00000042156<br>ENSMUSG00000001157<br>ENSMUSG00000039781<br>ENSMUSG00000042650<br>ENSMUSG00000013973<br>ENSMUSG00000006418<br>ENSMUSG00000044201<br>ENSMUSG00000030968<br>ENSMUSG00000019732<br>ENSMUSG00000079681<br>ENSMUSG00000024352<br>ENSMUSG00000029502<br>ENSMUSG00000044288<br>ENSMUSG00000007107<br>ENSMUSG00000028389<br>ENSMUSG00000041147<br>ENSMUSG00000027381<br>ENSMUSG00000031493<br>ENSMUSG00000056820<br>ENSMUSG00000024837<br>ENSMUSG00000036529<br>ENSMUSG00000049123<br>ENSMUSG00000020777<br>ENSMUSG00000007033<br>ENSMUSG00000000567<br>ENSMUSG00000014361<br>ENSMUSG00000041550<br>ENSMUSG00000047904<br>ENSMUSG00000062438<br>ENSMUSG00000095801<br>ENSMUSG00000021569<br>ENSMUSG00000000811<br>ENSMUSG00000010592<br>ENSMUSG00000028458<br>ENSMUSG00000027793<br>ENSMUSG00000020317<br>ENSMUSG00000003131<br>ENSMUSG00000002190<br>ENSMUSG00000029778<br>ENSMUSG00000025056<br>ENSMUSG00000027794<br>ENSMUSG00000039209<br>ENSMUSG00000025081<br>ENSMUSG00000078938<br>ENSMUSG00000034401<br>ENSMUSG00000027298	

ENSMUSG00000008892	ENSMUSG00000035246	
ENSMUSG00000025551	ENSMUSG00000079509	
ENSMUSG00000019433	ENSMUSG00000032076	
ENSMUSG00000020308	ENSMUSG00000074704	
ENSMUSG00000034656	ENSMUSG00000020325	
ENSMUSG00000026322	ENSMUSG00000050957	
ENSMUSG00000031144	ENSMUSG00000008429	
ENSMUSG00000001260	ENSMUSG00000056155	
ENSMUSG00000066392	ENSMUSG00000043050	
ENSMUSG00000070570	ENSMUSG00000050058	
ENSMUSG00000001018	ENSMUSG00000038015	
ENSMUSG00000024798	ENSMUSG00000022501	
ENSMUSG00000020961	ENSMUSG00000024176	
ENSMUSG00000039953	ENSMUSG00000024905	
ENSMUSG00000078816	ENSMUSG00000021590	
ENSMUSG00000027071	ENSMUSG00000024392	
ENSMUSG00000020436	ENSMUSG00000031990	
ENSMUSG00000055078	ENSMUSG00000036478	
ENSMUSG00000063239	ENSMUSG00000031991	
ENSMUSG00000020745	ENSMUSG00000041323	
ENSMUSG00000030519	ENSMUSG00000060794	
ENSMUSG00000036510	ENSMUSG00000005893	
ENSMUSG00000003279	ENSMUSG00000028410	
ENSMUSG00000051111	ENSMUSG00000003814	
ENSMUSG00000053025	ENSMUSG00000021038	
ENSMUSG00000034520	ENSMUSG00000020530	
ENSMUSG00000020866	ENSMUSG00000046532	
ENSMUSG00000006932	ENSMUSG00000029469	
ENSMUSG00000030201	ENSMUSG00000033644	
ENSMUSG00000039110	ENSMUSG00000020097	
ENSMUSG00000021919	ENSMUSG00000033985	
ENSMUSG00000049583	ENSMUSG00000053470	
ENSMUSG00000017740	ENSMUSG00000039224	
ENSMUSG00000052572	ENSMUSG00000022556	
ENSMUSG00000021647	ENSMUSG00000001761	
ENSMUSG00000000617	ENSMUSG00000027938	
ENSMUSG00000061762	ENSMUSG00000031552	
ENSMUSG00000023064	ENSMUSG00000040828	
ENSMUSG00000004931	ENSMUSG00000040268	
ENSMUSG00000033768	ENSMUSG00000019996	
ENSMUSG00000070687	ENSMUSG00000007659	
ENSMUSG00000020402	ENSMUSG00000029475	
ENSMUSG00000038486	ENSMUSG00000024207	
ENSMUSG00000051331	ENSMUSG00000020513	
ENSMUSG00000029054	ENSMUSG00000021499	
ENSMUSG00000056073	ENSMUSG00000025977	
ENSMUSG00000020524	ENSMUSG00000032591	
ENSMUSG00000022523	ENSMUSG00000028427	
ENSMUSG00000062151	ENSMUSG00000006527	
ENSMUSG00000034593	ENSMUSG00000025289	
ENSMUSG00000033577	ENSMUSG00000021264	
ENSMUSG00000049511	ENSMUSG00000050199	
ENSMUSG00000097449	ENSMUSG00000006345	
ENSMUSG00000059602	ENSMUSG00000075706	
ENSMUSG00000024261	ENSMUSG00000036840	
ENSMUSG00000030093	ENSMUSG00000029155	
ENSMUSG00000019906	ENSMUSG00000007646	
ENSMUSG00000035864	ENSMUSG00000003873	
ENSMUSG00000027162	ENSMUSG00000023015	
ENSMUSG00000003872	ENSMUSG00000031661	
ENSMUSG00000026797	ENSMUSG00000000085	
ENSMUSG00000028657	ENSMUSG00000030041	
ENSMUSG00000035390	ENSMUSG00000044405	
ENSMUSG00000004113	ENSMUSG00000030069	
ENSMUSG00000027273	ENSMUSG00000025482	
ENSMUSG00000009394	ENSMUSG00000002384	
ENSMUSG00000037217	ENSMUSG00000039329	
ENSMUSG00000038128	ENSMUSG00000032726	
ENSMUSG00000013663	ENSMUSG00000070999	
ENSMUSG00000063077	ENSMUSG00000005672	
ENSMUSG00000032186	ENSMUSG00000022783	
ENSMUSG00000066279	ENSMUSG00000028441	
ENSMUSG00000027792	ENSMUSG00000050612	
ENSMUSG00000048330	ENSMUSG00000038932	
ENSMUSG00000023945	ENSMUSG00000039335	
ENSMUSG00000032303	ENSMUSG00000020150	
ENSMUSG00000027577	ENSMUSG00000069565	

ENSMUSG00000046159<br>ENSMUSG00000041189<br>ENSMUSG00000031840<br>ENSMUSG00000033730<br>ENSMUSG00000027470<br>ENSMUSG00000049107<br>ENSMUSG00000027107<br>ENSMUSG00000032773<br>ENSMUSG00000013089<br>ENSMUSG00000047139<br>ENSMUSG00000030761<br>ENSMUSG00000038084<br>ENSMUSG00000052214<br>ENSMUSG00000025900<br>ENSMUSG00000028012<br>ENSMUSG00000024140<br>ENSMUSG00000000724<br>ENSMUSG00000021396<br>ENSMUSG00000022125<br>ENSMUSG00000046049<br>ENSMUSG00000025921<br>ENSMUSG00000020599<br>ENSMUSG00000025389<br>ENSMUSG00000031394<br>ENSMUSG00000026317<br>ENSMUSG00000036192<br>ENSMUSG00000002058<br>ENSMUSG00000029491<br>ENSMUSG00000023978<br>ENSMUSG00000024041<br>ENSMUSG00000023979<br>ENSMUSG00000023982<br>ENSMUSG00000053773<br>ENSMUSG00000032556<br>ENSMUSG00000022483<br>ENSMUSG00000023267<br>ENSMUSG00000026840<br>ENSMUSG00000028003<br>ENSMUSG00000044708<br>ENSMUSG00000033080<br>ENSMUSG00000037408<br>ENSMUSG00000061524<br>ENSMUSG00000057132<br>ENSMUSG00000025386<br>ENSMUSG00000026114<br>ENSMUSG00000066975<br>ENSMUSG00000042240<br>ENSMUSG00000025350<br>ENSMUSG00000058831<br>ENSMUSG00000021123<br>ENSMUSG00000056494<br>ENSMUSG00000031142<br>ENSMUSG00000036446<br>ENSMUSG00000075410<br>ENSMUSG00000043850<br>ENSMUSG00000069170<br>ENSMUSG00000060890<br>ENSMUSG00000026609<br>ENSMUSG00000067220<br>ENSMUSG00000024842<br>ENSMUSG00000049353<br>ENSMUSG00000052911<br>ENSMUSG00000031737<br>ENSMUSG00000021007<br>ENSMUSG00000027966<br>ENSMUSG00000042064<br>ENSMUSG00000020907<br>ENSMUSG00000024992<br>ENSMUSG00000025952<br>ENSMUSG00000030523<br>ENSMUSG00000022243<br>ENSMUSG00000039194<br>ENSMUSG00000043972<br>ENSMUSG00000039474<br>ENSMUSG00000028125<br>ENSMUSG00000028174<br>ENSMUSG00000031174	ENSMUSG00000041912<br>ENSMUSG00000028137<br>ENSMUSG00000029104<br>ENSMUSG00000028647<br>ENSMUSG00000038498<br>ENSMUSG00000048731<br>ENSMUSG00000027480<br>ENSMUSG00000028894<br>ENSMUSG00000024990<br>ENSMUSG00000044933<br>ENSMUSG00000031786<br>ENSMUSG00000009628<br>ENSMUSG00000030451<br>ENSMUSG00000014030<br>ENSMUSG00000032498<br>ENSMUSG00000072663<br>ENSMUSG00000024922<br>ENSMUSG00000005493<br>ENSMUSG00000040489<br>ENSMUSG00000030254<br>ENSMUSG00000054003<br>ENSMUSG00000023830<br>ENSMUSG00000053153<br>ENSMUSG00000037716<br>ENSMUSG00000068037<br>ENSMUSG00000032431<br>ENSMUSG00000028796<br>ENSMUSG00000053030<br>ENSMUSG00000026182<br>ENSMUSG00000032921<br>ENSMUSG00000000411<br>ENSMUSG00000046723<br>ENSMUSG00000071937<br>ENSMUSG00000056758<br>ENSMUSG00000002799<br>ENSMUSG00000033210<br>ENSMUSG00000029848<br>ENSMUSG00000030189<br>ENSMUSG00000041566<br>ENSMUSG00000038797<br>ENSMUSG00000022429<br>ENSMUSG00000056899<br>ENSMUSG00000031893<br>ENSMUSG00000029423<br>ENSMUSG00000017767<br>ENSMUSG00000020867<br>ENSMUSG00000044787<br>ENSMUSG00000031347<br>ENSMUSG00000039110<br>ENSMUSG00000038057<br>ENSMUSG00000078796<br>ENSMUSG00000014767<br>ENSMUSG00000022652<br>ENSMUSG00000051977<br>ENSMUSG00000036196<br>ENSMUSG00000079470<br>ENSMUSG00000036545<br>ENSMUSG00000027660<br>ENSMUSG00000038994<br>ENSMUSG00000037625<br>ENSMUSG00000031722<br>ENSMUSG00000007907<br>ENSMUSG00000006731<br>ENSMUSG00000032280<br>ENSMUSG00000048003<br>ENSMUSG00000051965<br>ENSMUSG00000040891<br>ENSMUSG00000045378<br>ENSMUSG00000028109<br>ENSMUSG00000032405<br>ENSMUSG00000020390<br>ENSMUSG00000041138<br>ENSMUSG00000020694<br>ENSMUSG00000025407<br>ENSMUSG00000041431<br>ENSMUSG00000035431<br>ENSMUSG00000050623	

ENSMUSG00000044375<br>ENSMUSG00000037446<br>ENSMUSG00000020890<br>ENSMUSG00000001506<br>ENSMUSG00000003848<br>ENSMUSG00000051228<br>ENSMUSG00000064330<br>ENSMUSG00000032245<br>ENSMUSG00000009030<br>ENSMUSG00000021799<br>ENSMUSG00000026239<br>ENSMUSG00000029410<br>ENSMUSG00000021804<br>ENSMUSG00000030324<br>ENSMUSG00000027679<br>ENSMUSG00000056055<br>ENSMUSG00000034452<br>ENSMUSG00000070337<br>ENSMUSG00000019803<br>ENSMUSG00000071648<br>ENSMUSG00000042282<br>ENSMUSG00000006007<br>ENSMUSG00000097687<br>ENSMUSG00000038600<br>ENSMUSG00000078630<br>ENSMUSG00000014686<br>ENSMUSG00000062372<br>ENSMUSG00000005371<br>ENSMUSG00000025418<br>ENSMUSG00000025401<br>ENSMUSG00000039488<br>ENSMUSG00000006269<br>ENSMUSG00000023033<br>ENSMUSG00000063919<br>ENSMUSG00000024034<br>ENSMUSG00000031628<br>ENSMUSG00000037705<br>ENSMUSG00000021779<br>ENSMUSG00000041235<br>ENSMUSG00000031028<br>ENSMUSG00000075267<br>ENSMUSG00000026805<br>ENSMUSG00000022982<br>ENSMUSG00000024411<br>ENSMUSG00000022114<br>ENSMUSG00000051367<br>ENSMUSG00000069581<br>ENSMUSG00000042678<br>ENSMUSG00000011751<br>ENSMUSG00000024979<br>ENSMUSG00000019935<br>ENSMUSG00000030905<br>ENSMUSG00000029821<br>ENSMUSG00000096472<br>ENSMUSG00000034990<br>ENSMUSG00000031688<br>ENSMUSG00000029844<br>ENSMUSG00000024456<br>ENSMUSG00000029015<br>ENSMUSG00000024497<br>ENSMUSG00000029205<br>ENSMUSG00000057322<br>ENSMUSG00000012819<br>ENSMUSG00000039137<br>ENSMUSG00000027463<br>ENSMUSG00000068082<br>ENSMUSG00000021359<br>ENSMUSG00000015968<br>ENSMUSG00000045288<br>ENSMUSG00000025716<br>ENSMUSG00000073678<br>ENSMUSG00000032818<br>ENSMUSG00000063856<br>ENSMUSG00000031565<br>ENSMUSG00000022676<br>ENSMUSG00000028631<br>ENSMUSG00000024182	ENSMUSG00000019834<br>ENSMUSG00000028760<br>ENSMUSG00000020681<br>ENSMUSG00000026650<br>ENSMUSG00000041165<br>ENSMUSG00000063972<br>ENSMUSG00000024116<br>ENSMUSG00000060499<br>ENSMUSG00000031065<br>ENSMUSG00000041035<br>ENSMUSG00000036211<br>ENSMUSG00000016758<br>ENSMUSG00000049539<br>ENSMUSG00000040013<br>ENSMUSG00000037270<br>ENSMUSG00000052155<br>ENSMUSG00000040693<br>ENSMUSG00000021758<br>ENSMUSG00000022508<br>ENSMUSG00000037992<br>ENSMUSG00000037017<br>ENSMUSG00000033952<br>ENSMUSG00000026360<br>ENSMUSG00000043289<br>ENSMUSG00000056131<br>ENSMUSG00000032359<br>ENSMUSG00000051306<br>ENSMUSG00000005202<br>ENSMUSG00000051176<br>ENSMUSG00000001558<br>ENSMUSG00000031922<br>ENSMUSG00000043648<br>ENSMUSG00000032959<br>ENSMUSG00000071470<br>ENSMUSG00000028453<br>ENSMUSG00000040829<br>ENSMUSG00000035984<br>ENSMUSG00000013787<br>ENSMUSG00000026790<br>ENSMUSG00000059970<br>ENSMUSG00000029707<br>ENSMUSG00000061099<br>ENSMUSG00000005883<br>ENSMUSG00000085576<br>ENSMUSG00000023000<br>ENSMUSG00000072770<br>ENSMUSG00000027939<br>ENSMUSG00000032101<br>ENSMUSG00000033487<br>ENSMUSG00000003068<br>ENSMUSG00000020486<br>ENSMUSG00000039041<br>ENSMUSG00000037514<br>ENSMUSG00000067909<br>ENSMUSG00000030450<br>ENSMUSG00000062078<br>ENSMUSG00000044407<br>ENSMUSG00000037196<br>ENSMUSG00000002324<br>ENSMUSG00000028572<br>ENSMUSG00000045521<br>ENSMUSG00000025235<br>ENSMUSG00000000365<br>ENSMUSG00000005506<br>ENSMUSG00000027274<br>ENSMUSG00000030641<br>ENSMUSG00000040841<br>ENSMUSG00000009596<br>ENSMUSG00000026915<br>ENSMUSG00000041791<br>ENSMUSG00000090083<br>ENSMUSG00000049470<br>ENSMUSG00000068117<br>ENSMUSG00000018554<br>ENSMUSG00000027719<br>ENSMUSG00000060985<br>ENSMUSG00000006435	

ENSMUSG00000010461	ENSMUSG00000020308	
ENSMUSG00000030838	ENSMUSG00000031755	
ENSMUSG00000009487	ENSMUSG00000019861	
ENSMUSG00000024330	ENSMUSG00000011349	
ENSMUSG00000007653	ENSMUSG00000048077	
ENSMUSG00000022141	ENSMUSG00000047654	
ENSMUSG00000005360	ENSMUSG00000040850	
ENSMUSG00000049555	ENSMUSG00000050799	
ENSMUSG00000033676	ENSMUSG00000066687	
ENSMUSG00000042367	ENSMUSG00000013089	
ENSMUSG00000002799	ENSMUSG00000047014	
ENSMUSG00000027254	ENSMUSG00000041301	
ENSMUSG00000025232	ENSMUSG00000024430	
ENSMUSG00000021665	ENSMUSG00000030669	
ENSMUSG00000056854	ENSMUSG00000005469	
ENSMUSG00000023959	ENSMUSG00000020664	
ENSMUSG00000033498	ENSMUSG00000001225	
ENSMUSG00000028943	ENSMUSG00000020131	
ENSMUSG00000062252	ENSMUSG00000040132	
ENSMUSG00000003031	ENSMUSG00000074378	
ENSMUSG00000020953	ENSMUSG00000095063	
ENSMUSG00000046352	ENSMUSG00000027378	
ENSMUSG00000040055	ENSMUSG00000020096	
ENSMUSG00000000303	ENSMUSG00000045179	
ENSMUSG00000049791	ENSMUSG00000026142	
ENSMUSG00000005103		
ENSMUSG00000009097		
ENSMUSG00000030302		
ENSMUSG00000021636		
ENSMUSG00000074637		
ENSMUSG00000048707		
ENSMUSG00000044055		
ENSMUSG00000038248		
ENSMUSG00000056966		
ENSMUSG00000032419		
ENSMUSG00000096051		
ENSMUSG00000094586		
ENSMUSG00000068232		
ENSMUSG00000068231		
ENSMUSG00000068234		
ENSMUSG00000061653		
ENSMUSG00000060724		
ENSMUSG00000057592		
ENSMUSG00000095932		
ENSMUSG00000094553		
ENSMUSG00000060816		
ENSMUSG00000062546		
ENSMUSG00000057697		
ENSMUSG00000047203		
ENSMUSG00000093635		
ENSMUSG00000064259		
ENSMUSG00000093692		
ENSMUSG00000058731		
ENSMUSG00000094206		
ENSMUSG00000091734		
ENSMUSG00000095670		
ENSMUSG00000027523		
ENSMUSG00000069708		
ENSMUSG00000069707		
ENSMUSG00000069706		
ENSMUSG00000093811		
ENSMUSG00000043314		
ENSMUSG00000050813		
ENSMUSG00000058807		
ENSMUSG00000050818		
ENSMUSG00000057654		
ENSMUSG00000059279		
ENSMUSG00000060765		
ENSMUSG00000056564		
ENSMUSG00000043880		
ENSMUSG00000063549		
ENSMUSG00000061344		
ENSMUSG00000064044		
ENSMUSG00000058222		
ENSMUSG00000060030		
ENSMUSG00000061835		
ENSMUSG00000056959		

ENSMUSG00000059504<br>ENSMUSG00000070438<br>ENSMUSG00000096806<br>ENSMUSG00000094805<br>ENSMUSG00000061165<br>ENSMUSG00000063350<br>ENSMUSG00000058628<br>ENSMUSG00000066750<br>ENSMUSG00000066749<br>ENSMUSG00000066748<br>ENSMUSG00000066747<br>ENSMUSG00000060583<br>ENSMUSG00000094461<br>ENSMUSG00000095893<br>ENSMUSG00000096424<br>ENSMUSG00000096167<br>ENSMUSG00000095527<br>ENSMUSG00000096356<br>ENSMUSG00000096409<br>ENSMUSG00000045528<br>ENSMUSG00000093901<br>ENSMUSG00000070311<br>ENSMUSG00000094778<br>ENSMUSG00000096427<br>ENSMUSG00000058270<br>ENSMUSG00000094588<br>ENSMUSG00000049864<br>ENSMUSG00000096757<br>ENSMUSG00000049098<br>ENSMUSG00000052058<br>ENSMUSG00000089717<br>ENSMUSG00000049334<br>ENSMUSG00000094380<br>ENSMUSG00000096794<br>ENSMUSG00000039962<br>ENSMUSG00000094810<br>ENSMUSG00000057444<br>ENSMUSG00000060114<br>ENSMUSG00000059189<br>ENSMUSG00000047050<br>ENSMUSG00000094701<br>ENSMUSG00000049648<br>ENSMUSG00000063225<br>ENSMUSG00000046150<br>ENSMUSG00000022762<br>ENSMUSG00000051593<br>ENSMUSG00000056961<br>ENSMUSG00000070983<br>ENSMUSG00000061039<br>ENSMUSG00000049926<br>ENSMUSG00000043911<br>ENSMUSG00000044798<br>ENSMUSG00000062103<br>ENSMUSG00000047667<br>ENSMUSG00000064333<br>ENSMUSG00000063221<br>ENSMUSG00000058515<br>ENSMUSG00000057424<br>ENSMUSG00000059595<br>ENSMUSG00000058820<br>ENSMUSG00000095194<br>ENSMUSG00000094182<br>ENSMUSG00000048501<br>ENSMUSG00000049708<br>ENSMUSG00000094970<br>ENSMUSG00000096555<br>ENSMUSG00000063380<br>ENSMUSG00000057349<br>ENSMUSG00000094269<br>ENSMUSG00000058856<br>ENSMUSG00000044814<br>ENSMUSG00000043925<br>ENSMUSG00000044824<br>ENSMUSG00000048292<br>ENSMUSG00000073979<br>ENSMUSG00000060556<br>ENSMUSG00000067545		

ENSMUSG00000073977<br>ENSMUSG00000060878<br>ENSMUSG00000073975<br>ENSMUSG00000073974<br>ENSMUSG00000073973<br>ENSMUSG00000094745<br>ENSMUSG00000073972<br>ENSMUSG00000073971<br>ENSMUSG00000063176<br>ENSMUSG00000073970<br>ENSMUSG00000073969<br>ENSMUSG00000062649<br>ENSMUSG00000095322<br>ENSMUSG00000073967<br>ENSMUSG00000070423<br>ENSMUSG00000050853<br>ENSMUSG00000066273<br>ENSMUSG00000059366<br>ENSMUSG00000066272<br>ENSMUSG00000043366<br>ENSMUSG00000073966<br>ENSMUSG00000048469<br>ENSMUSG00000060888<br>ENSMUSG00000073965<br>ENSMUSG00000062142<br>ENSMUSG00000073964<br>ENSMUSG00000043310<br>ENSMUSG00000073963<br>ENSMUSG00000045824<br>ENSMUSG00000066269<br>ENSMUSG00000073962<br>ENSMUSG00000043354<br>ENSMUSG00000045792<br>ENSMUSG00000073961<br>ENSMUSG00000073960<br>ENSMUSG00000067529<br>ENSMUSG00000073959<br>ENSMUSG00000067528<br>ENSMUSG00000078080<br>ENSMUSG00000067526<br>ENSMUSG00000066268<br>ENSMUSG00000051362<br>ENSMUSG00000044994<br>ENSMUSG00000057461<br>ENSMUSG00000048299<br>ENSMUSG00000073956<br>ENSMUSG00000059867<br>ENSMUSG00000067525<br>ENSMUSG00000073955<br>ENSMUSG00000067524<br>ENSMUSG00000073954<br>ENSMUSG00000067522<br>ENSMUSG00000059106<br>ENSMUSG00000094133<br>ENSMUSG00000067519<br>ENSMUSG00000064110<br>ENSMUSG00000060049<br>ENSMUSG00000062121<br>ENSMUSG00000073952<br>ENSMUSG00000073951<br>ENSMUSG00000095839<br>ENSMUSG00000073950<br>ENSMUSG00000045540<br>ENSMUSG00000094353<br>ENSMUSG00000067513<br>ENSMUSG00000061200<br>ENSMUSG00000096436<br>ENSMUSG00000059874<br>ENSMUSG00000055820<br>ENSMUSG00000046650<br>ENSMUSG00000050815<br>ENSMUSG00000095903<br>ENSMUSG00000094254<br>ENSMUSG00000058079<br>ENSMUSG00000073949<br>ENSMUSG00000095774<br>ENSMUSG00000042402		

ENSMUSG00000073948<br>ENSMUSG00000093934<br>ENSMUSG00000046396<br>ENSMUSG00000094449<br>ENSMUSG00000045584<br>ENSMUSG00000096584<br>ENSMUSG00000095390<br>ENSMUSG00000094119<br>ENSMUSG00000051493<br>ENSMUSG00000078624<br>ENSMUSG00000073947<br>ENSMUSG00000043331<br>ENSMUSG00000047544<br>ENSMUSG00000073946<br>ENSMUSG00000047352<br>ENSMUSG00000073945<br>ENSMUSG00000044292<br>ENSMUSG00000073944<br>ENSMUSG00000059473<br>ENSMUSG00000045132<br>ENSMUSG00000060254<br>ENSMUSG00000050085<br>ENSMUSG00000055124<br>ENSMUSG00000046678<br>ENSMUSG00000045780<br>ENSMUSG00000049010<br>ENSMUSG00000094822<br>ENSMUSG00000044205<br>ENSMUSG00000096516<br>ENSMUSG00000047545<br>ENSMUSG00000045812<br>ENSMUSG00000050281<br>ENSMUSG00000049073<br>ENSMUSG00000058662<br>ENSMUSG00000061626<br>ENSMUSG00000051095<br>ENSMUSG00000047535<br>ENSMUSG00000058200<br>ENSMUSG00000063615<br>ENSMUSG00000047163<br>ENSMUSG00000042219<br>ENSMUSG00000073938<br>ENSMUSG00000073937<br>ENSMUSG00000094520<br>ENSMUSG00000094063<br>ENSMUSG00000066263<br>ENSMUSG00000066262<br>ENSMUSG00000073932<br>ENSMUSG00000049797<br>ENSMUSG00000095559<br>ENSMUSG00000094302<br>ENSMUSG00000051340<br>ENSMUSG00000073931<br>ENSMUSG00000042909<br>ENSMUSG00000044899<br>ENSMUSG00000052417<br>ENSMUSG00000072707<br>ENSMUSG00000020654<br>ENSMUSG00000073928<br>ENSMUSG00000073927<br>ENSMUSG00000073926<br>ENSMUSG00000073925<br>ENSMUSG00000051182<br>ENSMUSG00000073924<br>ENSMUSG00000044441<br>ENSMUSG00000073923<br>ENSMUSG00000045030<br>ENSMUSG00000070421<br>ENSMUSG00000073922<br>ENSMUSG00000073920<br>ENSMUSG00000073917<br>ENSMUSG00000063582<br>ENSMUSG00000056782<br>ENSMUSG00000057770<br>ENSMUSG00000073916<br>ENSMUSG00000044705<br>ENSMUSG00000094531		

ENSMUSG00000051172<br>ENSMUSG00000096773<br>ENSMUSG00000073915<br>ENSMUSG00000073914<br>ENSMUSG00000073913<br>ENSMUSG00000096029<br>ENSMUSG00000095248<br>ENSMUSG00000044120<br>ENSMUSG00000047225<br>ENSMUSG00000047794<br>ENSMUSG00000048425<br>ENSMUSG00000073908<br>ENSMUSG00000073909<br>ENSMUSG00000073907<br>ENSMUSG00000050266<br>ENSMUSG00000043948<br>ENSMUSG00000073906<br>ENSMUSG00000059610<br>ENSMUSG00000048919<br>ENSMUSG00000044061<br>ENSMUSG00000030897<br>ENSMUSG00000046272<br>ENSMUSG00000045126<br>ENSMUSG00000057817<br>ENSMUSG00000060303<br>ENSMUSG00000048456<br>ENSMUSG00000049498<br>ENSMUSG00000062892<br>ENSMUSG00000046913<br>ENSMUSG00000094755<br>ENSMUSG00000094986<br>ENSMUSG00000061637<br>ENSMUSG00000062844<br>ENSMUSG00000057503<br>ENSMUSG00000045883<br>ENSMUSG00000094721<br>ENSMUSG00000096365<br>ENSMUSG00000062199<br>ENSMUSG00000096485<br>ENSMUSG00000049015<br>ENSMUSG00000063777<br>ENSMUSG00000096320<br>ENSMUSG00000095189<br>ENSMUSG00000096273<br>ENSMUSG00000096708<br>ENSMUSG00000071629<br>ENSMUSG00000095484<br>ENSMUSG00000096289<br>ENSMUSG00000094846<br>ENSMUSG00000045678<br>ENSMUSG00000061387<br>ENSMUSG00000051156<br>ENSMUSG00000050865<br>ENSMUSG00000047207<br>ENSMUSG00000048356<br>ENSMUSG00000044040<br>ENSMUSG00000045395<br>ENSMUSG00000054526<br>ENSMUSG00000057270<br>ENSMUSG00000056858<br>ENSMUSG00000059105<br>ENSMUSG00000062314<br>ENSMUSG00000051680<br>ENSMUSG00000064223<br>ENSMUSG00000057832<br>ENSMUSG00000051591<br>ENSMUSG00000059087<br>ENSMUSG00000096714<br>ENSMUSG00000094493<br>ENSMUSG00000036744<br>ENSMUSG00000056863<br>ENSMUSG00000073901<br>ENSMUSG00000073900<br>ENSMUSG00000095373<br>ENSMUSG00000096487<br>ENSMUSG00000069390<br>ENSMUSG00000045581		

ENSMUSG00000036647<br>ENSMUSG00000045013<br>ENSMUSG00000070417<br>ENSMUSG00000073898<br>ENSMUSG00000049674<br>ENSMUSG00000073897<br>ENSMUSG00000060503<br>ENSMUSG00000073896<br>ENSMUSG00000066242<br>ENSMUSG00000095910<br>ENSMUSG00000094289<br>ENSMUSG00000073893<br>ENSMUSG00000095212<br>ENSMUSG00000094197<br>ENSMUSG00000095301<br>ENSMUSG00000096151<br>ENSMUSG00000094426<br>ENSMUSG00000043855<br>ENSMUSG00000063120<br>ENSMUSG00000054236<br>ENSMUSG00000059031<br>ENSMUSG00000078118<br>ENSMUSG00000059996<br>ENSMUSG00000096068<br>ENSMUSG00000095929<br>ENSMUSG00000096465<br>ENSMUSG00000093808<br>ENSMUSG00000094612<br>ENSMUSG00000093980<br>ENSMUSG00000059227<br>ENSMUSG00000094104<br>ENSMUSG00000095239<br>ENSMUSG00000096679<br>ENSMUSG00000058014<br>ENSMUSG00000060759<br>ENSMUSG00000060105<br>ENSMUSG00000058244<br>ENSMUSG00000061000<br>ENSMUSG00000063764<br>ENSMUSG00000049280<br>ENSMUSG00000096209<br>ENSMUSG00000056946<br>ENSMUSG00000051200<br>ENSMUSG00000066241<br>ENSMUSG00000062434<br>ENSMUSG00000066240<br>ENSMUSG00000046431<br>ENSMUSG00000066239<br>ENSMUSG00000068259<br>ENSMUSG00000045514<br>ENSMUSG00000048933<br>ENSMUSG00000093825<br>ENSMUSG00000096254<br>ENSMUSG00000068437<br>ENSMUSG00000060523<br>ENSMUSG00000059488<br>ENSMUSG00000050030<br>ENSMUSG00000049011<br>ENSMUSG00000050431<br>ENSMUSG00000048080<br>ENSMUSG00000091873<br>ENSMUSG00000090874<br>ENSMUSG00000045306<br>ENSMUSG00000046210<br>ENSMUSG00000047716<br>ENSMUSG00000094692<br>ENSMUSG00000057903<br>ENSMUSG00000095917<br>ENSMUSG00000095765<br>ENSMUSG00000068431<br>ENSMUSG00000094285<br>ENSMUSG00000053815<br>ENSMUSG00000050028<br>ENSMUSG00000058188<br>ENSMUSG00000057179<br>ENSMUSG00000060084<br>ENSMUSG00000059069		

ENSMUSG00000045479<br>ENSMUSG00000049806<br>ENSMUSG00000043385<br>ENSMUSG00000068574<br>ENSMUSG00000091983<br>ENSMUSG00000090631<br>ENSMUSG00000095831<br>ENSMUSG00000095236<br>ENSMUSG00000094192<br>ENSMUSG00000054431<br>ENSMUSG00000049168<br>ENSMUSG00000043119<br>ENSMUSG00000045708<br>ENSMUSG00000073110<br>ENSMUSG00000094669<br>ENSMUSG00000071481<br>ENSMUSG00000043605<br>ENSMUSG00000048693<br>ENSMUSG00000059411<br>ENSMUSG00000061210<br>ENSMUSG00000054141<br>ENSMUSG00000078116<br>ENSMUSG00000051414<br>ENSMUSG00000062868<br>ENSMUSG00000035932<br>ENSMUSG00000095957<br>ENSMUSG00000095525<br>ENSMUSG00000045204<br>ENSMUSG00000059303<br>ENSMUSG00000048391<br>ENSMUSG00000049229<br>ENSMUSG00000061614<br>ENSMUSG00000058692<br>ENSMUSG00000059821<br>ENSMUSG00000052182<br>ENSMUSG00000094535<br>ENSMUSG00000052625<br>ENSMUSG00000051160<br>ENSMUSG00000095667<br>ENSMUSG00000043087<br>ENSMUSG00000094678<br>ENSMUSG00000058659<br>ENSMUSG00000095448<br>ENSMUSG00000066905<br>ENSMUSG00000063842<br>ENSMUSG00000051118<br>ENSMUSG00000050803<br>ENSMUSG00000044454<br>ENSMUSG00000044106<br>ENSMUSG00000058491<br>ENSMUSG00000066899<br>ENSMUSG00000061457<br>ENSMUSG00000066897<br>ENSMUSG00000059623<br>ENSMUSG00000049028<br>ENSMUSG00000066896<br>ENSMUSG00000051003<br>ENSMUSG00000059043<br>ENSMUSG00000051706<br>ENSMUSG00000069998<br>ENSMUSG00000061561<br>ENSMUSG00000046881<br>ENSMUSG00000032177<br>ENSMUSG00000021609<br>ENSMUSG00000037924<br>ENSMUSG00000062527<br>ENSMUSG00000058981<br>ENSMUSG00000046643<br>ENSMUSG00000049456<br>ENSMUSG00000044286<br>ENSMUSG00000051650<br>ENSMUSG00000075427<br>ENSMUSG00000090129<br>ENSMUSG00000045381<br>ENSMUSG00000047048<br>ENSMUSG00000050134<br>ENSMUSG00000049528		

ENSMUSG00000059371<br>ENSMUSG00000059460<br>ENSMUSG00000046486<br>ENSMUSG00000051528<br>ENSMUSG00000055033<br>ENSMUSG00000062037<br>ENSMUSG00000051793<br>ENSMUSG00000056184<br>ENSMUSG00000063780<br>ENSMUSG00000032987<br>ENSMUSG00000054036<br>ENSMUSG00000050788<br>ENSMUSG00000066672<br>ENSMUSG00000059503<br>ENSMUSG00000051509<br>ENSMUSG00000066671<br>ENSMUSG00000071185<br>ENSMUSG00000051190<br>ENSMUSG00000062873<br>ENSMUSG00000094673<br>ENSMUSG00000094080<br>ENSMUSG00000042774<br>ENSMUSG00000046493<br>ENSMUSG00000063216<br>ENSMUSG00000060205<br>ENSMUSG00000047286<br>ENSMUSG00000094891<br>ENSMUSG00000093884<br>ENSMUSG00000054666<br>ENSMUSG00000004347<br>ENSMUSG00000095030<br>ENSMUSG00000094140<br>ENSMUSG00000063867<br>ENSMUSG00000063106<br>ENSMUSG00000035626<br>ENSMUSG00000057564<br>ENSMUSG00000059887<br>ENSMUSG00000043529<br>ENSMUSG00000069430<br>ENSMUSG00000063374<br>ENSMUSG00000056853<br>ENSMUSG00000059762<br>ENSMUSG00000050198<br>ENSMUSG00000042801<br>ENSMUSG00000047868<br>ENSMUSG00000061367<br>ENSMUSG00000094944<br>ENSMUSG00000096229<br>ENSMUSG00000096220<br>ENSMUSG00000095483<br>ENSMUSG00000062914<br>ENSMUSG00000044537<br>ENSMUSG00000049573<br>ENSMUSG00000095138<br>ENSMUSG00000095075<br>ENSMUSG00000094347<br>ENSMUSG00000095696<br>ENSMUSG00000096497<br>ENSMUSG00000049217<br>ENSMUSG00000044025<br>ENSMUSG00000047626<br>ENSMUSG00000094496<br>ENSMUSG00000044293<br>ENSMUSG00000052012<br>ENSMUSG00000095002<br>ENSMUSG00000094734<br>ENSMUSG00000063452<br>ENSMUSG00000058513<br>ENSMUSG00000093866<br>ENSMUSG00000046041<br>ENSMUSG00000095401<br>ENSMUSG00000096858<br>ENSMUSG00000071065<br>ENSMUSG00000050478<br>ENSMUSG00000049894<br>ENSMUSG00000050251<br>ENSMUSG00000069421		

ENSMUSG00000063173<br>ENSMUSG00000049052<br>ENSMUSG00000052818<br>ENSMUSG00000059134<br>ENSMUSG00000061961<br>ENSMUSG00000063715<br>ENSMUSG00000058071<br>ENSMUSG00000094295<br>ENSMUSG00000048745<br>ENSMUSG00000044897<br>ENSMUSG00000058251<br>ENSMUSG00000096747<br>ENSMUSG00000095804<br>ENSMUSG00000058084<br>ENSMUSG00000059862<br>ENSMUSG00000045559<br>ENSMUSG00000048153<br>ENSMUSG00000024524<br>ENSMUSG00000052819<br>ENSMUSG00000051952<br>ENSMUSG00000043698<br>ENSMUSG00000043383<br>ENSMUSG00000046790<br>ENSMUSG00000070821<br>ENSMUSG00000070820<br>ENSMUSG00000095218<br>ENSMUSG00000061075<br>ENSMUSG00000073770<br>ENSMUSG00000073769<br>ENSMUSG00000073768<br>ENSMUSG00000096705<br>ENSMUSG00000096368<br>ENSMUSG00000074966<br>ENSMUSG00000063844<br>ENSMUSG00000074965<br>ENSMUSG00000068647<br>ENSMUSG00000068646<br>ENSMUSG00000068645<br>ENSMUSG00000095156<br>ENSMUSG00000096554<br>ENSMUSG00000064266<br>ENSMUSG00000050781<br>ENSMUSG00000096703<br>ENSMUSG00000095586<br>ENSMUSG00000044039<br>ENSMUSG00000061195<br>ENSMUSG00000095809<br>ENSMUSG00000094000<br>ENSMUSG00000074959<br>ENSMUSG00000094858<br>ENSMUSG00000096853<br>ENSMUSG00000074957<br>ENSMUSG00000057149<br>ENSMUSG00000044560<br>ENSMUSG00000093804<br>ENSMUSG00000074955<br>ENSMUSG00000096566<br>ENSMUSG00000094747<br>ENSMUSG00000074952<br>ENSMUSG00000051180<br>ENSMUSG00000051051<br>ENSMUSG00000050366<br>ENSMUSG00000074951<br>ENSMUSG00000074949<br>ENSMUSG00000074948<br>ENSMUSG00000074947<br>ENSMUSG00000074946<br>ENSMUSG00000074945<br>ENSMUSG00000061489<br>ENSMUSG00000074943<br>ENSMUSG00000062782<br>ENSMUSG00000060112<br>ENSMUSG00000060974<br>ENSMUSG00000050776<br>ENSMUSG00000062712<br>ENSMUSG00000063823<br>ENSMUSG00000049758		

ENSMUSG00000056883<br>ENSMUSG00000063230<br>ENSMUSG00000050215<br>ENSMUSG00000052508<br>ENSMUSG00000046450<br>ENSMUSG00000095901<br>ENSMUSG00000093942<br>ENSMUSG00000096069<br>ENSMUSG00000094819<br>ENSMUSG00000059136<br>ENSMUSG00000066122<br>ENSMUSG00000057997<br>ENSMUSG00000044801<br>ENSMUSG00000059448<br>ENSMUSG00000059101<br>ENSMUSG00000071000<br>ENSMUSG00000071764<br>ENSMUSG00000067971<br>ENSMUSG00000048101<br>ENSMUSG00000056281<br>ENSMUSG00000050504<br>ENSMUSG00000048173<br>ENSMUSG00000006464<br>ENSMUSG00000044308<br>ENSMUSG00000093839<br>ENSMUSG00000093920<br>ENSMUSG00000060787<br>ENSMUSG00000075223<br>ENSMUSG00000075222<br>ENSMUSG00000075221<br>ENSMUSG00000075220<br>ENSMUSG00000075219<br>ENSMUSG00000075218<br>ENSMUSG00000059990<br>ENSMUSG00000062128<br>ENSMUSG00000046076<br>ENSMUSG00000075215<br>ENSMUSG00000075214<br>ENSMUSG00000075212<br>ENSMUSG00000063881<br>ENSMUSG00000075211<br>ENSMUSG00000050603<br>ENSMUSG00000043226<br>ENSMUSG00000075210<br>ENSMUSG00000069823<br>ENSMUSG00000053287<br>ENSMUSG00000059379<br>ENSMUSG00000033850<br>ENSMUSG00000075209<br>ENSMUSG00000043892<br>ENSMUSG00000075208<br>ENSMUSG00000046975<br>ENSMUSG00000057761<br>ENSMUSG00000050128<br>ENSMUSG00000055971<br>ENSMUSG00000075206<br>ENSMUSG00000021013<br>ENSMUSG00000072709<br>ENSMUSG00000042863<br>ENSMUSG00000095312<br>ENSMUSG00000070383<br>ENSMUSG00000059873<br>ENSMUSG00000044923<br>ENSMUSG00000043267<br>ENSMUSG00000042796<br>ENSMUSG00000069818<br>ENSMUSG00000045392<br>ENSMUSG00000060422<br>ENSMUSG00000061984<br>ENSMUSG00000061704<br>ENSMUSG00000075205<br>ENSMUSG00000094488<br>ENSMUSG00000075204<br>ENSMUSG00000075203<br>ENSMUSG00000075202<br>ENSMUSG00000056921<br>ENSMUSG00000075201		

ENSMUSG00000062186<br>ENSMUSG00000075200<br>ENSMUSG00000075199<br>ENSMUSG00000069816<br>ENSMUSG00000075198<br>ENSMUSG00000075197<br>ENSMUSG00000075196<br>ENSMUSG00000057050<br>ENSMUSG00000075195<br>ENSMUSG00000075194<br>ENSMUSG00000049041<br>ENSMUSG00000075193<br>ENSMUSG00000047149<br>ENSMUSG00000047444<br>ENSMUSG00000043692<br>ENSMUSG00000070380<br>ENSMUSG00000070379<br>ENSMUSG00000070378<br>ENSMUSG00000075190<br>ENSMUSG00000070377<br>ENSMUSG00000070374<br>ENSMUSG00000075189<br>ENSMUSG00000075188<br>ENSMUSG00000075187<br>ENSMUSG00000063116<br>ENSMUSG00000075186<br>ENSMUSG00000075185<br>ENSMUSG00000090059<br>ENSMUSG00000054406<br>ENSMUSG00000075182<br>ENSMUSG00000075181<br>ENSMUSG00000075180<br>ENSMUSG00000060742<br>ENSMUSG00000058275<br>ENSMUSG00000075179<br>ENSMUSG00000075178<br>ENSMUSG00000079239<br>ENSMUSG00000075177<br>ENSMUSG00000075176<br>ENSMUSG00000075175<br>ENSMUSG00000075174<br>ENSMUSG00000075173<br>ENSMUSG00000031789<br>ENSMUSG00000075172<br>ENSMUSG00000047969<br>ENSMUSG00000078420<br>ENSMUSG00000044213<br>ENSMUSG00000075171<br>ENSMUSG00000075170<br>ENSMUSG00000075169<br>ENSMUSG00000075168<br>ENSMUSG00000070875<br>ENSMUSG00000075167<br>ENSMUSG00000049843<br>ENSMUSG00000075166<br>ENSMUSG00000075165<br>ENSMUSG00000075164<br>ENSMUSG00000075163<br>ENSMUSG00000075161<br>ENSMUSG00000075160<br>ENSMUSG00000075159<br>ENSMUSG00000075158<br>ENSMUSG00000044487<br>ENSMUSG00000070857<br>ENSMUSG00000070856<br>ENSMUSG00000089732<br>ENSMUSG00000070853<br>ENSMUSG00000070852<br>ENSMUSG00000047594<br>ENSMUSG00000043274<br>ENSMUSG00000050772<br>ENSMUSG00000058194<br>ENSMUSG00000061520<br>ENSMUSG00000075156<br>ENSMUSG00000062272<br>ENSMUSG00000059205<br>ENSMUSG00000068819		

ENSMUSG00000068818<br>ENSMUSG00000075155<br>ENSMUSG00000075154<br>ENSMUSG00000075153<br>ENSMUSG00000075151<br>ENSMUSG00000075150<br>ENSMUSG00000075149<br>ENSMUSG00000068817<br>ENSMUSG00000075148<br>ENSMUSG00000068816<br>ENSMUSG00000068815<br>ENSMUSG00000068814<br>ENSMUSG00000061875<br>ENSMUSG00000047039<br>ENSMUSG00000045225<br>ENSMUSG00000048197<br>ENSMUSG00000075146<br>ENSMUSG00000075145<br>ENSMUSG00000075144<br>ENSMUSG00000075143<br>ENSMUSG00000075142<br>ENSMUSG00000062793<br>ENSMUSG00000075141<br>ENSMUSG00000045150<br>ENSMUSG00000075140<br>ENSMUSG00000075139<br>ENSMUSG00000075137<br>ENSMUSG00000075136<br>ENSMUSG00000075135<br>ENSMUSG00000075134<br>ENSMUSG00000061342<br>ENSMUSG00000075133<br>ENSMUSG00000075132<br>ENSMUSG00000050023<br>ENSMUSG00000056995<br>ENSMUSG00000075127<br>ENSMUSG00000075126<br>ENSMUSG00000075125<br>ENSMUSG00000049372<br>ENSMUSG00000051424<br>ENSMUSG00000057735<br>ENSMUSG00000068809<br>ENSMUSG00000068808<br>ENSMUSG00000060827<br>ENSMUSG00000075121<br>ENSMUSG00000075120<br>ENSMUSG00000075119<br>ENSMUSG00000075117<br>ENSMUSG00000089892<br>ENSMUSG00000075115<br>ENSMUSG00000059023<br>ENSMUSG00000046623<br>ENSMUSG00000064084<br>ENSMUSG00000057447<br>ENSMUSG00000061798<br>ENSMUSG00000062757<br>ENSMUSG00000075114<br>ENSMUSG00000075113<br>ENSMUSG00000075112<br>ENSMUSG00000048226<br>ENSMUSG00000089751<br>ENSMUSG00000075110<br>ENSMUSG00000075108<br>ENSMUSG00000075107<br>ENSMUSG00000075106<br>ENSMUSG00000075105<br>ENSMUSG00000075104<br>ENSMUSG00000075103<br>ENSMUSG00000075102<br>ENSMUSG00000075101<br>ENSMUSG00000075100<br>ENSMUSG00000075098<br>ENSMUSG00000075097<br>ENSMUSG00000075096<br>ENSMUSG00000050288<br>ENSMUSG00000075095<br>ENSMUSG00000075094		

ENSMUSG00000075093<br>ENSMUSG00000075092<br>ENSMUSG00000090824<br>ENSMUSG00000048620<br>ENSMUSG00000054938<br>ENSMUSG00000096228<br>ENSMUSG00000034583<br>ENSMUSG00000093877<br>ENSMUSG00000048067<br>ENSMUSG00000075091<br>ENSMUSG00000056696<br>ENSMUSG00000045148<br>ENSMUSG00000056600<br>ENSMUSG00000095377<br>ENSMUSG00000096477<br>ENSMUSG00000091601<br>ENSMUSG00000043827<br>ENSMUSG00000049561<br>ENSMUSG00000064121<br>ENSMUSG00000058802<br>ENSMUSG00000063660<br>ENSMUSG00000075072<br>ENSMUSG00000049057<br>ENSMUSG00000061972<br>ENSMUSG00000092077<br>ENSMUSG00000091531<br>ENSMUSG00000049618<br>ENSMUSG00000063188<br>ENSMUSG00000059687<br>ENSMUSG00000029184<br>ENSMUSG00000049149<br>ENSMUSG00000068806<br>ENSMUSG00000090894<br>ENSMUSG00000042894<br>ENSMUSG00000090675<br>ENSMUSG00000061295<br>ENSMUSG00000051611<br>ENSMUSG00000051313<br>ENSMUSG00000092292<br>ENSMUSG00000062629<br>ENSMUSG00000092413<br>ENSMUSG00000059112<br>ENSMUSG00000075069<br>ENSMUSG00000059910<br>ENSMUSG00000061336<br>ENSMUSG00000075068<br>ENSMUSG00000095286<br>ENSMUSG00000075067<br>ENSMUSG00000080990<br>ENSMUSG00000075066<br>ENSMUSG00000059964<br>ENSMUSG00000075065<br>ENSMUSG00000063994<br>ENSMUSG00000075063<br>ENSMUSG00000060017<br>ENSMUSG00000075062<br>ENSMUSG00000083947<br>ENSMUSG00000045202<br>ENSMUSG00000044985<br>ENSMUSG00000075061<br>ENSMUSG00000050613<br>ENSMUSG00000061777<br>ENSMUSG00000058114<br>ENSMUSG00000059030<br>ENSMUSG00000071084<br>ENSMUSG00000081724<br>ENSMUSG00000094878<br>ENSMUSG00000043312<br>ENSMUSG00000067186<br>ENSMUSG00000063240<br>ENSMUSG00000096009<br>ENSMUSG00000057801<br>ENSMUSG00000057443<br>ENSMUSG00000054940<br>ENSMUSG00000096840<br>ENSMUSG00000040328<br>ENSMUSG00000047511		

ENSMUSG00000050763<br>ENSMUSG00000048378<br>ENSMUSG00000025235<br>ENSMUSG00000059864<br>ENSMUSG00000050959<br>ENSMUSG00000057168<br>ENSMUSG00000096909<br>ENSMUSG00000045421<br>ENSMUSG00000046575<br>ENSMUSG00000047702<br>ENSMUSG00000063386<br>ENSMUSG00000062285<br>ENSMUSG00000059729<br>ENSMUSG00000044170<br>ENSMUSG00000057767<br>ENSMUSG00000063827<br>ENSMUSG00000095187<br>ENSMUSG00000091308<br>ENSMUSG00000005864<br>ENSMUSG00000070460<br>ENSMUSG00000070459<br>ENSMUSG00000057540<br>ENSMUSG00000054054<br>ENSMUSG00000054498<br>ENSMUSG00000055610<br>ENSMUSG00000055571<br>ENSMUSG00000062426<br>ENSMUSG00000039608<br>ENSMUSG00000061549<br>ENSMUSG00000020168<br>ENSMUSG00000062878<br>ENSMUSG00000057067<br>ENSMUSG00000059319<br>ENSMUSG00000062042<br>ENSMUSG00000063394<br>ENSMUSG00000060688<br>ENSMUSG00000027274<br>ENSMUSG00000029268<br>ENSMUSG00000050343<br>ENSMUSG00000061952<br>ENSMUSG00000060918<br>ENSMUSG00000059397<br>ENSMUSG00000050742<br>ENSMUSG00000050158<br>ENSMUSG00000056822<br>ENSMUSG00000045341<br>ENSMUSG00000061361<br>ENSMUSG00000068535<br>ENSMUSG00000062245<br>ENSMUSG00000060480<br>ENSMUSG00000074006<br>ENSMUSG00000073998<br>ENSMUSG00000073997<br>ENSMUSG00000068950<br>ENSMUSG00000094464<br>ENSMUSG00000094266<br>ENSMUSG00000075387<br>ENSMUSG00000061305<br>ENSMUSG00000096822<br>ENSMUSG00000059251<br>ENSMUSG00000094764<br>ENSMUSG00000057351<br>ENSMUSG00000049315<br>ENSMUSG00000094949<br>ENSMUSG00000075384<br>ENSMUSG00000050015<br>ENSMUSG00000075383<br>ENSMUSG00000053146<br>ENSMUSG00000075382<br>ENSMUSG00000055088<br>ENSMUSG00000075380<br>ENSMUSG00000070943<br>ENSMUSG00000055838<br>ENSMUSG00000075379<br>ENSMUSG00000083361<br>ENSMUSG00000075378<br>ENSMUSG00000075377		

ENSMUSG00000059429<br>ENSMUSG00000068947<br>ENSMUSG00000049018<br>ENSMUSG00000062204<br>ENSMUSG00000060170<br>ENSMUSG00000042869<br>ENSMUSG00000045474<br>ENSMUSG00000045508<br>ENSMUSG00000048996<br>ENSMUSG00000054890<br>ENSMUSG00000046016<br>ENSMUSG00000051258<br>ENSMUSG00000036658<br>ENSMUSG00000049737<br>ENSMUSG00000094398<br>ENSMUSG00000096404<br>ENSMUSG00000053391<br>ENSMUSG00000053251<br>ENSMUSG00000081649<br>ENSMUSG00000051046<br>ENSMUSG00000050654<br>ENSMUSG00000071510<br>ENSMUSG00000049362<br>ENSMUSG00000063137<br>ENSMUSG00000044029<br>ENSMUSG00000090629<br>ENSMUSG00000090951<br>ENSMUSG00000062105<br>ENSMUSG00000047960<br>ENSMUSG00000043357<br>ENSMUSG00000064006<br>ENSMUSG00000094539<br>ENSMUSG00000060057<br>ENSMUSG00000063020<br>ENSMUSG00000062608<br>ENSMUSG00000096695<br>ENSMUSG00000052537<br>ENSMUSG00000074996<br>ENSMUSG00000074995<br>ENSMUSG00000048810<br>ENSMUSG00000068182<br>ENSMUSG00000095928<br>ENSMUSG00000094422<br>ENSMUSG00000066257<br>ENSMUSG00000095706<br>ENSMUSG00000067064<br>ENSMUSG00000057464<br>ENSMUSG00000042849<br>ENSMUSG00000058904<br>ENSMUSG00000046300<br>ENSMUSG00000062497<br>ENSMUSG00000063583<br>ENSMUSG00000061616<br>ENSMUSG00000051392<br>ENSMUSG00000023030<br>ENSMUSG00000019969<br>ENSMUSG00000021448<br>ENSMUSG00000037868<br>ENSMUSG00000019772<br>ENSMUSG00000038676<br>ENSMUSG00000038418<br>ENSMUSG00000044288<br>ENSMUSG00000028222<br>ENSMUSG00000010609<br>ENSMUSG00000000552<br>ENSMUSG00000005142<br>ENSMUSG00000005583<br>ENSMUSG00000048482<br>ENSMUSG00000028072<br>ENSMUSG00000027827<br>ENSMUSG00000002900<br>ENSMUSG00000026782<br>ENSMUSG00000037251<br>ENSMUSG00000031751<br>ENSMUSG00000033208<br>ENSMUSG00000029104<br>ENSMUSG00000079037		

ENSMUSG00000096225<br>ENSMUSG00000031189<br>ENSMUSG00000049551<br>ENSMUSG00000000594<br>ENSMUSG00000058756<br>ENSMUSG00000025855<br>ENSMUSG00000028747<br>ENSMUSG00000022602<br>ENSMUSG00000025318<br>ENSMUSG00000022973<br>ENSMUSG00000039419<br>ENSMUSG00000036678<br>ENSMUSG00000026080<br>ENSMUSG00000022623<br>ENSMUSG00000052087<br>ENSMUSG00000013662<br>ENSMUSG00000022208<br>ENSMUSG00000055254<br>ENSMUSG00000002997<br>ENSMUSG00000024912<br>ENSMUSG00000037996<br>ENSMUSG00000031137<br>ENSMUSG00000032327<br>ENSMUSG00000052684<br>ENSMUSG00000029838<br>ENSMUSG00000022240<br>ENSMUSG00000000326<br>ENSMUSG00000030310<br>ENSMUSG00000021314<br>ENSMUSG00000028664<br>ENSMUSG00000053024<br>ENSMUSG00000032487<br>ENSMUSG00000098892<br>ENSMUSG00000020838<br>ENSMUSG00000040907<br>ENSMUSG00000027859<br>ENSMUSG00000034987<br>ENSMUSG00000026768<br>ENSMUSG00000046949<br>ENSMUSG00000035283<br>ENSMUSG00000000555<br>ENSMUSG00000064023<br>ENSMUSG00000027301<br>ENSMUSG00000022681<br>ENSMUSG00000050321<br>ENSMUSG00000023017<br>ENSMUSG00000000889<br>ENSMUSG00000034997<br>ENSMUSG00000026981<br>ENSMUSG00000042644<br>ENSMUSG00000048583<br>ENSMUSG00000049112<br>ENSMUSG00000042632<br>ENSMUSG00000051177<br>ENSMUSG00000018634<br>ENSMUSG00000053004<br>ENSMUSG00000001507<br>ENSMUSG00000014351<br>ENSMUSG00000021701<br>ENSMUSG00000024742<br>ENSMUSG00000027239<br>ENSMUSG00000000753<br>ENSMUSG00000062202<br>ENSMUSG00000015605<br>ENSMUSG00000020431<br>ENSMUSG00000042453<br>ENSMUSG00000038738<br>ENSMUSG00000020516<br>ENSMUSG00000074121<br>ENSMUSG00000024083<br>ENSMUSG00000005949<br>ENSMUSG00000043110<br>ENSMUSG00000021794<br>ENSMUSG00000029053<br>ENSMUSG00000022376<br>ENSMUSG00000015829<br>ENSMUSG00000030720		

ENSMUSG00000039358<br>ENSMUSG00000038255<br>ENSMUSG00000020423<br>ENSMUSG00000078672<br>ENSMUSG00000062078<br>ENSMUSG00000044407<br>ENSMUSG00000002250<br>ENSMUSG00000037625<br>ENSMUSG00000031425<br>ENSMUSG00000022892<br>ENSMUSG00000037032<br>ENSMUSG00000027852<br>ENSMUSG00000022039<br>ENSMUSG00000021109<br>ENSMUSG00000030265<br>ENSMUSG00000020716<br>ENSMUSG00000035168<br>ENSMUSG00000073804<br>ENSMUSG00000046876<br>ENSMUSG00000025485<br>ENSMUSG00000025499<br>ENSMUSG00000005672<br>ENSMUSG00000038580<br>ENSMUSG00000067629<br>ENSMUSG00000021670<br>ENSMUSG00000061718<br>ENSMUSG00000059246<br>ENSMUSG00000004110<br>ENSMUSG00000057280<br>ENSMUSG00000034460<br>ENSMUSG00000030409<br>ENSMUSG00000020182<br>ENSMUSG00000021071<br>ENSMUSG00000025867<br>ENSMUSG00000033615<br>ENSMUSG00000052301<br>ENSMUSG00000030805<br>ENSMUSG00000029468<br>ENSMUSG00000019998<br>ENSMUSG00000039714<br>ENSMUSG00000020894<br>ENSMUSG00000028879<br>ENSMUSG00000061601<br>ENSMUSG00000022568<br>ENSMUSG00000034799<br>ENSMUSG00000007207<br>ENSMUSG00000054423<br>ENSMUSG00000017978<br>ENSMUSG00000020886<br>ENSMUSG00000033906<br>ENSMUSG00000074939<br>ENSMUSG00000020723<br>ENSMUSG00000042372<br>ENSMUSG00000031990<br>ENSMUSG00000019146<br>ENSMUSG00000045092<br>ENSMUSG00000016346<br>ENSMUSG00000026442<br>ENSMUSG00000017412<br>ENSMUSG00000020635<br>ENSMUSG00000029862<br>ENSMUSG00000019194<br>ENSMUSG00000064329<br>ENSMUSG00000066058<br>ENSMUSG00000020598<br>ENSMUSG00000069601<br>ENSMUSG00000029470<br>ENSMUSG00000075316<br>ENSMUSG00000020787<br>ENSMUSG00000054934<br>ENSMUSG00000061576<br>ENSMUSG00000068696<br>ENSMUSG00000022416<br>ENSMUSG00000037610<br>ENSMUSG00000091091<br>ENSMUSG00000053647<br>ENSMUSG00000059363		

ENSMUSG00000067724<br>ENSMUSG00000003974<br>ENSMUSG00000036437<br>ENSMUSG00000041681<br>ENSMUSG00000049281<br>ENSMUSG00000042448<br>ENSMUSG00000034285<br>ENSMUSG00000024256<br>ENSMUSG00000022122<br>ENSMUSG00000079056<br>ENSMUSG00000000766<br>ENSMUSG00000074037<br>ENSMUSG00000031616<br>ENSMUSG00000019828<br>ENSMUSG00000004655<br>ENSMUSG00000063065<br>ENSMUSG00000029223<br>ENSMUSG00000041347<br>ENSMUSG00000027765<br>ENSMUSG00000027820<br>ENSMUSG00000021367<br>ENSMUSG00000063358<br>ENSMUSG00000029236<br>ENSMUSG00000005952<br>ENSMUSG00000004296<br>ENSMUSG00000033585<br>ENSMUSG00000022270<br>ENSMUSG00000020892<br>ENSMUSG00000049103<br>ENSMUSG00000062585<br>ENSMUSG00000056648<br>ENSMUSG00000049612<br>ENSMUSG00000054252<br>ENSMUSG00000041309<br>ENSMUSG00000021379<br>ENSMUSG00000020774<br>ENSMUSG00000048001<br>ENSMUSG00000095139<br>ENSMUSG00000030890<br>ENSMUSG00000071176<br>ENSMUSG00000036560<br>ENSMUSG00000045629<br>ENSMUSG00000039952<br>ENSMUSG00000090125<br>ENSMUSG00000019899<br>ENSMUSG00000040537<br>ENSMUSG00000029050<br>ENSMUSG00000031985<br>ENSMUSG00000017167<br>ENSMUSG00000030342<br>ENSMUSG00000024044<br>ENSMUSG00000032854<br>ENSMUSG00000032826<br>ENSMUSG00000033579<br>ENSMUSG00000004056<br>ENSMUSG00000021112<br>ENSMUSG00000001729<br>ENSMUSG00000005125<br>ENSMUSG00000031918<br>ENSMUSG00000018217<br>ENSMUSG00000025949<br>ENSMUSG00000038417<br>ENSMUSG00000034220<br>ENSMUSG00000030400<br>ENSMUSG00000037025<br>ENSMUSG00000027995<br>ENSMUSG00000048078<br>ENSMUSG00000043924<br>ENSMUSG00000020081<br>ENSMUSG00000022935<br>ENSMUSG00000027438<br>ENSMUSG00000031538<br>ENSMUSG00000071424<br>ENSMUSG00000026514<br>ENSMUSG00000026024<br>ENSMUSG00000024873<br>ENSMUSG00000006024		

ENSMUSG00000021557<br>ENSMUSG00000035936<br>ENSMUSG00000043943<br>ENSMUSG00000057880<br>ENSMUSG00000057606<br>ENSMUSG00000025037<br>ENSMUSG00000026687<br>ENSMUSG00000026787<br>ENSMUSG00000070880<br>ENSMUSG00000035735<br>ENSMUSG00000039206<br>ENSMUSG00000029563<br>ENSMUSG00000049721<br>ENSMUSG00000025402<br>ENSMUSG00000026812<br>ENSMUSG00000002603<br>ENSMUSG00000038668<br>ENSMUSG00000051469<br>ENSMUSG00000039830<br>ENSMUSG00000027375<br>ENSMUSG00000029145<br>ENSMUSG00000004788<br>ENSMUSG00000023000<br>ENSMUSG00000024527<br>ENSMUSG00000002881<br>ENSMUSG00000050947<br>ENSMUSG00000027312<br>ENSMUSG00000028402<br>ENSMUSG00000041607<br>ENSMUSG00000021703<br>ENSMUSG00000031775<br>ENSMUSG00000041378<br>ENSMUSG00000019843<br>ENSMUSG00000003235<br>ENSMUSG00000062312<br>ENSMUSG00000045382<br>ENSMUSG00000032112<br>ENSMUSG00000045103<br>ENSMUSG00000015994<br>ENSMUSG00000062991<br>ENSMUSG00000022619<br>ENSMUSG00000055493<br>ENSMUSG00000031214<br>ENSMUSG00000015488<br>ENSMUSG00000028488<br>ENSMUSG00000040276<br>ENSMUSG00000020368<br>ENSMUSG00000035392<br>ENSMUSG00000022048<br>ENSMUSG00000030849<br>ENSMUSG00000057667<br>ENSMUSG00000021824<br>ENSMUSG00000024480<br>ENSMUSG00000031539<br>ENSMUSG00000090247<br>ENSMUSG00000038982<br>ENSMUSG00000057531<br>ENSMUSG00000021686<br>ENSMUSG00000063801<br>ENSMUSG00000060708<br>ENSMUSG00000020198<br>ENSMUSG00000062444<br>ENSMUSG00000005804<br>ENSMUSG00000057506<br>ENSMUSG00000041670<br>ENSMUSG00000020848<br>ENSMUSG00000037386<br>ENSMUSG00000049630<br>ENSMUSG00000032216<br>ENSMUSG00000037890<br>ENSMUSG00000029112<br>ENSMUSG00000096146<br>ENSMUSG00000030314<br>ENSMUSG00000037580<br>ENSMUSG00000025579<br>ENSMUSG00000068079<br>ENSMUSG00000021983		

ENSMUSG00000031960<br>ENSMUSG00000030894<br>ENSMUSG00000057897<br>ENSMUSG00000028341<br>ENSMUSG00000022840<br>ENSMUSG00000008658<br>ENSMUSG00000022055<br>ENSMUSG00000028224<br>ENSMUSG00000031996<br>ENSMUSG00000020734<br>ENSMUSG00000040148<br>ENSMUSG00000018012<br>ENSMUSG00000009681<br>ENSMUSG00000062762<br>ENSMUSG00000074676<br>ENSMUSG00000033565<br>ENSMUSG00000020900<br>ENSMUSG00000015134<br>ENSMUSG00000017631<br>ENSMUSG00000048000<br>ENSMUSG00000032394<br>ENSMUSG00000038664<br>ENSMUSG00000020142<br>ENSMUSG00000068270<br>ENSMUSG00000041552<br>ENSMUSG00000030077<br>ENSMUSG00000031232<br>ENSMUSG00000039530<br>ENSMUSG00000050368<br>ENSMUSG00000005069<br>ENSMUSG00000036580<br>ENSMUSG00000042605<br>ENSMUSG00000022484<br>ENSMUSG00000062168<br>ENSMUSG00000038276<br>ENSMUSG00000051056<br>ENSMUSG00000056043<br>ENSMUSG00000024990<br>ENSMUSG00000037418<br>ENSMUSG00000029064<br>ENSMUSG00000096776<br>ENSMUSG00000096244<br>ENSMUSG00000095201<br>ENSMUSG00000096152<br>ENSMUSG00000096164<br>ENSMUSG00000094011<br>ENSMUSG00000094735<br>ENSMUSG00000094298<br>ENSMUSG00000094757<br>ENSMUSG00000091539<br>ENSMUSG00000095745<br>ENSMUSG00000095806<br>ENSMUSG00000096386<br>ENSMUSG00000094545<br>ENSMUSG00000094762<br>ENSMUSG00000096663<br>ENSMUSG00000096903<br>ENSMUSG00000096761<br>ENSMUSG00000095837<br>ENSMUSG00000095275<br>ENSMUSG00000095768<br>ENSMUSG00000094284<br>ENSMUSG00000091638<br>ENSMUSG00000094085<br>ENSMUSG00000091435<br>ENSMUSG00000095973<br>ENSMUSG00000096737<br>ENSMUSG00000094589<br>ENSMUSG00000094010<br>ENSMUSG00000093986<br>ENSMUSG00000094680<br>ENSMUSG00000094001<br>ENSMUSG00000094385<br>ENSMUSG00000094221<br>ENSMUSG00000095309<br>ENSMUSG00000096813<br>ENSMUSG00000093890		

ENSMUSG00000095758<br>ENSMUSG00000094879<br>ENSMUSG00000094208<br>ENSMUSG00000093941<br>ENSMUSG00000074322<br>ENSMUSG00000096513<br>ENSMUSG00000096760<br>ENSMUSG00000095064<br>ENSMUSG00000095191<br>ENSMUSG00000095962<br>ENSMUSG00000095163<br>ENSMUSG00000074311<br>ENSMUSG00000096304<br>ENSMUSG00000096348<br>ENSMUSG00000095273<br>ENSMUSG00000096071<br>ENSMUSG00000094981<br>ENSMUSG00000094149<br>ENSMUSG00000093871<br>ENSMUSG00000093917<br>ENSMUSG00000095543<br>ENSMUSG00000094542<br>ENSMUSG00000096871<br>ENSMUSG00000093853<br>ENSMUSG00000095383<br>ENSMUSG00000094748<br>ENSMUSG00000095984<br>ENSMUSG00000094905<br>ENSMUSG00000095081<br>ENSMUSG00000095619<br>ENSMUSG00000094700<br>ENSMUSG00000095931<br>ENSMUSG00000094931<br>ENSMUSG00000092297<br>ENSMUSG00000094532<br>ENSMUSG00000094934<br>ENSMUSG00000096601<br>ENSMUSG00000095358<br>ENSMUSG00000096073<br>ENSMUSG00000090715<br>ENSMUSG00000074291<br>ENSMUSG00000094602<br>ENSMUSG00000094187<br>ENSMUSG00000062483<br>ENSMUSG00000035523<br>ENSMUSG00000090411<br>ENSMUSG00000095632<br>ENSMUSG00000096859<br>ENSMUSG00000057513<br>ENSMUSG00000062598<br>ENSMUSG00000046924<br>ENSMUSG00000092473<br>ENSMUSG00000057946<br>ENSMUSG00000092456<br>ENSMUSG00000066723<br>ENSMUSG00000027985<br>ENSMUSG00000046130<br>ENSMUSG00000091924<br>ENSMUSG00000000216<br>ENSMUSG00000030873<br>ENSMUSG00000044248<br>ENSMUSG00000022996<br>ENSMUSG00000025739<br>ENSMUSG00000030340<br>ENSMUSG00000033470<br>ENSMUSG00000044338<br>ENSMUSG00000074402<br>ENSMUSG00000091874<br>ENSMUSG00000091652<br>ENSMUSG00000078808<br>ENSMUSG00000074401<br>ENSMUSG00000090794<br>ENSMUSG00000094313<br>ENSMUSG00000092579<br>ENSMUSG00000058631<br>ENSMUSG00000058399<br>ENSMUSG00000066850		

ENSMUSG00000045340<br>ENSMUSG00000059206<br>ENSMUSG00000095430<br>ENSMUSG00000051687<br>ENSMUSG00000047655<br>ENSMUSG00000043308<br>ENSMUSG00000045713<br>ENSMUSG00000095864<br>ENSMUSG00000061602<br>ENSMUSG00000096735<br>ENSMUSG00000057161<br>ENSMUSG00000094078<br>ENSMUSG00000066804<br>ENSMUSG00000066803<br>ENSMUSG00000070817<br>ENSMUSG00000070816<br>ENSMUSG00000070815<br>ENSMUSG00000095902<br>ENSMUSG00000095629<br>ENSMUSG00000057754<br>ENSMUSG00000061977<br>ENSMUSG00000051153<br>ENSMUSG00000063478<br>ENSMUSG00000059382<br>ENSMUSG00000071150<br>ENSMUSG00000078280<br>ENSMUSG00000071149<br>ENSMUSG00000060412<br>ENSMUSG00000058349<br>ENSMUSG00000030194<br>ENSMUSG00000056926<br>ENSMUSG00000059410<br>ENSMUSG00000063762<br>ENSMUSG00000057699<br>ENSMUSG00000062528<br>ENSMUSG00000030196<br>ENSMUSG00000097425<br>ENSMUSG00000045267<br>ENSMUSG00000091151<br>ENSMUSG00000043537<br>ENSMUSG00000042848<br>ENSMUSG00000060245<br>ENSMUSG00000061150<br>ENSMUSG00000045417<br>ENSMUSG00000050933<br>ENSMUSG00000062165<br>ENSMUSG00000045575<br>ENSMUSG00000057203<br>ENSMUSG00000054142<br>ENSMUSG00000058030<br>ENSMUSG00000057299<br>ENSMUSG00000046932<br>ENSMUSG00000095125<br>ENSMUSG00000069292<br>ENSMUSG00000069291<br>ENSMUSG00000094898<br>ENSMUSG00000094379<br>ENSMUSG00000094637<br>ENSMUSG00000071491<br>ENSMUSG00000071490<br>ENSMUSG00000061829<br>ENSMUSG00000057799<br>ENSMUSG00000060490<br>ENSMUSG00000061376<br>ENSMUSG00000069280<br>ENSMUSG00000094798<br>ENSMUSG00000095982<br>ENSMUSG00000024749<br>ENSMUSG00000060332<br>ENSMUSG00000001249<br>ENSMUSG00000024597<br>ENSMUSG00000022346<br>ENSMUSG00000054200<br>ENSMUSG00000028950<br>ENSMUSG00000079258<br>ENSMUSG00000040061<br>ENSMUSG00000038555		

ENSMUSG00000041730<br>ENSMUSG00000032769<br>ENSMUSG00000033717<br>ENSMUSG00000036251<br>ENSMUSG00000060216<br>ENSMUSG00000032537<br>ENSMUSG00000002393<br>ENSMUSG00000047557<br>ENSMUSG00000015533<br>ENSMUSG00000026249<br>ENSMUSG00000048349<br>ENSMUSG00000023828<br>ENSMUSG00000002341<br>ENSMUSG00000063063<br>ENSMUSG00000030272<br>ENSMUSG00000006699<br>ENSMUSG00000049176<br>ENSMUSG00000010803<br>ENSMUSG00000032012<br>ENSMUSG00000028273<br>ENSMUSG00000051136<br>ENSMUSG00000026304<br>ENSMUSG00000022607<br>ENSMUSG00000021994<br>ENSMUSG00000029245<br>ENSMUSG00000025020<br>ENSMUSG00000074736<br>ENSMUSG00000047085<br>ENSMUSG00000031654<br>ENSMUSG00000022674<br>ENSMUSG00000024647<br>ENSMUSG00000023885<br>ENSMUSG00000064177<br>ENSMUSG00000031253<br>ENSMUSG00000005958<br>ENSMUSG00000000632<br>ENSMUSG00000045471<br>ENSMUSG00000028161<br>ENSMUSG00000023052<br>ENSMUSG00000023942<br>ENSMUSG00000035200<br>ENSMUSG00000020484<br>ENSMUSG00000039579<br>ENSMUSG00000043051<br>ENSMUSG00000013033<br>ENSMUSG00000006398<br>ENSMUSG00000026883<br>ENSMUSG00000026965<br>ENSMUSG00000024268<br>ENSMUSG00000043895<br>ENSMUSG00000028004<br>ENSMUSG00000052921		

Gene list for ubiquitous GO terms for mouse

Membrane organization	Protein folding	RNA splicing
ENSMUSG00000061455	ENSMUSG00000031960	ENSMUSG00000008373
ENSMUSG00000030720	ENSMUSG00000039515	ENSMUSG00000001767
ENSMUSG00000050732	ENSMUSG00000025757	ENSMUSG00000033228
ENSMUSG00000022765	ENSMUSG00000020048	ENSMUSG00000062604
ENSMUSG00000066621	ENSMUSG00000083780	ENSMUSG00000019432
ENSMUSG00000030365	ENSMUSG00000001555	ENSMUSG00000032580
ENSMUSG00000020143	ENSMUSG00000062933	ENSMUSG00000055760
ENSMUSG00000033788	ENSMUSG00000015656	ENSMUSG00000060121
ENSMUSG00000042828	ENSMUSG00000025613	ENSMUSG00000024735
ENSMUSG00000033196	ENSMUSG00000048330	ENSMUSG00000032469
ENSMUSG00000048612	ENSMUSG00000020635	ENSMUSG00000037197
ENSMUSG00000062694	ENSMUSG00000036052	ENSMUSG00000005150
ENSMUSG00000020900	ENSMUSG00000027804	ENSMUSG00000025982
ENSMUSG00000024743	ENSMUSG00000030007	ENSMUSG00000020409
ENSMUSG00000003873	ENSMUSG00000019428	ENSMUSG00000020211
ENSMUSG00000022144	ENSMUSG00000002732	ENSMUSG00000028809
ENSMUSG00000041189	ENSMUSG00000055795	ENSMUSG00000028676
ENSMUSG00000022789	ENSMUSG00000071866	ENSMUSG00000035585
ENSMUSG00000047205	ENSMUSG00000028343	ENSMUSG00000024268

ENSMUSG00000025812	ENSMUSG00000003226	ENSMUSG00000059208
ENSMUSG00000066319	ENSMUSG00000099102	ENSMUSG00000046434
ENSMUSG00000033039	ENSMUSG00000024041	ENSMUSG00000027763
ENSMUSG00000017774	ENSMUSG00000005681	ENSMUSG00000034544
ENSMUSG00000015950	ENSMUSG00000032171	ENSMUSG00000028820
ENSMUSG00000025043	ENSMUSG00000006412	ENSMUSG00000041297
ENSMUSG00000022722	ENSMUSG00000024359	ENSMUSG00000023110
ENSMUSG00000025220	ENSMUSG00000019732	ENSMUSG00000024217
ENSMUSG00000020882	ENSMUSG00000005981	ENSMUSG00000060098
ENSMUSG00000033355	ENSMUSG00000024346	ENSMUSG00000040621
ENSMUSG00000042429	ENSMUSG00000066151	ENSMUSG00000054679
ENSMUSG00000030654	ENSMUSG00000031197	ENSMUSG00000028484
ENSMUSG00000027828	ENSMUSG00000001289	ENSMUSG00000018379
ENSMUSG00000025780	ENSMUSG00000004069	ENSMUSG00000098301
ENSMUSG00000009549	ENSMUSG00000030877	ENSMUSG00000002129
ENSMUSG00000014504	ENSMUSG00000015757	ENSMUSG00000040383
ENSMUSG00000030082	ENSMUSG00000007739	ENSMUSG00000004096
ENSMUSG00000020780	ENSMUSG00000028558	ENSMUSG00000061479
ENSMUSG00000078598	ENSMUSG00000020698	ENSMUSG00000005687
ENSMUSG00000010066	ENSMUSG00000043510	ENSMUSG00000090115
ENSMUSG00000026511	ENSMUSG00000038074	ENSMUSG00000029817
ENSMUSG00000032042	ENSMUSG00000052033	ENSMUSG00000005481
ENSMUSG00000021039	ENSMUSG00000021868	ENSMUSG00000021111
ENSMUSG00000031314	ENSMUSG00000002190	ENSMUSG00000021039
ENSMUSG00000036323	ENSMUSG00000005483	ENSMUSG00000063511
ENSMUSG00000073079	ENSMUSG00000003355	ENSMUSG00000020850
ENSMUSG00000079108	ENSMUSG00000032966	ENSMUSG00000021047
ENSMUSG00000021020	ENSMUSG00000026740	ENSMUSG00000023932
ENSMUSG00000019802	ENSMUSG00000025130	ENSMUSG00000031723
ENSMUSG00000032725	ENSMUSG00000024538	ENSMUSG00000002455
ENSMUSG00000001827	ENSMUSG00000029781	ENSMUSG00000071273
ENSMUSG00000028649	ENSMUSG00000023004	ENSMUSG00000008333
ENSMUSG00000027706	ENSMUSG00000030421	ENSMUSG00000061360
ENSMUSG00000029468	ENSMUSG00000024580	ENSMUSG00000015748
ENSMUSG00000028125	ENSMUSG00000028410	ENSMUSG00000022858
ENSMUSG00000029684	ENSMUSG00000003814	ENSMUSG00000090553
ENSMUSG00000000058	ENSMUSG00000032060	ENSMUSG00000074817
ENSMUSG00000051412	ENSMUSG00000021037	ENSMUSG00000029823
ENSMUSG00000030279	ENSMUSG00000040554	ENSMUSG00000002107
ENSMUSG00000036499	ENSMUSG00000039615	ENSMUSG00000024188
ENSMUSG00000000001	ENSMUSG00000073676	ENSMUSG00000028134
ENSMUSG00000033721	ENSMUSG00000031701	ENSMUSG00000020863
ENSMUSG00000020903	ENSMUSG00000024847	ENSMUSG00000005506
ENSMUSG00000005804	ENSMUSG00000024309	ENSMUSG00000001783
ENSMUSG00000026470	ENSMUSG00000027472	ENSMUSG00000020781
ENSMUSG00000015243	ENSMUSG00000026035	ENSMUSG00000024713
ENSMUSG00000098112	ENSMUSG00000060288	ENSMUSG00000027079
ENSMUSG00000026656	ENSMUSG00000001525	ENSMUSG00000042389
ENSMUSG00000059498	ENSMUSG00000028960	ENSMUSG00000047656
ENSMUSG00000058715	ENSMUSG00000032285	ENSMUSG00000014980
ENSMUSG00000037902	ENSMUSG00000024184	ENSMUSG00000037958
ENSMUSG00000056870	ENSMUSG00000028651	ENSMUSG00000031590
ENSMUSG00000024397	ENSMUSG00000042133	ENSMUSG00000025576
ENSMUSG00000079293	ENSMUSG00000021270	ENSMUSG00000040824
ENSMUSG00000030605	ENSMUSG00000079480	ENSMUSG00000094658
ENSMUSG00000041112	ENSMUSG00000050612	ENSMUSG00000093918
ENSMUSG00000015947	ENSMUSG00000038044	ENSMUSG00000093987
ENSMUSG00000001847	ENSMUSG00000028035	ENSMUSG00000095948
ENSMUSG00000028063	ENSMUSG00000001416	ENSMUSG00000096520
ENSMUSG00000019873	ENSMUSG00000034024	ENSMUSG00000039449
ENSMUSG00000026849	ENSMUSG00000030357	ENSMUSG00000022635
ENSMUSG00000063450	ENSMUSG00000022771	ENSMUSG00000044709
ENSMUSG00000033589	ENSMUSG00000039230	ENSMUSG00000027593
ENSMUSG00000043207	ENSMUSG00000029131	ENSMUSG00000061028
ENSMUSG00000022142	ENSMUSG00000068039	ENSMUSG00000010608
ENSMUSG00000042524	ENSMUSG00000029198	ENSMUSG00000029290
ENSMUSG00000030409	ENSMUSG00000038503	ENSMUSG00000053453
ENSMUSG00000018559	ENSMUSG00000024222	ENSMUSG00000025580
ENSMUSG00000036817	ENSMUSG00000023944	ENSMUSG00000009076
ENSMUSG00000047363	ENSMUSG00000032525	ENSMUSG00000015656
ENSMUSG00000004748	ENSMUSG00000029447	ENSMUSG00000059005
ENSMUSG00000026317	ENSMUSG00000039233	ENSMUSG00000034274
ENSMUSG00000003438	ENSMUSG00000020368	ENSMUSG00000029279
ENSMUSG00000005069	ENSMUSG00000022234	ENSMUSG00000063919
ENSMUSG00000029020	ENSMUSG00000022403	ENSMUSG00000011306
ENSMUSG00000025889	ENSMUSG00000030708	ENSMUSG00000033222
ENSMUSG00000038084	ENSMUSG00000009030	ENSMUSG00000022774

ENSMUSG00000053768	ENSMUSG00000024007	ENSMUSG00000024287
ENSMUSG00000022346	ENSMUSG00000056629	ENSMUSG00000036054
ENSMUSG00000069601	ENSMUSG00000021713	ENSMUSG00000044155
ENSMUSG00000026532	ENSMUSG00000020949	ENSMUSG00000002477
ENSMUSG00000021061	ENSMUSG00000021715	ENSMUSG00000061136
ENSMUSG00000041936	ENSMUSG00000078451	ENSMUSG00000003660
ENSMUSG00000020315	ENSMUSG00000015357	ENSMUSG00000037361
ENSMUSG00000047878	ENSMUSG00000021831	ENSMUSG00000059708
ENSMUSG00000063681	ENSMUSG00000032383	ENSMUSG00000063166
ENSMUSG00000029501	ENSMUSG00000040013	ENSMUSG00000025872
ENSMUSG00000029682	ENSMUSG00000001134	ENSMUSG00000031848
ENSMUSG00000029678	ENSMUSG00000004460	ENSMUSG00000029169
ENSMUSG00000072647	ENSMUSG00000033684	ENSMUSG00000057278
ENSMUSG00000029344	ENSMUSG00000097319	ENSMUSG00000028609
ENSMUSG00000030342	ENSMUSG00000036430	ENSMUSG00000061613
ENSMUSG00000064158	ENSMUSG00000055936	ENSMUSG00000001158
ENSMUSG00000038498	ENSMUSG00000034203	ENSMUSG00000025024
ENSMUSG00000028575	ENSMUSG00000027006	ENSMUSG00000028382
ENSMUSG00000046846	ENSMUSG00000025980	ENSMUSG00000043639
ENSMUSG00000043648	ENSMUSG00000060802	ENSMUSG00000002524
ENSMUSG00000031967	ENSMUSG00000021236	ENSMUSG00000022139
ENSMUSG00000024527	ENSMUSG00000037072	ENSMUSG00000063800
ENSMUSG00000057789	ENSMUSG00000037062	ENSMUSG00000030056
ENSMUSG00000022412	ENSMUSG00000026848	ENSMUSG00000027998
ENSMUSG00000026150	ENSMUSG00000026849	ENSMUSG00000022961
ENSMUSG00000027668	ENSMUSG00000029657	ENSMUSG00000040728
ENSMUSG00000019054	ENSMUSG00000024610	ENSMUSG00000040720
ENSMUSG00000067851	ENSMUSG00000009563	ENSMUSG00000004980
ENSMUSG00000074582	ENSMUSG00000037649	ENSMUSG00000066148
ENSMUSG00000041685	ENSMUSG00000037548	ENSMUSG00000064317
ENSMUSG00000045210	ENSMUSG00000079547	ENSMUSG00000060373
ENSMUSG00000024191	ENSMUSG00000059555	ENSMUSG00000027014
ENSMUSG00000064210	ENSMUSG00000060519	ENSMUSG00000037070
ENSMUSG00000032369	ENSMUSG00000001774	ENSMUSG00000031683
ENSMUSG00000062300	ENSMUSG00000022037	ENSMUSG00000069014
ENSMUSG00000031451	ENSMUSG00000003464	ENSMUSG00000007050
ENSMUSG00000010047	ENSMUSG00000048206	ENSMUSG00000027620
ENSMUSG00000031628	ENSMUSG00000018845	ENSMUSG00000027905
ENSMUSG00000053647	ENSMUSG00000030533	ENSMUSG00000078184
ENSMUSG00000074029	ENSMUSG00000071072	ENSMUSG00000021413
ENSMUSG00000022770	ENSMUSG00000014195	ENSMUSG00000008658
ENSMUSG00000028657		ENSMUSG00000029345
ENSMUSG00000001018		ENSMUSG00000078765
ENSMUSG00000033579		ENSMUSG00000039630
ENSMUSG00000013663		ENSMUSG00000029701
ENSMUSG00000022982		ENSMUSG00000078348
ENSMUSG00000004056		ENSMUSG00000037475
ENSMUSG00000021112		ENSMUSG00000020018
ENSMUSG00000045629		ENSMUSG00000091625
ENSMUSG00000001729		ENSMUSG00000045365
ENSMUSG00000005125		ENSMUSG00000027404
ENSMUSG00000025809		ENSMUSG00000027510
ENSMUSG00000038555		ENSMUSG00000063543
ENSMUSG00000047139		ENSMUSG00000042502
ENSMUSG00000032826		ENSMUSG00000034192
ENSMUSG00000028854		ENSMUSG00000033712
ENSMUSG00000021248		ENSMUSG00000019470
ENSMUSG00000035069		ENSMUSG00000025134
ENSMUSG00000009995		ENSMUSG00000021431
ENSMUSG00000030086		ENSMUSG00000046201
ENSMUSG00000005299		ENSMUSG00000021134
ENSMUSG00000023043		ENSMUSG00000023007
ENSMUSG00000026696		ENSMUSG00000056962
ENSMUSG00000026672		ENSMUSG00000034120
ENSMUSG00000079657		ENSMUSG00000041459
ENSMUSG00000026577		ENSMUSG00000056305
ENSMUSG00000073002		ENSMUSG00000021494
ENSMUSG00000056515		ENSMUSG00000034931
ENSMUSG00000006464		ENSMUSG00000021500
ENSMUSG00000031755		ENSMUSG00000040767
ENSMUSG00000022200		ENSMUSG00000007670
ENSMUSG00000038708		ENSMUSG00000038406
ENSMUSG00000028955		ENSMUSG00000029538
ENSMUSG00000020894		ENSMUSG00000057130
ENSMUSG00000038671		ENSMUSG00000020180
ENSMUSG00000020671		ENSMUSG00000026035
ENSMUSG00000003378		ENSMUSG00000028639

ENSMUSG00000027253		ENSMUSG00000060288
ENSMUSG00000057280		ENSMUSG00000031311
ENSMUSG00000031328		ENSMUSG00000049124
ENSMUSG00000029071		ENSMUSG00000033565
ENSMUSG00000040003		ENSMUSG00000021546
ENSMUSG00000034656		ENSMUSG00000027881
ENSMUSG00000062352		ENSMUSG00000052144
ENSMUSG00000028969		ENSMUSG00000037296
ENSMUSG00000067629		ENSMUSG00000029427
ENSMUSG00000044716		ENSMUSG00000028651
ENSMUSG00000030774		ENSMUSG00000096370
ENSMUSG00000024044		ENSMUSG00000033760
ENSMUSG00000045382		ENSMUSG00000094936
ENSMUSG00000040152		ENSMUSG00000036109
ENSMUSG00000037752		ENSMUSG00000028137
ENSMUSG00000033191		ENSMUSG00000028902
ENSMUSG00000009214		ENSMUSG00000039148
ENSMUSG00000021079		ENSMUSG00000050188
ENSMUSG00000027076		ENSMUSG00000062078
ENSMUSG00000022437		ENSMUSG00000043962
ENSMUSG00000021270		ENSMUSG00000029402
ENSMUSG00000022427		ENSMUSG00000031792
ENSMUSG00000020361		ENSMUSG00000030435
ENSMUSG00000067825		ENSMUSG00000030512
ENSMUSG00000003464		ENSMUSG00000031134
ENSMUSG00000027222		ENSMUSG00000020929
ENSMUSG00000019809		ENSMUSG00000060475
ENSMUSG00000036943		ENSMUSG00000028180
ENSMUSG00000052456		ENSMUSG00000014776
ENSMUSG00000026837		ENSMUSG00000030188
ENSMUSG00000059834		ENSMUSG00000031189
ENSMUSG00000011751		ENSMUSG00000074088
ENSMUSG00000046167		ENSMUSG00000003527
ENSMUSG00000020598		ENSMUSG00000092607
ENSMUSG00000026442		ENSMUSG00000035215
ENSMUSG00000039419		ENSMUSG00000029439
ENSMUSG00000025221		ENSMUSG00000084128
ENSMUSG00000053024		ENSMUSG00000004865
ENSMUSG00000054342		ENSMUSG00000030204
ENSMUSG00000003341		ENSMUSG00000028882
ENSMUSG00000032328		ENSMUSG00000007850
ENSMUSG00000006782		ENSMUSG00000041777
ENSMUSG00000021868		ENSMUSG00000021645
ENSMUSG00000074170		ENSMUSG00000024949
ENSMUSG00000024521		ENSMUSG00000030216
ENSMUSG00000007659		ENSMUSG00000028821
ENSMUSG00000078566		ENSMUSG00000033732
ENSMUSG00000029104		ENSMUSG00000071172
ENSMUSG00000024959		ENSMUSG00000022283
ENSMUSG00000057329		ENSMUSG00000024097
ENSMUSG00000000631		ENSMUSG00000024007
ENSMUSG00000037643		ENSMUSG00000030224
ENSMUSG00000058569		ENSMUSG00000034889
ENSMUSG00000026924		ENSMUSG00000038446
ENSMUSG00000004364		ENSMUSG00000032621
ENSMUSG00000026455		ENSMUSG00000041319
ENSMUSG00000026589		ENSMUSG00000063480
ENSMUSG00000003037		ENSMUSG00000039218
ENSMUSG00000021567		ENSMUSG00000068856
ENSMUSG00000070000		ENSMUSG00000021713
ENSMUSG00000028552		ENSMUSG00000021716
ENSMUSG00000035152		ENSMUSG00000038374
ENSMUSG00000020297		ENSMUSG00000042079
ENSMUSG00000022150		ENSMUSG00000034681
ENSMUSG00000025468		ENSMUSG00000032940
ENSMUSG00000006169		ENSMUSG00000003119
ENSMUSG00000029126		ENSMUSG00000016018
ENSMUSG00000039361		ENSMUSG00000037275
ENSMUSG00000039959		ENSMUSG00000078676
ENSMUSG00000033419		ENSMUSG00000032423
ENSMUSG00000032757		ENSMUSG00000022023
ENSMUSG00000029407		ENSMUSG00000028330
ENSMUSG00000010110		ENSMUSG00000024604
ENSMUSG00000020198		ENSMUSG00000032040
ENSMUSG00000020745		ENSMUSG00000020715
ENSMUSG00000018736		
ENSMUSG00000035505		

ENSMUSG00000000959<br>ENSMUSG00000052852<br>ENSMUSG00000035745<br>ENSMUSG00000033383<br>ENSMUSG00000047531<br>ENSMUSG00000026575<br>ENSMUSG00000037890<br>ENSMUSG00000001761<br>ENSMUSG00000030323<br>ENSMUSG00000026966<br>ENSMUSG00000041523<br>ENSMUSG00000014077<br>ENSMUSG00000032740<br>ENSMUSG00000052468<br>ENSMUSG00000041607<br>ENSMUSG00000033192<br>ENSMUSG00000018126<br>ENSMUSG00000041488<br>ENSMUSG00000021814<br>ENSMUSG00000039703<br>ENSMUSG00000025473<br>ENSMUSG00000030534<br>ENSMUSG00000022303<br>ENSMUSG00000038822<br>ENSMUSG00000056962<br>ENSMUSG00000028115<br>ENSMUSG00000007655<br>ENSMUSG00000016664<br>ENSMUSG00000004069<br>ENSMUSG00000013089<br>ENSMUSG00000026848<br>ENSMUSG00000026466<br>ENSMUSG00000015290<br>ENSMUSG00000024392<br>ENSMUSG00000025858<br>ENSMUSG00000037852<br>ENSMUSG00000040711<br>ENSMUSG00000079110<br>ENSMUSG00000040276<br>ENSMUSG00000020216<br>ENSMUSG00000037712<br>ENSMUSG00000049281<br>ENSMUSG00000041046<br>ENSMUSG00000028603<br>ENSMUSG00000001240<br>ENSMUSG00000048483<br>ENSMUSG00000027935<br>ENSMUSG00000027765<br>ENSMUSG00000045967<br>ENSMUSG00000044279<br>ENSMUSG00000059714<br>ENSMUSG00000037316<br>ENSMUSG00000036304<br>ENSMUSG00000038024<br>ENSMUSG00000031137<br>ENSMUSG00000074364<br>ENSMUSG00000000303<br>ENSMUSG00000018820<br>ENSMUSG00000004771<br>ENSMUSG00000070337<br>ENSMUSG00000034353<br>ENSMUSG00000029053<br>ENSMUSG00000022878<br>ENSMUSG00000034593<br>ENSMUSG00000021466<br>ENSMUSG00000029359<br>ENSMUSG00000035863<br>ENSMUSG00000031543<br>ENSMUSG00000024855<br>ENSMUSG00000029390<br>ENSMUSG00000028126<br>ENSMUSG00000024048<br>ENSMUSG00000021143<br>ENSMUSG00000006127<br>ENSMUSG00000024065<br>ENSMUSG00000032231<br>ENSMUSG00000015647		

ENSMUSG00000036856<br>ENSMUSG00000038298<br>ENSMUSG00000004446<br>ENSMUSG00000019787<br>ENSMUSG00000002059<br>ENSMUSG00000079477<br>ENSMUSG00000055069<br>ENSMUSG00000031504<br>ENSMUSG00000021486<br>ENSMUSG00000029535<br>ENSMUSG00000024109<br>ENSMUSG00000074736<br>ENSMUSG00000063887<br>ENSMUSG00000031302<br>ENSMUSG00000051790<br>ENSMUSG00000028399<br>ENSMUSG00000020886<br>ENSMUSG00000022623<br>ENSMUSG00000042453<br>ENSMUSG00000031760<br>ENSMUSG00000027257<br>ENSMUSG00000032733<br>ENSMUSG00000017897<br>ENSMUSG00000017686<br>ENSMUSG00000025733<br>ENSMUSG00000086965<br>ENSMUSG00000057506<br>ENSMUSG00000039164<br>ENSMUSG00000028145<br>ENSMUSG00000059552<br>ENSMUSG00000027104<br>ENSMUSG00000027381<br>ENSMUSG00000028455		
